# OsAPSE modulates non-covalent interactions between arabinogalactan protein *O*-glycans and pectin in rice cell walls

**DOI:** 10.3389/fpls.2025.1588802

**Published:** 2025-05-22

**Authors:** Tibo De Coninck, Isabel Verbeke, Pierre Rougé, Tom Desmet, Els J. M. Van Damme

**Affiliations:** ^1^ Department of Biotechnology, Laboratory for Biochemistry & Glycobiology, Ghent University, Ghent, Belgium; ^2^ Unité Mixte de Recherche (UMR) 152 PharmaDev, Université Toulouse III Paul Sabatier, Institut de Recherche et Développement, Toulouse, France; ^3^ Department of Biotechnology, Centre for Synthetic Biology, Ghent University, Ghent, Belgium

**Keywords:** cell wall, glycoside hydrolase, α-D-galactopyranosidase, β-l-arabinopyranosidase, *Oryza sativa*, rice, arabinogalactan protein, germination

## Abstract

Flexibility of cell walls is crucial to accommodate cell elongation and growth, typically associated with the reorganization of cell wall polysaccharides. Seed germination is a fast-paced developmental process in which cell wall adaptability is highly required. The plant cell utilizes multiple strategies to obtain a flexible cell wall and in part relies on cell wall-active enzymes to loosen both covalent and non-covalent interactions between cell wall polysaccharides. OsAPSE is an example of a cell wall-active enzyme originating from Japanese rice (*Oryza sativa* subsp. Japonica) belonging to the glycoside hydrolase family 27 (GH27), potentially active on the pectin–arabinogalactan protein *O*-glycan junction. We provide insights into the biochemical and enzymatic properties of this protein, characterized by the presence of a GH27 domain linked to a ricin-B-like domain. Using small-scale production experiments in a cell-free protein synthesis system, we demonstrated the catalytic activity of the recombinant OsAPSE towards synthetic and natural substrates. Furthermore, subcellular localization analysis and *in silico* data suggest that OsAPSE may undergo unconventional secretion to the cell surface. We hypothesize that OsAPSE plays a role during rice seed germination by removing terminal α-D-Gal*p* and β-L-Ara*p* moieties along the pectin–arabinogalactan protein *O*-glycan network. This activity may abolish non-covalent interactions between pectic rhamnogalacturonan I and *O*-glycans of arabinogalactan proteins, contributing to cell wall relaxation for growth during germination.

## 1 Introduction

Glycoside hydrolases (GHs) are carbohydrate-active enzymes (CAZymes), catalyzing the hydrolytic cleavage of glycosidic bonds (Henrissat and Davies, 1997). Today, 1.9 million modules are classified in almost 190 GH families in the CAZy database (Drula et al., 2022). Members from the same GH family are evolutionarily related, show a conserved protein structure and act mechanistically similar on substrates.

The GH family of interest in this study is the GH27 family, which is present in every kingdom of life (Naumoff, 2004). Members of the GH27 family can display several activities, including α-D-galactopyranosidase (AGAL)/melibiase, N-acetylgalactosaminidase (NAGA) or β-L-arabinopyranosidase (ARAP) activity. In general, *bona fide* GH27 enzymes catalyze the hydrolysis of glycosidic bonds between α-D-galactopyranosyl (α-D-Gal*p*), α-1,3-N-acetyl-D-galactosaminyl (α-D-GalNAc) and/or β-L-arabinopyranosyl (β-L-Ara*p*) residues and other carbohydrates in a wide range of substrates (**Supplementary File S1**). Several GH27 enzymes are bifunctional proteins and display both AGAL and ARAP activity (Sakamoto et al., 2010; Kotake et al., 2016; Imaizumi et al., 2017; Kikuchi et al., 2017). This property is attributed to the structural similarities between α-D-Gal*p* and β-L-Ara*p* (Kotake et al., 2016), but also to the presence of conserved residues in the catalytic pocket of GH27 enzymes (Imaizumi et al., 2017) that make use of the Koshland double displacement mechanism and retain the anomeric configuration of the substrate upon hydrolysis (McCarter and Stephen Withers, 1994). The catalytic residues are aspartic acid residues and are strongly conserved within the GH27 family (Zhu et al., 1995; Hart et al., 2000; Ly et al., 2000; Garman et al., 2002; Fujimoto et al., 2003; Guce et al., 2010; Okazawa et al., 2015; Kytidou et al., 2018). Non-canonical activities have been reported sporadically, including glucan-α-1,6-isomaltosidase and galactan:galactosyltransferase activity (**Supplementary File S1**). These aforementioned activities, whether or not canonical, have also been observed in other GH families. Families GH27, GH31 and GH36 constitute the GH-D clan, a GH superfamily with mechanistic and structural resemblances (Comfort et al., 2007). In eukaryotes, the canonical activities are confined to the GH-D clan, while in prokaryotes these activities are also found in families outside the GH-D clan, *i.e.* in GH4, GH31, GH57, GH97, GH109, GH110 and GH129.

GH27 enzymes are of interest for various applications (Katrolia et al., 2014). In human medicine, several debilitating disorders, including Fabry, Schindler and Kanzaki disease, are associated with mutations in AGAL and NAGA genes, causing accumulation of glycosphingolipids and glycoproteins (Garman et al., 2002; Guce et al., 2010). Enzyme replacement therapy and gene therapy are employed to treat the aforementioned diseases (Kytidou et al., 2018; Umer and Kalra, 2023). Furthermore, NAGA and AGAL can be used to convert blood type A and B antigens respectively, to the universal donor type O blood (Rahfeld and Withers, 2020). In animal feed industry, AGALs are used to degrade raffinose family oligosaccharides (RFOs) in legumes, since non-ruminants are unable to digest RFOs (Di Stefano et al., 2007; Elango et al., 2022). RFOs are fermented by gut bacteria, causing abdominal discomfort, flatulence and diarrhea (Mutuyemungu et al., 2023). In plants, GH27 enzymes have been implicated in several developmental processes including seed germination (Guimaraes et al., 2001; Blöchl et al., 2008; Jia et al., 2015; Lien et al., 2018; Arunraj et al., 2020; Zhang et al., 2021a; Okazawa et al., 2022; Gojło, 2023), fruit development (Soh et al., 2006; Tsaniklidis et al., 2016; Hua et al., 2021; Liu et al., 2022), and senescence (Chrost et al., 2006; Lee et al., 2009; Zhang et al., 2021b), but also in the response towards biotic (Evers et al., 2006) and abiotic stresses (Pennycooke et al., 2003; Tapernoux-Lüthi et al., 2004; Zhao et al., 2006; Gu et al., 2018; Chen et al., 2023). The physiological roles for GH27 enzymes are multifarious and mostly associated with AGAL/ARAP-mediated degradation of storage oligosaccharides/polysaccharides (*i.e.* RFOs, galactomannan) or modification of structural glycoconjugates (*i.e.* galactolipids, *O*-glycans of arabinogalactan proteins (AGP)).

GH domains occur often in combination with a carbohydrate-recognition domain (CRD), which supports their function as a catalyst by enhancing substrate binding (Boraston et al., 2004). In plants, GH27 sequences often encode multidomain proteins in which the catalytic domain is coupled to a carbohydrate binding module (CBM) of family 13 or a ricin-B(-like) domain (Van Holle et al., 2017; Van Holle and Van Damme, 2019; De Coninck et al., 2024b).

The subject of this study is OsAPSE, a GH27 enzyme from Japanese rice (*O. sativa* subsp. Japonica), which was named after its characterized homolog AtAPSE from *Arabidopsis thaliana* (Imaizumi et al., 2017). The goal of this study is to provide clues about the enzymatic properties of the GH27 domain towards synthetic and natural substrates, the biological function of this bifunctional enzyme in relation to seed germination and cell wall metabolism, and its occurrence and phylogeny in the plant kingdom.

## 2 Materials and methods

### 2.1 Cloning, protein production and analysis

#### 2.1.1 Cloning of the GH27 domain of OsAPSE

The native coding sequence of the GH27 domain, flanked by 5’ *Nco*I and 3’ *Kpn*I restriction sites, an *N*-terminal His_6_-tag and double stop codon, was synthetically produced and cloned into a shuttle vector using the GeneArt Gene Synthesis service (Thermo Fisher Scientific, Waltham (MA), USA). The GH27 domain of *OsAPSE* was cloned into the *pALiCE02* expression vector for cell-free protein production (LenioBio GmbH, Düsseldorf, Germany) by means of a double restriction digest using 5 µg shuttle vector or 5 µg expression vector, 2.5 U *Nco*I and 2.5 U *KpnI* in 10X rCutSmart buffer (New England Biolabs, Ipswich (MA), USA) for 1 hour at 37°C, and 20 min heat inactivation of the restriction enzymes at 80°C. The double digests were purified using the QIAquick^®^ PCR & Gel Cleanup Kit (Qiagen, Hilden, Germany). The GH27 insert was ligated into the expression vector in a 3/1 insert-to-plasmid ratio using 5 U T4 DNA ligase (Thermo Fischer Scientific), 0.5 mM dithiothreitol (Thermo Fisher Scientific) and 10X ligase buffer (Thermo Fisher Scientific). The resulting expression plasmid was transformed into heat-shock competent *Escherichia coli* TOP10 cells (Thermo Fisher Scientific). Putatively transformed colonies were selected on lysogeny broth agar plates containing 80 µg/mL carbenicillin (Duchefa Biochemie, Haarlem, The Netherlands) and analyzed by colony PCR using Taq DNA polymerase (VWR, Radnor (PA), USA) and gene-specific primers (**Supplementary File S2**), with 5 min initial denaturation at 95°C, 35 cycles (30s at 95°C, 30s at 53°C, 1 min at 72°C) and 5 min final elongation at 72°C. Transformed TOP10 cells were propagated in lysogeny broth with 80 µg/mL carbenicillin and plasmids were purified at ultra-high purity using the NucleoBond Xtra Midi kit (Macherey-Nagel, Düren, Germany). Finally, the recombinant expression vector was analyzed by Sanger sequencing (Biosearch/LGC Genomics GmbH, Berlin, Germany) with plasmid-specific primers (**Supplementary File S2**).

#### 2.1.2 Cell-free production of the GH27 domain of OsAPSE

Protein synthesis was executed using the ‘Almost Living Cell-free Expression’ (ALiCE) cell-free production system (CFPS). Multiple reactions were initiated, by adding 500 ng of purified *pALiCE02*::GH27_OsAPSE per reaction at a final volume of 50 µL. Reactions with the *pALiCE02* empty vector were used as a control. After 48 hours of incubation at 25°C and 700 rpm on a thermomixer, the produced proteins were collected as described by the manufacturer’s protocol (Buntru et al., 2022). Due to the small scale of the CFPS reactions, no further purification was undertaken.

#### 2.1.3 Protein analysis

Protein concentrations were determined using the Bradford assay (Bio-Rad, Hercules (CA), USA) (Bradford, 1976) with bovine serum albumin (BSA) (MP Biomedicals, Irvine (CA), USA) as reference protein (0–1 mg/mL) in 96-well plates using a TECAN Infinite 200 PRO (TECAN, Männedorf, Switzerland) plate reader.

Discontinuous acrylamide gels containing 0.01% SDS (MP Biomedicals) and different concentrations of acrylamide/bisacrylamide ROTIPHORESE^®^ Gel 30 (37.5:1) (Carl Roth GmbH, Karlsruhe, Germany) in the stacking gel (pH 6.8, 4% acrylamide) and separating gel (pH 8.8, 15% acrylamide) respectively, were prepared. Polymerization was initiated with TEMED (Carl Roth GmbH) and 10 V% ammonium persulfate (Thermo Fisher Scientific). Protein samples were heat-treated (98°C) for 10 minutes with 4X sample buffer containing 1 M Tris-HCl pH 6.8 (MP Biomedicals), 8% SDS (MP Biomedicals), 40% glycerol (Chem-Lab), 0.4% bromophenol blue (Sigma-Aldrich, Saint Louis (MO), USA) and 1.125 M 2-mercaptoethanol (Sigma-Aldrich). Proteins were analyzed in a continuous electric field (180 V) for 1 hour in the presence of running buffer containing 25 mM Tris, 200 mM glycine (MP Biomedicals) and 0.1% SDS using a Mini-PROTEAN Tetra cell (Bio-Rad). Afterwards, acrylamide gels were stained with acidic Coomassie solution containing 0.1% Coomassie Brilliant Blue R250 (Merck, Darmstadt, Germany), 2.9 M glacial acetic acid (Chem-Lab) and 10.2 M HPLC-grade methanol (Chem-Lab), and destained with acidic destaining solution, containing 2.5 M technical ethanol (Chem-Lab) and 1.3 M glacial acetic acid for 2–3 hours.

Western blotting on methanol-activated Amersham Hybond™-P PVDF membranes (GE Healthcare, Chicago (IL), USA) was performed by semi-dry electroblotting (Bio-Rad) in Towbin buffer containing 25 mM Tris, 2.45 M HPLC-grade methanol and 192 mM glycine. After blotting, the membranes were incubated in 5% non-fat milk powder solution (AppliChem GmbH, Darmstadt, Germany). Immunodetection was executed with subsequent incubation steps (1 hour at room temperature) in consecutively 1/5000 THE™ His-tag monoclonal antibody (GenScript, Piscataway (NJ), USA), 1/1000 polyclonal rabbit anti-mouse antibody conjugated with horseradish peroxidase (Agilent/DAKO, Santa Clara (CA), USA), 1/300 peroxidase anti-peroxidase antibody (Sigma-Aldrich) and final detection in 100 mM Tris-HCl pH 7.6 buffer containing 1 mM 3,3’-diaminobenzidine (DAB) (Thermo Fisher Scientific) and 320 µM H_2_O_2_ (Acros Organics, Geel, Belgium). Trissaline containing 10 mM Tris, 150 mM NaCl (Chem-Lab) and 0.1 V% Triton-X100 (Sigma Aldrich) was used as diluent for all antibodies and for membrane washes (3x5 min) in between antibody incubations.

### 2.2 Enzymatic assays

#### 2.2.1 Experimental set-up

Different experimental set-ups were applied for the enzymatic assays including the initial screening for AGAL and ARAP activity, determination of the pH/temperature optima, determination of K_M_ and V_max_ and the activity on natural substrates (**Table 1**). The pH/temperature optima and enzymatic characteristics were determined using synthetic substrates, *i.e.* pNP-α-D-Gal*p* and pNP-β-L-Ara*p* (Sigma-Aldrich) and detection through absorbance measurements at 405 ± 10 nm using a TECAN Infinite 200 PRO plate reader. Activity assays on natural substrates made use of 50 mM melibiose monohydrate (Merck), 50 mM raffinose pentahydrate (Sigma-Aldrich), 50 mM verbascose (Megazyme, Wicklow, Ireland), 2.5 mg/mL arabinogalactan from larch wood (Sigma-Aldrich), 2.5 mg/mL carob bean galactomannan (Megazyme) and 2.5 mg/mL AGPs from *A. thaliana* PSB-D plant cell suspension cultures (Van Leene et al., 2011; Tryfona et al., 2012). Released α-D-Gal*p* and β-L-Ara*p* moieties were detected using the K-ARGA kit (Megazyme), which makes use of a galactose mutarotase and β-galactose dehydrogenase to convert L-Ara and D-Gal to their β-anomeric form and to oxidize the β-sugars to L-arabinonic acid, D-galactonic acid and NADH + H^+^. The amount of NADH formed is measured spectrophotometrically at a wavelength of 340 nm (Fukimura, 1988; Sturgeon, 1988) using a GENESYS150 UV/Vis spectrophotometer (Thermo Fisher Scientific) with 1.5 mL disposable 1 cm cuvettes (BRAND GmbH, Wertheim, Germany).

**Table 1:** Experimental setup for the different enzymatic assays.

Experiment	Initial activity screening	pH and temperature stability	Determination of kinetic parameters	Activity on di/oligosaccharides	Activity on polysaccharides
Substrate *^a^*	50 mM pNP-α-D-Gal*p* or 50 mM pNP-β-L-Ara*p* (100 µL)	50 mM pNP-α-D-Gal*p* (80 µL)	1–100 mM pNP-α-D-Gal*p* (120 µL)	50 mM melibiose monohydrate 50 mM raffinose pentahydrate 50 mM verbascose (240 µL)	2.5 mg/mL arabinogalactan from larch wood 2.5 mg/mL galactomannan 2.5 mg/mL arabinogalactan protein *O*-glycans from *A. thaliana* PSB-D (240 µL).
Assay type	Continuous	Discontinuous	Discontinuous	Discontinuous	
Control reaction	*pALiCE02* + pNP-α-D-Gal*p*	*pALiCE02* + pNP-α-D-Gal*p*	*pALiCE02* + pNP-α-D-Gal*p*	*pALiCE02* + pNP-α-D-Gal*p*	
Total reaction volume	200 µL	160 µL	240 µL	480 µL	
Buffer	50 µL 200 mM Tris-HCl pH 7.5	40 µL 200 mM Tris-HCl (pH 6, 7, 7.5, 8, 9).	60 µL 200 mM Tris-HCl (pH 8)	120 µL 200 mM Tris-HCl (pH 8)	
Enzyme	50 µL	40 µL	60 µL	120 µL	
Temperature	22°C (room temperature)	25°C, 30°C, 35°C, 40°C	25°C	25°C	
Sampling volume	Not applicable	50 µL		100 µL	
Sampling points	Every 5 min for 2 h	5 min, 1 h, 2 h	5 min, 30 min, 1 h, 2 h	5 min, 30 min, 1 h, 2 h	
Inactivation	Not applicable	50 µL 500 mM Na_2_CO_3_ (pH 11) (Sigma-Aldrich)	50 µL 500 mM Na_2_CO_3_ (pH 11 (Sigma-Aldrich)	5 min at 95°C	
Assay	Absorbance at 405 ± 10 nm			K-ARGA enzymatic/colorimetric kit (Megazyme), absorbance measurement at 340 nm.	

*^a^* concentrations correspond to initial concentrations. The final concentration equals half of the initial concentration.

#### 2.2.2 Calculation of kinetic parameters

Initial reaction velocities (v_0_) in mol·L^-1^·s^-1^ are calculated by measuring the release of pNP or NADH using the Lambert-Beer law (Equation 1).


(1) $${\text{v}}_{0}=\frac{\text{d}[\text{P}]}{\text{dt}}=\frac{\text{dA}}{\text{ϵ}\cdot \text{L}\cdot \text{dt}}$$

With: d[P]: increase of product concentration in mol·L^-1^; dt: time coordinate in s; dA: increase of absorbance at 405 ± 10 nm (pNP) or 340 nm (NADH), unitless; ϵ: molecular extinction coefficient (pNP: 18000 mol·L^-1^·cm^-1^; NADH: 6300 mol·L^-1^·cm^-1^); L: pathlength in cm.

The catalytic activity expressed as katals (1 kat = 1 mol·s^-1^) is calculated by multiplication of the initial velocity with the reaction volume as indicated in **Table 1**. We define 1 unit (U) as the release of 1 µmol product (*i.e.* pNP or NADH) in 1 minute. For the determination of K_M_ and V_max_, the Hanes-Woolf linearization method was used (Hanes, 1932). The calculated the K_M_ and V_max_ were used to construct a theoretical Michaelis-Menten plot according to Equation 2 (Michaelis and Menten, 1913). The resulting hyperbole was compared with the obtained experimental values for v_0_ and the quality of the fit was evaluated by R² values.


(2) $${\text{v}}_{0}=\frac{{\text{V}}_{\text{max}}\cdot [\text{S}]}{{\text{K}}_{\text{M}}+[\text{S}]}$$

With: [S]: substrate concentration in mol·L^-1^, V_max_: maximum reaction velocity in mol·L^-1^·s^-1^; K_M_: Michaelis constant in mol·L^-1^.

### 2.3 Protein modelling, molecular dynamics and phylogeny

#### 2.3.1 Determination of substrate-binding affinities

Modeling of OsAPSE was performed with AlphaFold (Jumper et al., 2021) (RRID: SCR_025454), while the thermodynamic quality of the model was assessed in Swiss-Model (Waterhouse et al., 2018) (RRID: SCR_018123). Molecular structures of monosaccharides (L-Ara*p*, D-Xyl*p*, D-Glc*p*, D-Gal*p*, D-GalNAc) and oligosaccharides (melibiose, raffinose) used in the docking experiments, were retrieved from PubChem^1^ (RRID: SCR_004284). Carbohydrates were docked to the GH27 domain of OsAPSE with SwissDock (Grosdidier et al., 2011; Bugnon et al., 2024) (RRID: SCR_022564), using the Attractive cavities method (Zoete et al., 2016; Röhrig et al., 2023). Binding affinities between the GH27 domain of OsAPSE and the carbohydrates were calculated. Molecular cartoons were drawn with the Chimera software (Pettersen et al., 2004) (RRID: SCR_004097).

#### 2.3.2 Molecular dynamics simulations

The number of hydrogen bonds arising between the GH27 domain of OsAPSE and different carbohydrate structures was determined for simulations of 100 ns duration (Osterne et al., 2024). For these analyses, the AlphaFold structure of OsAPSE was used, combined with a set of additional carbohydrates compared to previous simulations, downloaded from PubChem or ChemSpider^2^ (RRID: SCR_006360) (D-Gal*p*, L-Ara*p*, D-GalNAc, D-GlcNAc, pNP-D-Gal*p*, pNP-L-Ara*p*, melibiose, raffinose, stachyose, verbascose and ajugose). Carbohydrates were docked into the catalytic site of the GH27 domain using the GOLD software v2023 (RRID: SCR_000188) within the Hermes suite using standard settings (Jones et al., 1997; Verdonk et al., 2003). Docking grids of 7 Å and 12 Å, were established around the catalytic site for docking of monosaccharides and oligosaccharides respectively. CHEMPLP scores were used to evaluate the most favorable protein-carbohydrate interactions. CHARMM-GUI (RRID: SCR_025037) was used to prepare a TIP3P solution system, with neutralizing Na^+^ and Cl^-^ ions (Jorgensen et al., 1983; Jo et al., 2008; Brooks et al., 2009; Lee et al., 2020). The simulations were executed using the pmemd.cuda module of AMBER23 with the ff19SB and GLYCAM_06j force fields for the protein and carbohydrates, respectively (Kirschner et al., 2008; Tian et al., 2020; Case et al., 2023). Simulations were performed under isobaric (Monte Carlo barostat, 1 bar) and isothermal (Langevin thermostat, 300 K) conditions (Berendsen et al., 1984; Loncharich et al., 1992). Pressure and temperature were equilibrated by 500 ps in the NPT and NVT ensembles, respectively. Every simulation was run for 50 ns, collecting 5000 frames. Intermolecular hydrogen bonds were analyzed using of the Cpptraj module and visualized in Xmgrace (Roe and Cheatham, 2013).

#### 2.3.3 Phylogenetic analyses

Phylogenetic analyses of GH27 and APSE sequences from plant species (*Viridiplantae*), grasses and cereals (*Poales*) and across kingdoms were executed. GH27 sequences were obtained through the Conserved Unique Peptide Patterns (CUPP) database (**Supplementary File S3**) (Barrett et al., 2020) (RRID: SCR_026501). The CANDY tool for carbohydrate active enzyme domain analysis was employed to analyze the protein domain modularity (Windels et al., 2024). Additional phylogenetic analyses based on the GH27 domain sequences were executed using phylogeny.fr (Dereeper et al., 2008) (RRID: SCR_010266). Sequences were aligned using MUSCLE v3.8.31 (RRID: SCR_011812). Phylogenetic analysis was performed using the Maximum Likelihood method implemented in PhyML v3.1/3.0 aLRT (RRID: SCR_014629) within the phylogeny.fr pipeline. Tree reconstruction employed the WAG substitution model with empirical amino acid frequencies. Rate heterogeneity among sites was modeled using a gamma distribution and included a proportion of invariant sites. Amino acid frequences were estimated from the alignment and used in the Maximum Likelihood calculations. Support for clades was evaluated using both bootstrap analysis and approximate likelihood-ratio tests. Trees were rendered using TreeDyn v198.3 (RRID: SCR_015946) and the resulting phylograms were visualized using the interactive Tree of Life v6.0 (Letunic and Bork, 2024) (RRID: SCR_018174) and formatted using inkscape v1.3.2 (RRID: SCR_014479). Multiple sequence alignments were performed in Clustal Omega (Madeira et al., 2024) (RRID: SCR_001591) and used as input to generate a WebLogo using WebLogo3 (Crooks et al., 2004) (RRID: SCR_010236). PyMOL v2.5.4 (RRID: SCR_000305) was used for structural comparisons of 3D models, either from crystallization data (PDB) or AlphaFold models obtained via UniProt (RRID: SCR_002380) (**Supplementary File S4**). Domain coordinates were extracted from InterPro (RRID: SCR_006695). Root-mean square deviation (RMSD) values were used to assess the structural alignment quality (Shindyalov and Bourne, 1998; Kufareva and Abagyan, 2011).

#### 2.3.4 In silico prediction of biochemical protein properties

Biochemical protein properties including molecular weight, iso-electrical point, amino acid distribution, stability index and hydrophobicity index were calculated using ExPASy ProtParam (Gasteiger et al., 2003) (RRID: SCR_018087). Prediction of post-translational modifications (PTMs) was done through NetNGlyc v1.0 for *N*-glycosylation (Gupta and Brunak, 2002) (RRID: SCR_001570), NetOGlyc v4.0 for *O*-glycosylation (Steentoft et al., 2013) (RRID: SCR_009026), diANNA v1.1 for disulfide bridges (Ferre and Clote, 2005) (RRID: SCR_018529), SignalP v6.0 for signal peptides (SP) (Teufel et al., 2022) (RRID: SCR_015644), TargetP v2.0 for other transfer peptides (Almagro Armenteros et al., 2019) (RRID: SCR_019022), NucPred for the presence of Nuclear Localization Signals (NLS) (Brameier et al., 2007) (RRID: SCR_026502) and DeepLoc v2.1 for membrane association (Ødum et al., 2024) (RRID: SCR_026503).

### 2.4 Expression of OsAPSE during rice seed germination

#### 2.4.1 Cultivation of transgenic, mutant and wild type rice

Transgenic *O. sativa* subsp. Japonica cv. Kitaake lines were created by means of *Agrobacterium*-mediated transformation, including 3 overexpression *pUBI::OsAPSE* and 3 knock-out *osapse* lines. The overexpression *pUBI::OsAPSE* lines were generated using a binary vector harboring a hygromycin resistance gene, in which the *OsAPSE* coding sequence is under the control of the constitutive maize ubiquitin promoter and a nopaline synthase terminator. Mutant *osapse* lines were generated using the CRISPR-Cas9 system, with 2 guide RNAs (gRNA) directed against the coding sequence of OsAPSE: 5’-CTTGCTGAGTTTCCACCAAGAGG-3’ and 5’-CATCATCCAGAATTGATAAAGGG-3’ (*i.e.* single gene, dual target) (Jiang and Doudna, 2017).

Rice seeds were de-husked with coarse sandpaper, sterilized by incubation on a rotary wheel (10 rpm) in 70% ethanol (Chem-Lab) for 5 min followed by 45 min in 5% commercial bleach (Carrefour supermarket), washed 7–10 times with sterile water and incubated overnight in sterile water on a rotary wheel. Afterwards, rice seeds were sown on Murashige and Skoog (MS) medium (pH 5.7-5.8) with modified vitamins (Duchefa Biochemie), 3% sucrose (Chem-Lab) and 1.5% micro agar (Duchefa Biochemie). Seeds were germinated for 10 days inside a controlled Adaptis growth cabinet (Conviron, Winnipeg (MB), Canada) at 28°C using a 16/8 photoperiod with photon flux density of 310 µmol·m^-2^·s^-1^. Afterwards, rice seeds were brought to greenhouses of the Institute for Agriculture and Fishery Research (Instituut voor Landbouw en Visserijonderzoek) in Melle, Belgium (50°59’35.667” N, 3°47’4.902” O) for seed multiplication. Rice plantlets were transferred from MS medium to general potting soil in 30 cm diameter pots. The rice plants were cultivated aerobically at 25-30°C and were watered daily using a tidal irrigation system. Additional iron and ammonium were supplemented with 0.18% FeSO_4_ (Carl Roth GmbH) and 0.09% (NH_4_)_2_SO_4_ (Chem-Lab) during the first weeks of growth. After 6–8 months, rice seeds were harvested and dried at 28°C for 2 weeks prior to further usage.

#### 2.4.2 Characterization of transgenic and mutant rice plants

Wild type (WT), transgenic overexpression *pUBI::OsAPSE* and mutant *osapse* plants were grown as described above. At the age of 1 month, 3–4 cm samples of young rice leaves were collected in duplicate in sterile round-bottom safe-lock Eppendorf tubes and stored on dry ice during transportation and at -80°C until further usage. Rice material was ground using a Tissue Lyser II (Qiagen) with magnetic beads (Ø 3 mm) and prior cooling on liquid nitrogen. Afterwards, 0.1 g crushed leaf material was mixed with 1 mL DNA extraction buffer containing 2% hexadecyl-trimethyl ammonium bromide (CTAB) (Sigma-Aldrich), 0.1 M Tris-HCl, pH 7.5, 1.4 M NaCl and 2 mM Na_2_EDTA (Sigma-Aldrich) followed by extraction using a mixture of chloroform (Chem-Lab) and isoamyl-alcohol (Carl Roth GmbH) in 24:1 ratio. Total genomic DNA (gDNA) was precipitated with 100% isopropanol (Chem-Lab) and washed with mixtures of 76% ethanol + 0.2 M NaOAc pH 8 (Merck) and 76% ethanol + 10 mM NH_4_OAc pH 6 (Chem-Lab). The DNA pellet was dissolved in 50 µL sterile water and stored at -20°C.

PCR analyses using gDNA extracted from *pUBI::OsAPSE* plants, allowed to amplify a fragment of the hygromycin resistance gene using Taq DNA polymerase (VWR), 2 µL DNA with initial denaturation at 95°C for 5 min, 35 cycles (95°C for 30s, 52°C for 30s, 72°C for 30s) and final elongation at 72°C for 5 min (**Supplementary File S2**). Similarly PCR using gDNA from mutant *osapse* plants, aimed to amplify the target region for CRISPR knock-out using ALLin™ Mega HiFi Red Mastermix (highQu GmbH, Kraichtal, Germany), 2 µL DNA with initial denaturation at 95°C for 5 min, 35 cycles (95°C for 30s, 60°C for 45s, 72°C for 30s) and final elongation at 72°C for 5 min (**Supplementary File S2**). The resulting PCR amplicons were purified using the QIAquick^®^ PCR & Gel Cleanup Kit (Qiagen) and sequenced (Biosearch/LGC Genomics GmbH). Amplified sequences of WT and *osapse* plants were aligned to screen for mutations, caused by non-homologous end-joining after Cas9 endonuclease-mediated double-stranded breaks. The effect of mutations on the resulting polypeptides was assessed using AlphaFold (Jumper et al., 2021).

#### 2.4.3 OsAPSE expression during rice seed germination

WT seeds, transgenic overexpression *pUBI::OsAPSE* and mutant *osapse* seeds from the F_3_ generation were de-husked, sterilized and sown on non-selective MS medium as described above. The number of germinating and non-germinating (dead) seeds was counted at 1, 4, 7 and 11 days post imbibition (dpi), with 20 seeds per time point. Germination rates were calculated. Total seedling material, including roots, shoots and seeds from 8–10 plantlets per biological replicate were collected at 3, 7 and 10 dpi for the *pUBI::OsAPSE* and *osapse* lines, and at 1, 4, 7 and 11 dpi for WT, with minimum 3 biological replicates per sampling point. Different samples were used for RNA extraction and for germination assays. The *OsAPSE* transcript levels for WT at 4-7–11 dpi, *pUBI::OsAPSE* overexpression lines and mutant *osapse* lines at 3-7–10 dpi were correlated to the germination rates of WT, *pUBI::OsAPSE* overexpression lines and mutant *osapse* lines at 4-7–11 dpi. The germination rates at 1 dpi were excluded since *OsAPSE* transcript levels in *pUBI::OsAPSE* overexpression lines and mutant *osapse* lines were not determined at 1 dpi.

Plant material was crushed to a fine powder using a mortar, pestle and liquid nitrogen. All materials were decontaminated and rinsed between samples, using 70% ethanol and RNase AWAY (Thermo Fisher Scientific). Crushed materials were stored at -80°C until further usage. Total RNA was extracted using the Spectrum™ kit (Sigma-Aldrich), treated with RNase-free DNase I (Thermo Fisher Scientific) and RiboLock RNase inhibitor (Thermo Fisher Scientific) to remove co-extracted gDNA. Complementary DNA (cDNA) was synthesized from 500 ng RNA using Maxima Reverse Transcriptase (Thermo Fisher Scientific) according to the manufacturers’ protocol. The obtained cDNAs were diluted 5 times in ultrapure water prior to further usage, and stored at -20°C. In between operations, RNA quality and quantity were analyzed using a NanoDrop2000 spectrophotometer (Thermo Fisher Scientific). RT-PCR for quality control of the cDNA samples was executed, amplifying reference genes as controls (**Supplementary File S2**), using 2 µL cDNA and Taq DNA polymerase with initial denaturation at 95°C for 5 min, 40 cycles (95°C for 30s, 58°C for 30s, 72°C for 30s) and final elongation at 72°C for 5 min. Finally, RT-qPCR was performed with 8 µL 4x diluted cDNA, 1 µL of each primer and 10 µL iQ™ SYBR^®^ Green Supermix (Bio-Rad) in a CFX Duet Real-Time PCR System (Bio-Rad) using the following amplification protocol: 95°C for 3 min followed by 42 cycles (95°C for 15s, 60°C for 25s, 72°C for 20s). Melting curves from 65°C to 95°C were generated with 0.5°C increments and fluorescence measurements every 5 s and analyzed using the CFX Maestro v2.3 software. The generated output was analyzed in qBase+ (Hellemans et al., 2007). The list of reference genes is included in **Supplementary File S2**. Primers were designed using Primer3Plus (Rozen and Skaletsky, 1999) (RRID: SCR_003081). Primer amplification efficiency and stability (Bustin et al., 2009) were analyzed using the GeNorm algorithm in qBase+ (Vandesompele et al., 2002) (RRID: SCR_003370).

#### 2.4.4 Analysis of agronomical traits

Agronomical traits such as number of (im)mature seeds per panicle, seed setting rate, panicle mass and seed mass were determined by counting and weighing F_3_ seeds of WT, *osapse* and *pUBI::OsAPSE* plants. Panicles from 4–5 individual plants per line were used. Images of wild type, *pUBI::OsAPSE* and *osapse* panicles were taken using a Canon EOS 70D digital camera (Canon Inc., Shimomaruko (Tokyo), Japan) on a statue with fixed height.

### 2.5 Subcellular localization of OsAPSE

#### 2.5.1 Transient expression of OsAPSE-EGFP in *Nicotiana benthamiana* leaves

The *OsAPSE* coding sequence was codon-optimized for expression in *N. benthamiana* and synthetically produced through the GeneArt Gene Synthesis service (Thermo Fisher Scientific). The *OsAPSE* sequence was cloned into the Gateway™-compatible (Invitrogen, Carlsbad (CA), USA) pK7FWG2 vector for *C*-terminal fusion with the Enhanced Green Fluorescent Protein (EGFP) (Karimi et al., 2002) under control of the constitutive 35S Cauliflower Mosaic Virus promoter, following the cloning procedure as described earlier (Van Hove et al., 2011). The expression plasmid was confirmed through sequencing (Biosearch/LGC Genomics GmbH) and transformed (300–500 ng of plasmid DNA) in electrocompetent *A. tumefaciens* EHA105 cells through electroporation (2.5 kV, 25 µF, 400 Ω, time constant 5–6 ms). *Agrobacterium* cells were selected on yeast extract medium containing 5 g/L beef extract (Lab M Ltd., Heywood, United-Kingdom), 5 g/L peptone (Merck), 1 g/L yeast extract (Merck), 5 g/L sucrose, 15 g/L bacterial agar (Thermo Fisher Scientific), 200 µg/mL rifampicin (Duchefa Biochemie) and 50 µg/mL spectinomycin (Duchefa Biochemie), for 2 days at 28°C. Putatively transformed *Agrobacterium* colonies were analyzed through colony PCR using Taq DNA polymerase and gene-specific primers (**Supplementary File S2**), with 5 min initial denaturation at 95°C, 35 cycles (30s at 95°C, 30s at 50°C, 2 min at 72°C) and 5 min final elongation at 72°C.

Recombinant *Agrobacterium* cells were cultured overnight at 28°C (180 rpm) in selective yeast extract broth. Similarly, *A. tumefaciens* GV3105 cells harboring the empty vector *pK7FWG2* plasmid as free-EGFP positive control was cultured. All *Agrobacterium* cells were cultured until OD_600_ = 0.75 – 0.85. Thereafter, cells were washed using infiltration medium (pH 5.6) containing 10 mM 2-(N-morpholino)-ethanesulfonic acid (Carl Roth GmbH), 2 mM Na_2_HPO_4_ (VWR), 0.5% glucose (Carl Roth GmbH), till OD_600_ = 0.4 and prepared for infiltration by adding acetosyringone (Sigma Aldrich) to a final concentration of 100 µM.

The abaxial side of 3–5 weeks old *N. benthamiana* leaves (Bally et al., 2018) was transiently transformed with a suspension of *Agrobacterium* cells harboring either the *pK7FWG2::OsAPSE* plasmid or the empty vector *pK7FWG2* control (Sparkes et al., 2006). The *Agrobacterium* suspensions were administered using 2 mL syringes without a needle. Thereafter, the infiltrated area was highlighted with thin marker and the plants were incubated at 28°C for 2–3 days prior to microscopic analysis. Nuclear colocalization was visualized with 4’,6-diamidino-2-phenylindole (DAPI) (Thermo Fisher Scientific), whereby a working solution at final concentration of 10 µg/mL was infiltrated 30 minutes prior to microscopy analysis.

#### 2.5.2 Confocal fluorescence microscopy and image acquisition

A Nikon A1R confocal laser scanning microscope mounted on a Nikon Ti-E inverted epifluorescence body (Nikon instruments, Shinjuku, Japan) was used to capture confocal images. EGFP was excited at a wavelength of 488 nm using an argon laser and detected using an emission filter (515–530 nm). Microscopy analysis and image acquisition with the Fiji ImageJ software (Schindelin et al., 2012) were executed as described earlier (Dubiel et al., 2020).

#### 2.5.3 In silico prediction of subcellular localization of OsAPSE

Subcellular localization was also predicted *in silico* using online webservers including MultiLoc2 (Blum et al., 2009) (RRID: SCR_003151), Plant-mPLoc (Chou and Shen, 2010) (RRID: SCR_023014), CELLO v2.5 (Yu et al., 2014) (RRID: SCR_011968), DeepLoc v1/v2.1 (Almagro Armenteros et al., 2019; Ødum et al., 2024) and MuLocDeep (Jiang et al., 2023) (RRID: SCR_026504).

### 2.6 Statistical analyses and visualizations

Statistical analyses were performed using SPSS v29.0 (RRID: SCR_002865). Throughout this study, significance levels at p < 0.05 were enforced. Comparison of means with *ν* degrees of freedom (df) was executed using the Student’s T-test for comparison between 2 samples or one-way ANOVA for comparison between >2 samples. Prior analysis of normality using the Shapiro-Wilk test and homogeneity of variance using the Levene’s test was executed when applicable. In case the normality criterium was violated, the non-parametric Mann-Whitney U test or pairwise Kruskal-Wallis test were performed. The Welch test was executed when the homoskedasticity criterium was violated. Effect sizes for comparison of means was assessed based on the reported η² value, explaining a proportion of the observed variance per dataset. For the germination assays, a generalized linear model (GLM) was fitted with a binomial distribution and logit link function to model the probability of seed germination as a function of transgenic line, time point and their interaction. For each time point, differences in germination rates between transgenic lines, knock-out lines and WT were assessed using separate binomial logistic regression models, with WT as the reference. Bar charts were generated by means of Microsoft Excel (RRID: SCR_016137) and reformatted in Inkscape v1.3.2. Diagrams were generated in BioRender (RRID: SCR_018361).

## 3 Results and discussion

### 3.1 Phylogeny of GH27 sequences from grasses and cereals

Most of the GH27 sequences found in the CUPP database belong to the taxonomical division of *Bacteria* (60.0%), followed by *Fungi* (24.1%), *Metazoa* (7.4%) and *Viridiplantae* (7.3%) (**Supplementary File S3.1**). A total of 140 GH27 sequences from grasses and cereals (*Poales*) were retrieved. After removal of 38 duplicate sequences or gene fragments (**Supplementary File S3.2**), the CANDY tool was employed for modularity analysis (Windels et al., 2024). Several recurring InterPro domains were found across the GH27 sequences (**Supplementary File S3.3**). All sequences were attributed with the GH27-related IPR002241 and GH superfamily-related IPR017853 identifiers. The IPR041233 and IPR013780 identifiers both refer to the same domain, although the IPR041233 identifier is used specifically for MELs and the IPR013780 identifier applies to GH-all-beta domains in general. Almost every retrieved GH27 sequence contained the *C*-terminal GH-all-beta domain, which is a common terminal domain for a number of GH families, including GH5, GH13 and GH42. Structurally, this *C*-terminal domain resembles a Greek key β-sandwich (Hutchinson and Thornton, 1993). Furthermore, several sequences were provided with the IPR035992 identifier describing ricin-B-like lectin domains (Van Holle et al., 2017; De Coninck et al., 2024b).

**Figure 1:**
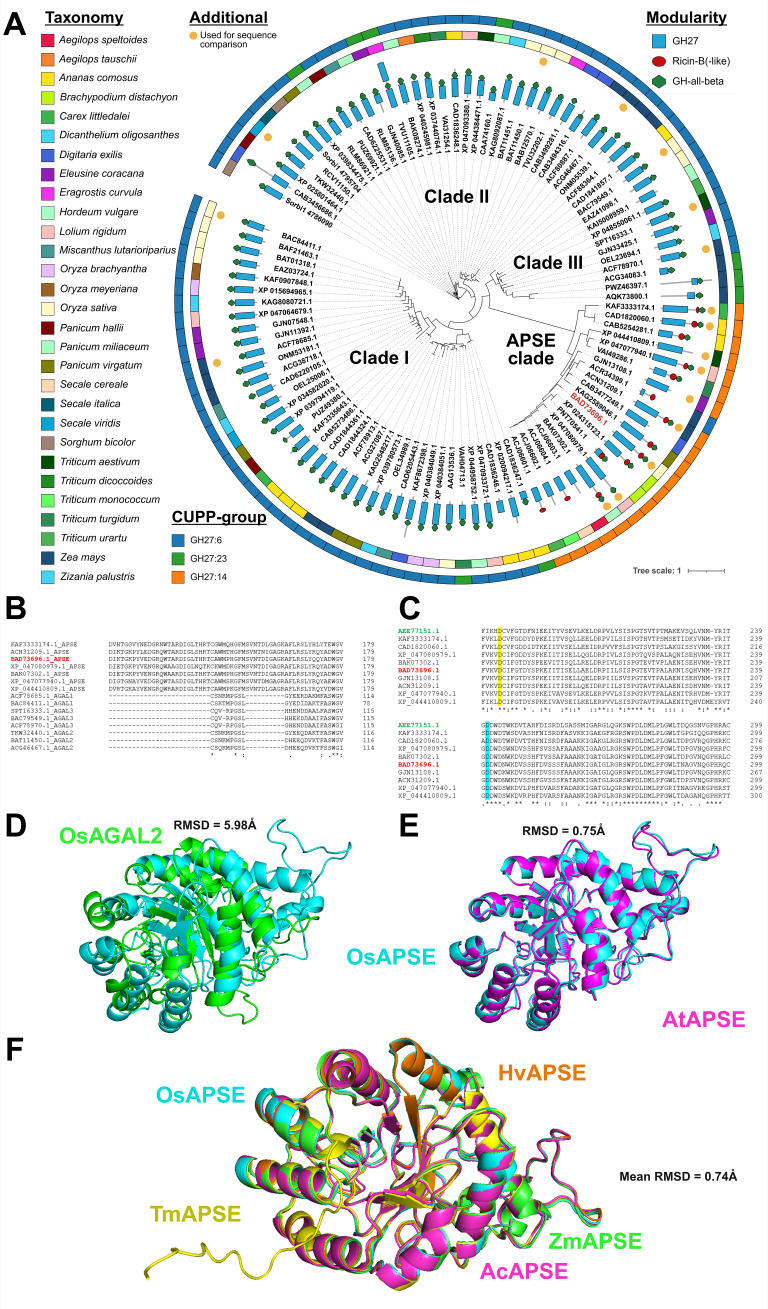
Phylogenetic and structural analysis of GH27 domain sequences from *Poales*. Modularity of the full-length GH27 sequences, represented by their corresponding GenBank IDs, is displayed by blue rectangles (GH27 domain), red circles (Ricin-B(-like) domain) or green hexagons (GH-all-beta domain). The outer strips represent taxonomy and CUPP-group and are colored as indicated in the legend. OsAPSE (GenBank ID: BAD73696.1) is highlighted in red. GH27 domain sequences used in the sequence alignment are highlighted with an orange circle **(A)**. Partial alignment of a selection of GH27 domains from the APSE clade and defined clades I, II and III denoted as AGAL1, AGAL2 and AGAL3 **(B)**. Partial alignment of GH27 domains from the APSE clade with the characterized APSE domain from *Arabidopsis thaliana* (highlighted in green) and OsAPSE (highlighted in red), illustrating the conservation of the catalytic residues. The catalytic nucleophile is highlighted in yellow and the catalytic acid/base in cyan **(C)**. Explanation of symbols: gap (**-**), conserved residue (.), highly conserved residue (): identical residue (*). The OsAPSE sequence is highlighted in red. Structural alignment of OsAPSE (cyan) with OsAGAL2 (green; GenBank ID: BAC79549.1) **(D)**. Structural alignment of OsAPSE (cyan) with AtAPSE (magenta) **(E)**. Multiple structural alignments of OsAPSE (cyan) with other APSE clade members of *Hordeum vulgare* (orange; GenBank ID: BAK07302.1*), Zea mays* (green; GenBank ID: ACN31209.1), *Ananas comosus* (magenta; GenBank ID: CAD1820060.1) and *Triticum monococcum* (yellow; GenBank ID: ACJ06602.1) **(F)**. RMSD values were calculated using the cealign algorithm.

Phylogenetic analyses based on the full-length sequences of GH27 enzymes were executed and enabled the identification of distinct clades (**Figure 1A**). The gamma shape parameter was estimated at α = 4.44 indicating moderate rate variation across sites. Approximately 9.4% of the sites were inferred to be invariant. Intriguingly, ricin-B-like domains have only been identified in a subpopulation of the GH27 sequences from *Poales*. As shown in the phylogram (**Figure 1A**), ricin-B-like domains are present only in the so-called “APSE clade”, which is distant from other GH27 sequences without a ricin-B-like domain. This is also observed when GH27 sequences across kingdoms are studied (**Supplementary File S3.1**). Members within the APSE clade in *Poales* species show high sequence similarity (median >70%; **Supplementary File S3.4**) towards the characterized APSE from *Arabidopsis thaliana* (Imaizumi et al., 2017) and have highly conserved amino acid sequences, judging from weblogos (**Supplementary File S3.5**) and multiple sequence alignments (**Supplementary File S3.6**).

The presence of a ricin-B-like domain is a distinctive trait to categorize the GH27 family. Almost all members from the APSE clade have been designated as CUPP group GH27:14, while all other GH27 sequences from *Poales* are found in CUPP groups GH27:6 and GH27:23 (**Figure 1A**). Belonging to different CUPP groups indicates that unique peptide patterns exist amongst APSE proteins and other GH27 proteins. These peptide patterns are highly conserved across species and are indicative for a unique protein structure and biological function (Barrett et al., 2020). Next to the APSE clade, three other clades have been identified, numbered with Greek letters I, II and III, and coincide mostly with CUPP groups GH27:6 and GH27:23. The apparent separation of the APSE clade from the other clades is not only attributed to the ricin-B(-like) domain, but also to the GH27 domain itself. When phylogenetic analysis is executed only on the GH27 domains of *Poales*, the APSE clade remains phylogenetically isolated from the other GH27 sequences, but the formation of clades I, II and III is now lost (**Supplementary File S3.7**), emphasizing the highly dissimilar nature of the APSE GH27 domain compared to other GH27 sequences.

Striking differences are apparent between GH27 domains from the APSE clade compared to GH27 domains from clades I, II and III. Although GH27 domains from clades I, II and III are mutually remarkably similar, they contain several large gaps compared to GH27 domains from the APSE clade (**Figure 1B**, **Supplementary File S3.8**). Despite the important differences between APSE and clade I, II, III GH27 sequences, the catalytic residues are conserved, which is a known characteristic of the GH27 family (**Figure 1C**, **Supplementary File S3.8**) (Hart et al., 2000; Ly et al., 2000; Garman et al., 2002; Fujimoto et al., 2003; Guce et al., 2010).

The dissimilarities between APSE members and other GH27 sequences are also present at the structural level. Structural comparison between OsAPSE and a selection of GH27 AGAL AlphaFold or PDB models from *Z. mays*, *T. aestivum*, *O. sativa* and *S. viridis* (**Supplementary File S4.1**) yields bad structural alignments with mean RMSD = 5.97 ± 0.06 Å. Surprisingly, also the alignment between OsAPSE and the other OsAGALs from rice yields bad structural alignments with mean RMSD = 4.57 ± 1.54 Å (**Figure 1D**) (**Supplementary File S4.2**). Likewise, poor RMSD values (5.37 ± 1.15 Å) are obtained when the OsAPSE GH27 domain was compared to the GH27 domains of characterized GH27 proteins from other kingdoms (**Supplementary File S4.1**). In contrast, structural comparison between OsAPSE and APSE sequences from the characterized *A. thaliana* AtAPSE (**Figure 1E**) and *H. vulgare*, *A. comosus*, *Z. mays*, *Triticum monococcum* (**Figure 1F**) yields close structural alignments (0.74 ± 0.51 Å) (**Supplementary File S4.2**).

### 3.2 Modelling and molecular dynamics of OsAPSE

#### 3.2.1 Modelling of OsAPSE

**Figure 2:**
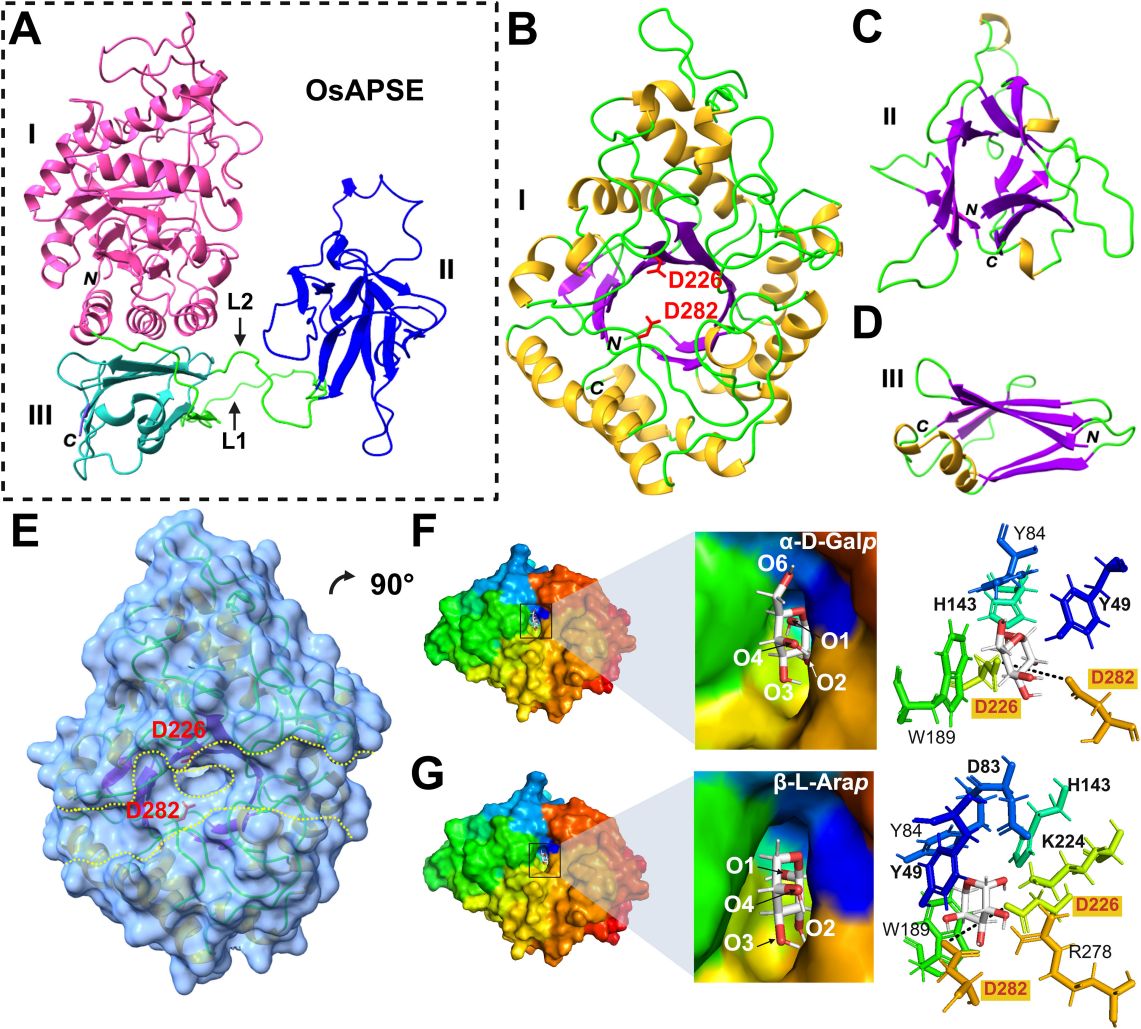
Structure of OsAPSE and interaction with monosaccharide substrates. OsAPSE is a 3-domain protein **(A)** comprising of a GH27 domain **(B)** with a catalytic cleft **(E)**. The other subdomains are the ricin-B-like domain **(C)** and the GH-all-β domain **(D)**. The Roman numbers I, II and III enumerate the subdomains of OsAPSE. L1 and L2 are two linkers connecting the GH27 domain with the ricin-B-like domain and the ricin-B-like domain and the GH-all-β domain respectively. The capital italic letters *N* and C indicate the *N* and *C*-terminal ends of OsAPSE and its subdomains. OsAPSE is shown without its native signal peptide. The catalytic residues D226 and D282 are indicated in red in sub-figures **(B, E)**. In sub-figure **(E)**, the protein surface is represented with a transparency of 40% to show localization of α-helices, β-strands and catalytic residues D226 and D282 in the vicinity of the entry of the active site (encircled yellow dashed line) within the catalytic groove of the domain (parallel yellow dashed lines). The monosaccharide substrates α-D-Gal*p* **(F)** and β-L-Ara*p* **(G)** were docked in the catalytic site of the GH27 domain. The black dashed line is indicative for the distance between the two catalytic residues D226 and D282 and measures 6.8 Å.

The OsAPSE protein is composed of 3 domains: an *N*-terminal GH27 catalytic domain, linked by a long loop (L1) to a β-trefoil ricin-B-like domain with a putative CRD, and a *C*-terminal GH-all-β domain of unknown function linked by a shorter loop (L2) to the ricin B(-like) domain (**Figure 2A**). Modeling of the structure of OsAPSE resulted in a low Qualitative Model Energy Analysis (QMEAN) value of 0.54. The low QMEAN value mostly depends on the occurrence of extended loops L1 and L2 connecting the ricin-B-like domain with the *N*-terminal GH27 and *C*-terminal GH-all-beta domain, respectively (Benkert et al., 2011; Studer et al., 2020). Modelling of extended loops typically gives conformations of poorly reliable geometric and thermodynamic quality (Fiser et al., 2000), in our case accounting for 1.85% and 1.78% of the Ramachandran and rotamer outliers, respectively. In addition, the QMEAN value of the individual GH27 domain was determined, yielding a QMEAN value of 0.69. The reported QMEAN value is considerably higher compared to the value of the complete OsAPSE protein, due to the presence of rigid α-helices and β-sheets in the TIM barrel of the GH27 domain. However, the value was somewhat lowered due to the presence of flexible loops connecting the α-helices and β-sheets.

The catalytic domain exhibits a canonical TIM (α_8_β_8_) barrel structure, made of a central crown of 8 β-strands, linked by short loops to a peripheral crown of 8 α-helices (**Figure 2B**), which is a common structure in enzymes (Vega et al., 2003). The catalytic cleft occupies the center of the TIM barrel and two aspartic acid residues (D226 and D282) located at the center of the catalytic cleft, form the active site of the enzyme (**Figure 2B**). These catalytic residues are highly conserved (**Figure 2E**) within the GH27 family (Zhu et al., 1995; Hart et al., 2000; Ly et al., 2000; Garman et al., 2002; Fujimoto et al., 2003; Guce et al., 2010; Okazawa et al., 2015; Kytidou et al., 2018). The ricin-B(-like) domain comprises 3 bundles of β-sheets organized in a typical β-trefoil lectin structure with putative carbohydrate-binding activity (**Figure 2C**) (Hazes, 1996; Steeves et al., 1999). The short *C*-terminal all-beta domain is made of 2 anti-parallel β-sheets forming a β-sandwich (**Figure 2D**). The function of all-beta domains is multifarious: they provide structural stability and assist in protein folding by acting as a nucleation site (Boissinot et al., 1997; Kemplen et al., 2015).

#### 3.2.2 Docking of substrates and molecular dynamics

Because of its close structural similarity to AtAPSE, it was expected that OsAPSE will display AGAL and ARAP activity against carbohydrate structures from the cell wall. Therefore, it was hypothesized that the main substrates for OsAPSE would be molecules with α-D-Gal*p* and/or β-L-Ara*p* side chains. Docking experiments performed with the GH27 domain (**Supplementary File S5.1**) showed that all the assayed mono- and oligosaccharides bind to the catalytic pocket via a network of hydrogen bonds with the catalytic residues D226 and D282 and surrounding hydrophilic residues (N47, D83, H143, K224, S255, S257, R278, D318, D320, M321), although these residues varied in number and type depending on the ligand (**Supplementary File S5.1**). The substrates α-D-Gal*p* and β-L-Ara*p* were docked in stable chair conformation. For α-D-Gal*p* (**Figure 2F**), O1/2, and O3/4are predicted to make contact with the catalytic residues D226 and D282, respectively. In addition, stacking interactions between aromatic residues Y49 (O4/6), Y84 and W189 located around the catalytic pocket, and the pyranose ring of the saccharides complete and reinforce the interaction with the GH27 domain (Asensio et al., 2013; Spiwok, 2017). Similar stacking residues were observed in other GH27 enzymes from fungi (Brumer et al., 1999), chicken (Garman et al., 2002), human (Garman and Garboczi, 2004; Guce et al., 2010), rice (Fujimoto et al., 2003) and tobacco (Kytidou et al., 2018). It was expected that more or less the same residues would be involved in substrate binding to α-D-Gal*p* and β-L-Ara*p* (Ichinose et al., 2009). However, despite β-L-Ara*p* being a smaller molecule, the *in silico* docking yielded two additional residues, D83 (O1) and K224 (O2), to be involved in substrate binding next to Y49 (O4), H143 (O1), D226 (O1) and D282 (O2/3) (**Figure 2G**). These results showcase the ability of the OsAPSE GH27 domain to accommodate substrates with terminal α-D-Gal*p* and β-L-Ara*p* residues.

GH27 enzymes, including OsAPSE, adhere to the classical Koshland double-displacement retaining mechanism, characterized by two consecutive displacement steps resulting in the retention of the anomeric configuration of the released sugar (Sinnott, 1990). In OsAPSE, D282 functions as the general acid, while D226 serves as the catalytic nucleophile. Upon substrate binding, D282 protonates the aglycone, facilitating glycosidic bond cleavage and generating an oxocarbenium ion-like transition state. Subsequently, the carboxylate group of D226 attacks the C1 atom of the sugar moiety, forming a covalent galactosyl/arabinosyl-OsAPSE intermediate (Vocadlo et al., 2001). In the second step, D282 deprotonates a water molecule, activating it for nucleophilic attack on C1, leading to a second oxocarbenium ion-like intermediate. This results in the cleavage of the catalytic bond between D226 and the galactosyl/arabinosyl group, releasing free galactose or arabinose, and restoring the enzyme to its initial state.

All saccharides displayed energetically favorable interactions (ΔG < 0), within the same range (K_D_ = 8-59 µM), (**Supplementary File S5.1**), even with carbohydrates for which no particular interaction was expected, such as D-Glc*p* and D-Xyl*p*. The binding affinity for D-Glc*p* is lower compared to other saccharides. The limited differences in binding affinity between the assayed carbohydrates is probably due to the strong structural resemblance between D-Gal*p* and D-Glc*p*, which are only differing in their configuration at C4 (Homolak et al., 2024), and similarly, L-Ara*p* and D-Xyl*p* are only different at the C4 anomeric configuration (Guo et al., 2025). However, it should be emphasized that the possibility to dock alternative ligands into the catalytic site does not necessarily mean that OsAPSE will cleave off these moieties. The structural flexibility of carbohydrates during molecular dynamics simulations is often exaggerated and may distort genuine protein-carbohydrate interactions (Boonstra et al., 2016), hence experimental validation is always required (Lerbret et al., 2007). Docking of carbohydrates to the ricin-B-like domain was outside the scope of this study.

Additional saccharides with varying degree of polymerization (DP) (*i.e.* stachyose, verbascose, ajugose, GalNAc, pNP-α-D-Gal*p* and pNP-β-L-Ara*p*) were docked in the catalytic pocket of the GH27 domain of OsAPSE, within a docking grid of 7Å and the number of hydrogen bonds during molecular dynamics simulations was determined (**Supplementary File S5.2**). Most stable interactions were observed for the smaller substrates L-Ara*p*, D-GalNAc, pNP- α-D-Gal*p* and D-Gal*p*, while the interactions were least stable for verbascose (DP = 5) and ajugose (DP = 6). It should be emphasized that the results should be interpreted with reservation, as ligand docking and molecular dynamics simulations were performed with modelled protein structures.

### 3.3 Production and activity of the recombinant GH27 domain of OsAPSE

Several attempts have been undertaken to produce OsAPSE or its GH27 domain recombinantly in multiple prokaryotic and eukaryotic hosts and strains, under a wide range of experimental conditions and construct designs, but these assays were mostly unsuccessful due to protein insolubility. The lack of soluble recombinant proteins is widely recognized as a main bottleneck in proteomics research (Bhatwa et al., 2021; Beygmoradi et al., 2023). CFPS platforms derived from cell lysates can be considered when cell-based strategies are inadequate (White et al., 2013). Although, prokaryotic CFPS systems are often preferred due to the low production costs, high productivity and scalability, they may not be the platform of choice for eukaryotic proteins due to the absence of appropriate PTMs and chaperones, which impact protein folding, structure and activity (Harbers, 2014; Zemella et al., 2015). About 10 years ago a CFPS based on tobacco BY-2 lysates was developed, facilitating oxidative folding, PTMs and assembly of multidomain enzymes/antibodies (Buntru et al., 2014, 2015). The performance of this platform was already demonstrated by successfully producing an array of eukaryotic glycoproteins with disulfide bridges and proper *N*-glycosylation (Buntru et al., 2022).

#### 3.3.1 Cell-free production of the GH27 domain of OsAPSE and screening for GH27 activity

**Figure 3:**
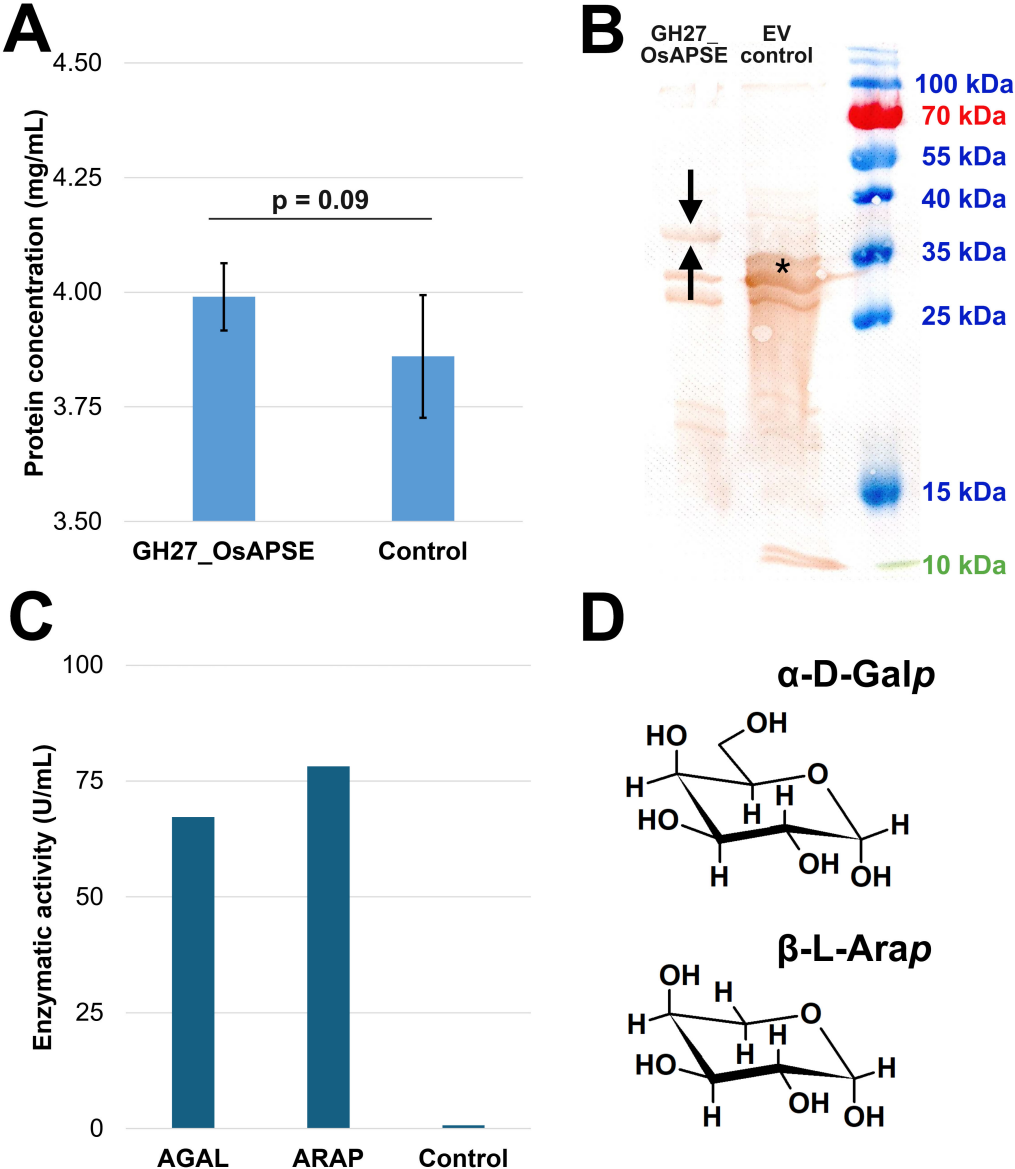
Quantification, visualization and screening for GH27 activities. Proteins were quantified using the Bradford assay. Error bars represent standard deviations based on four independent biological replicates **(A)**. Western blot with DAB detection using anti-His_6_ antibodies. The arrows indicate the protein of interest (*i.e.* GH27_OsAPSE) while the asterisk indicates the yellow fluorescent protein, present in the control reaction as reporter **(B)**. AGAL and ARAP activity expressed in U/mL, measured in a continuous assay. The control is the CFPS reaction with empty vector *pALiCE02* incubated at 25°C at pH 7.5 **(C)**. Structural comparison between α-D-Galp and β-L-Arap. Structural images were drawn using ChemSketch **(D)**. Abbreviations: EV (empty vector).

The GH27 domain of OsAPSE consists of 352 amino acids and has a predicted molecular weight of 39.9 kDa and pI = 5.99. Furthermore, the protein is predicted to be stable (instability index = 34.79) and moderately hydrophilic (Grand Average of Hydropathicity index = -0.365). Protein concentrations were estimated (**Figure 3A**) using BSA as reference protein (**Supplementary File S6**). GH27_OsAPSE was detected after Western blot analysis (**Figure 3B**). A distinct and unique protein band can be observed for the reaction with *pALiCE02::GH27_OsAPSE*. The protein polypeptide appears somewhat smaller (< 5 kDa) compared to the predicted size, but is not attributed to protease activity (Buntru et al., 2014). Deviating protein sizes are sometimes observed and are attributed to their charge distribution and more compact protein folding (Rath et al., 2009; Shi et al., 2012). The *pALiCE02* plasmid used in the control reactions contains a His_6_-tagged yellow fluorescent protein (YFP) reporter sequence, yielding a polypeptide with estimated size of 33 kDa (**Figure 3B**). The YFP sequence is removed from the *pALiCE02::GH27_OsAPSE* construct during the cloning process (**Supplementary File S7**).

A continuous enzymatic assay detected both AGAL and ARAP activity (**Figure 3C**) in the CFPS protein fractions (**Supplementary File S8.1**). This is not surprising since α-D-Gal*p* and β-L-Ara*p* are structurally very similar (**Figure 3D**) (Kotake et al., 2016), and both fit in the active site of the GH27_OsAPSE domain (**Figure 2**). OsAPSE shows high sequence identity (65%) (**Supplementary File S3.4**) and structural similarity (RMSD = 0.75Å) towards the characterized AtAPSE (**Figure 1E**), although the latter mainly demonstrated ARAP activity. However, it has been reported that AGALs may display both ARAP and AGAL activity (Imaizumi et al., 2017).

Absorbance measurements were used to calculate reaction velocities and activities (Equation 1). The reactions with *pALiCE02::GH27_OsAPSE* yielded an enzymatic AGAL and ARAP activity of 67.2 U/mL and 78.2 U/mL, respectively, whereas the control reaction with *pALiCE02* only released negligible pNP moieties (**Figure 3C**), most probably due to spontaneous degradation over the course of the enzymatic assay. Background activity originating from deglycosylases should not be present, as it was already demonstrated before that the ALiCE CFPS system gives rise to intact *N*-glycans (Buntru et al., 2022).

#### 3.3.2 Determination of temperature/pH optima and enzyme kinetics

**Figure 4:**
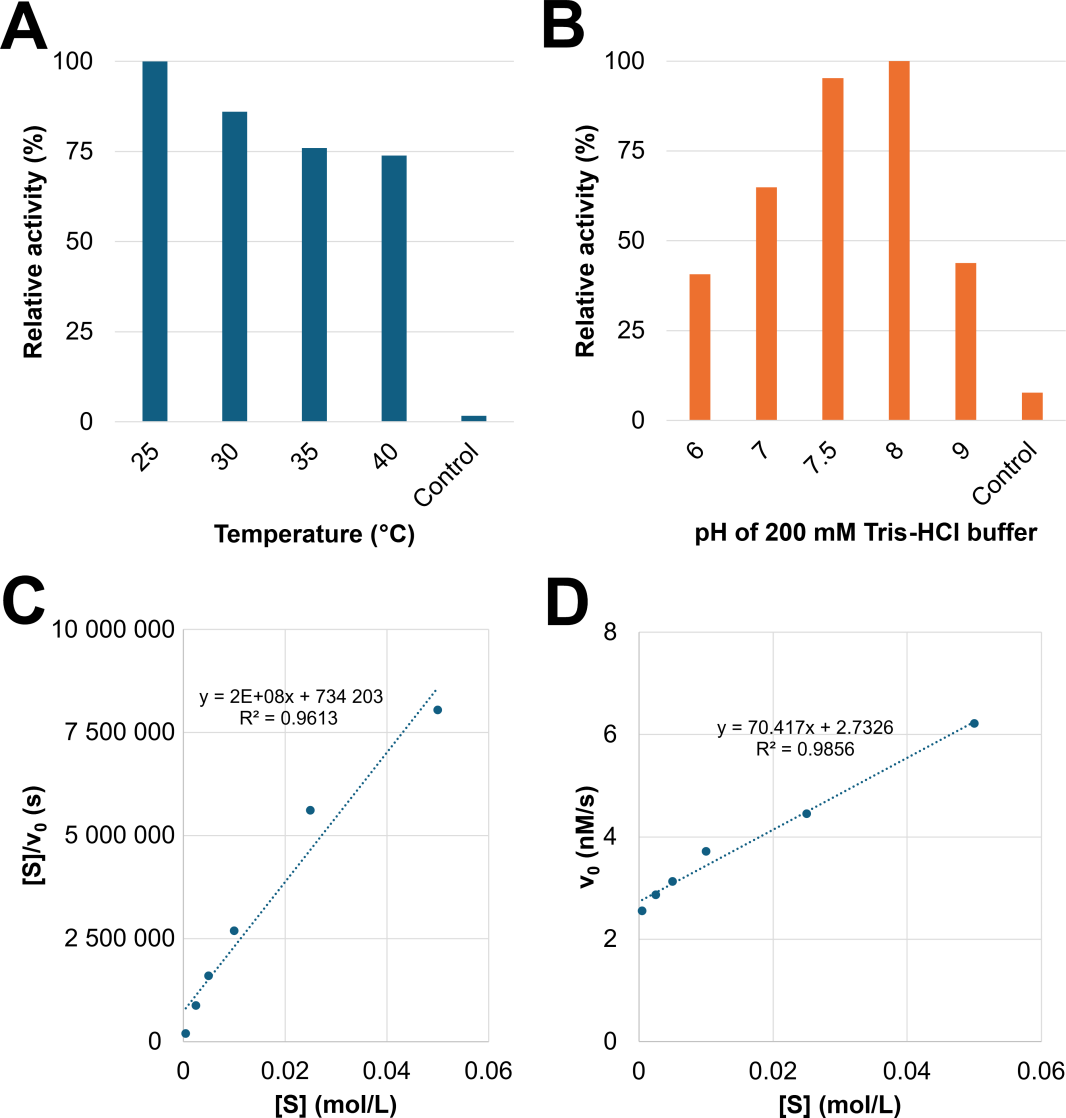
Determination of the temperature/pH optima and kinetic parameters K_M_ and V_max_. Comparison of relative activity at different temperatures **(A)**. Comparison of relative activity at different pH values **(B)**. The control is the CFPS reaction with empty vector *pALiCE02* incubated at 25°C at pH 7.5. Hanes-Woolf linearization was applied to determine K_M_ and V_max_. The slope represents V_max_^-1^, the K_M_ value is obtained by multiplying V_max_ and the value of the y-intercept. The obtained Hanes-Woolf curve is considered linear according to the R² value **(C)**. Linear relationship between v_0_ and [S] indicates that the performed experiment was conducted under substrate-saturated conditions at which v_0_ = V_max_ **(D)**.

Discontinuous enzymatic assays with pNP-α-D-Gal*p* revealed the temperature optimum of GH27_OsAPSE at 25°C (**Figure 4A**) and pH optimum at pH = 8 (**Figure 4B**) (**Supplementary File S8.2**). The relative activity decreases with increasing temperature. At temperatures around 50°C, the reaction mixtures turned opaque, due to protein denaturation and precipitation. However, the relative activity at various pH conditions shows a typical bell-shaped curve, with >95% of the activity retained between pH 7.5–8 and sharp decline when deviating from the optimum.

GH27 enzymes from plant origin are often categorized based on their pH optimum (**Supplementary File S8.5**), as there are acidic and alkaline AGALs present in plants. Plant AGALs usually display pH optima around 4.5-8.5 and temperature optima around 30-40°C although there are also AGALs with somewhat extreme optima, for instance in fava bean (pH_opt_ = 2.5) (Dey and Pridham, 1972) and maize and melon (pH_opt_ = 8.5) (Gao and Schaffer, 1999; Zhao et al., 2006). However, the pH_opt_ of OsAPSE (7.5-8) is not in accordance with its supposed biological environment, being the apoplast with typical apoplastic pH values (pH_apo_) between 5.5-6, although pH_apo_ values as low as 3.5 and as high as 8.5 have been reported before (Yu et al., 2000). The pH_apo_ in rice tissues has not been reported yet but is likely to be within the same range as closely related organisms such as barley leaves (pH_apo_ = 5.6-6.6) and maize coleoptiles (pH_apo_ = 5.7-6.0). Several cell wall-active enzymes display pH_opt_ values that differ from their surrounding physiological pH. For instance, expansins typically have a pH_opt_ = 4, which is far below the acidic pH_apo_ of 5.5-6 (Sampedro and Cosgrove, 2005). In addition, pectin methylesterases from *Arabidopsis* and citrus often have an pH_opt_ = 7–8 despite residing in generally acidic cell wall environments (Xu et al., 2022; Hocq et al., 2023). It is suggested that such pH optimum discrepancies imply enzyme dormancy until pH shifts occur. In this way, the enzyme activity is inhibited and becomes active only upon cell wall acidification or alkalinization. This built-in pH discrepancy is thought to enable rapid regulation of wall-loosening by the cell but also prevents excessive enzyme action until local conditions are adequate. Alternatively, pH discrepancies may also arise from artefacts of recombinant expression (altered folding, missing processing). An upward shift of the optimal pH due to the changed electrostatic environment of the catalytic site has been reported before (Montor-Antonio et al., 2017; Hofer et al., 2020). It is, however, difficult to predict whether or not OsAPSE was produced with an aberrant catalytical site.

AGALs with a T_opt_ = 60-65°C were reported in papaya (Soh et al., 2006) and sugarcane (Chinen et al., 1981). Stability towards pH is usually ± 2–3 pH values around the optimum. Likewise, the Q_10_ temperature coefficient for most plant enzymes is typically 2-4, meaning that the reaction rate decreases 2–4 fold with a 10°C temperature increase (Bernacchi et al., 2002; Elias et al., 2014).

Kinetic parameters including the K_M_ value and V_max_ were determined (**Supplementary File S8.3**). The absorbance did not further increase after 1 hour of incubation. The Hanes-Woolf linearization calculations were performed with the data point between substrate concentrations of 0.5–50 mM and yielded a linear relationship (R² = 0.9895) (**Figure 4C**). It was calculated that K_M_ = 0.67 mM and V_max_ = 3.9 nM·s^-1^. The initial fit to the hyperbolical Michaelis-Menten plot, according to Equation 2, yielded a poor R² 0.237 (n = 6 data points) or R² = 0.399 (n = 4 data points).


$${\text{v}}_{0}=\frac{{\text{V}}_{\text{max}}\cdot [\text{S}]}{{\text{K}}_{\text{M}}+[\text{S}]}=\frac{3.9\;\cdot 10^{-9}\;\cdot [\text{S}]}{0.67\cdot 10^{-3}+[\text{S}]}$$

It is obvious that the relationship between v_0_ and [S] is linear (R² = 0.9858) (**Figure 4D**). This is not surprising, since the experiment made use of a range of substrate concentrations between 0.5–50 mM, which are far above the calculated K_M_, under substrate-saturated conditions (v_0_ = V_max_). The value for V_max_ mainly depends on the enzyme concentration. The K_M_ value, on the other hand, is an intrinsic enzyme parameter, independent of the enzyme concentration. The observed K_M_ is in line with values reported in scientific literature (**Supplementary File S8.5**) for experiments with comparable substrates and enzymes. For instance, an AGAL from rice was produced recombinantly in *P. pastoris* and yielded a K_M_ = 0.47 mM for pNP-α-D-Gal*p* as substrate, which is only slightly lower compared to the K_M_ value in this study (Chien et al., 2008). Furthermore, K_M_ values are correlated with the complexity of the used substrate (**Supplementary File S8.5**). Larger substrates (*f.i.* RFOs), typically yield higher K_M_ values, *i.e.* lower enzyme-substrate affinity. We did not determine the K_M_ and V_max_ for pNP-β-L-Ara*p* as substrate due to limited availability of the used CFPS system and the very high probability of achieving similar values. It would not be unreasonable to assume that the K_M_ value for pNP-β-L-Ara*p* would be in the same order of magnitude, since we demonstrated that GH27_OsAPSE displays similar AGAL and ARAP activity at identical concentrations of substrate and protein (**Figure 3C**).

#### 3.3.3 Activity on natural substrates

**Figure 5:**
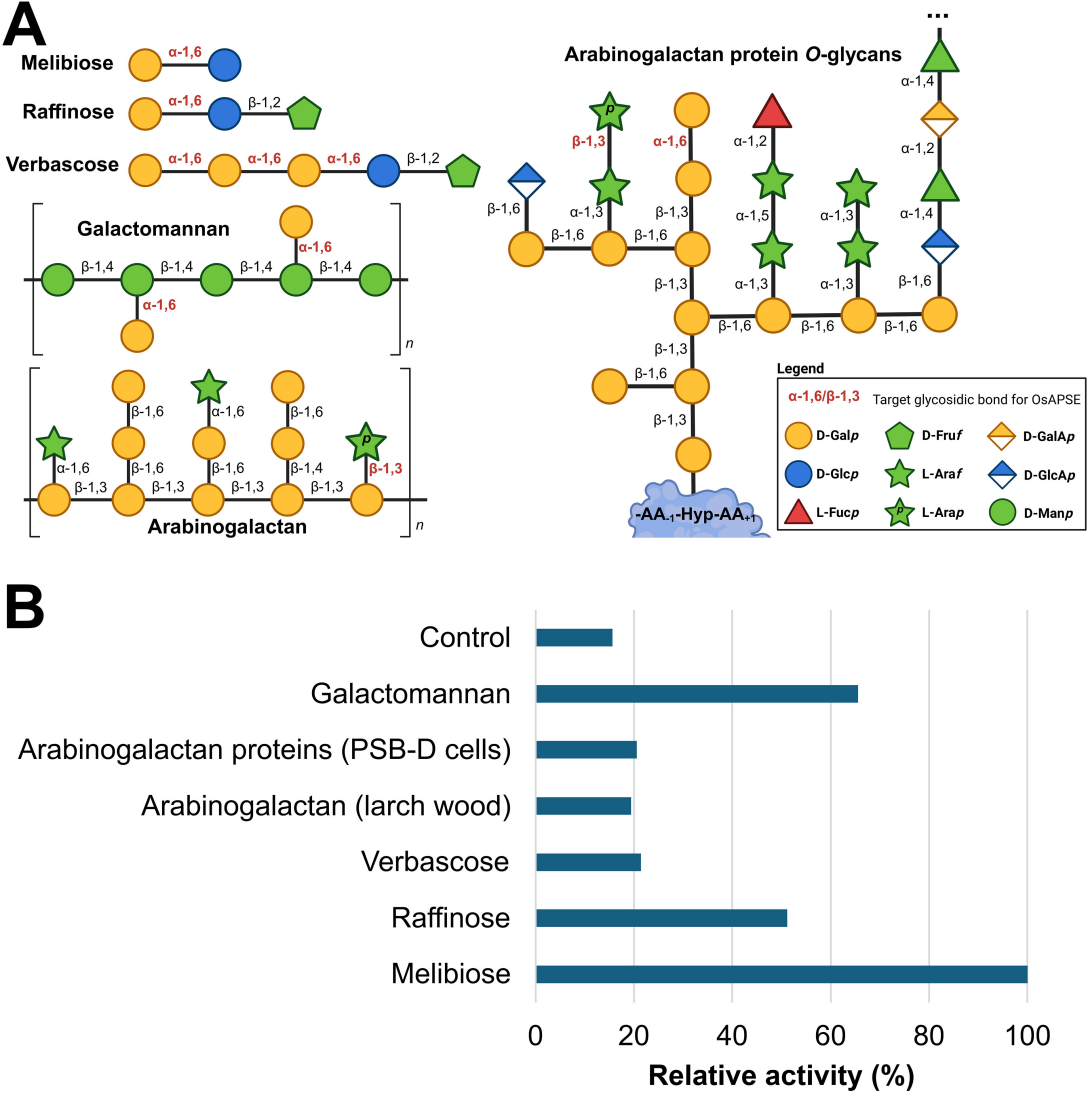
Enzymatic activity of GH27_OsAPSE on natural substrates. Structure of the considered natural substrates. The structures for galactomannan, arabinogalactan and arabinogalactan protein *O*-glycans are average structures based on theoretical models (Knoch et al., 2014; Seifert, 2020; Strasser et al., 2021; Voiniciuc, 2022; Tan et al., 2023) **(A)**. Bar chart representing relative activities of GH27_OsAPSE on natural substrates. The control is the CFPS reaction with empty vector *pALiCE02* incubated at 25°C at pH 7.5 **(B)**.

A wide range of putative substrates decorated with α-D-Gal*p* and/or β-L-Ara*p* side chains were submitted to enzymatic hydrolysis by the GH27 domain of OsAPSE (**Figure 5A**). Highest activities were observed for the disaccharide melibiose, the trisaccharide raffinose and the polysaccharide galactomannan (**Supplementary File S8.4**). An activity slightly higher compared to the empty vector *pALiCE02* control was observed for verbascose (+36.3%), arabinogalactan from larch wood (+24.2%) and AGPs from *A. thaliana* cell suspension cultures (+30.6%) (**Figure 5B**). The modest activity may be due to the inherently low abundance of α-D-Gal*p* and β-L-Ara*p* moieties in AGP *O*-glycans (Tryfona et al., 2012). Furthermore, AGAL activity is typically lower for RFOs with a higher DP (**Supplementary File S8.5**).

Similar to AtAPSE, GH27_OsAPSE shows high activity for substrates which are not colocalizing with OsAPSE at the cell surface (Imaizumi et al., 2017). RFOs do not occur at the cell surface of rice cells (De Coninck et al., 2024a), but are stored in vacuoles (Van den Ende, 2013; Elango et al., 2022). Galactomannan is speculated to be present in low quantities in the cell walls of rice endosperm and the aleurone layer (Ren et al., 2007). However, GH27 activity was also detected for AGPs with *O*-glycans (**Figure 5B**).

GH27 enzymes from plants typically accommodate hydrolytic cleavage of monosaccharides from storage polysaccharides or cell wall structures (Gao and Schaffer, 1999; Imaizumi et al., 2017; Chuankhayan et al., 2023). Most of the arabinose and galactose at the cell surface occurs as α/β-L-Ara*f* and β-D-Gal*p* (Ghosh et al., 2023) and is present in arabinogalactan, rhamnogalacturonan I and xylan side chains. Theoretical models depicting cell wall structure do not always include β-L-Ara*p* and α-D-Gal*p* (Seifert, 2020; Delmer et al., 2024) despite the fact that these residues have been detected by NMR at. the extremities of AGP *O*-glycans (Odonmazig et al., 1994; Ponder and Richards, 1997; Strasser et al., 2021) or in side chains of pectin rhamnogalacturonan-I (Perez, 2003; Caffall and Mohnen, 2009; Goetz et al., 2016). The structure (and function) of AGP *O*-glycans and pectic polysaccharides depends on the activity of CAZymes, such as glycosyltransferases for synthesis and GHs for trimming and degradation (Leszczuk et al., 2023). Both AGP *O*-glycans and pectic polysaccharides are highly complex and heterogenous structures, and the relationship between glycan/polysaccharide structure and biological function is not fully understood (Strasser et al., 2021). In plants, most of the transferases for glycan/pectin synthesis are known (Caffall and Mohnen, 2009; Silva et al., 2020) while far less information is available concerning *O*-glycan degradation in plants (Ellis et al., 2010), since only 5 AGP *O*-glycan degrading GHs are currently known (Knoch et al., 2014).

The composition and modification of AGP *O*-glycans vary significantly across cell types, tissues and species, suggesting that plants modify these structures to their environment and physiological needs (Leszczuk et al., 2023). Although not fully understood, evidence indicates that AGP *O*-glycan structure is linked to biological function. AGP *O*-glycans help plants respond to environmental stresses. For example, the seagrass *Zostera marina* produces *O*-glycans rich in 4-*O*-methylglucuronic acid, which provides a polyanionic interface and contributes to osmotic adjustment to salinity stress (Pfeifer et al., 2020). Such *O*-glycosylation patterns are not observed in land plants, which mostly obey to the typical ‘type-II AGP’ structure (Strasser et al., 2021). In land plants, fungal degradation of AGP *O*-glycans can impair cellulose production and growth, highlighting the importance of glycan length and branching for cell wall integrity (Kikuchi et al., 2022). AGP *O*-glycans also stabilize the cell wall by binding ions like Ca^2+^ through negatively charged residues (*f.i.* glucuronic acid). Plants with reduced glucuronidation show severe developmental issues, which can be alleviated by Ca^2+^ supplementation, indicating that ion binding plays a structural role (Lopez-Hernandez et al., 2020). Additionally, AGP *O*-glycans form covalent links with other cell wall components like pectic rhamnogalacturonan-I and arabinoxylan, contributing to structural stability of the cell wall matrix (Tan et al., 2013, 2023). Finally, AGP *O*-glycans are also crucial in development, influencing cell division, elongation and differentiation. For instance, trimming of AGP O-glycans affects apple fruit ripening (Leszczuk et al., 2020), and proper glycosylation by hydroxyproline-*O*-galactosyltransferases is essential for pollen development, as shown by sterility in *Arabidopsis* mutants lacking these enzymes (Kaur et al., 2022).

### 3.4 OsAPSE may be unconventionally secreted to the cell surface

#### 3.4.1 Arguments for unconventional protein secretion

OsAPSE is predicted to be synthesized with an *N*-terminal SP. No other localization signals are detected (**Supplementary File S9.1**). Because of the presence of a SP, it can be expected that OsAPSE will follow the secretory pathway involving protein synthesis on the endoplasmic reticulum (ER). Furthermore, PTMs such as the addition of *N*-glycans and the formation of disulfide bridges are likely to occur. Asparagine residues N269, N372 and N380 occur in a sequon and are predicted as *N*-glycosylation sites with high confidence (**Supplementary File S9.1**). However, N380 is part of a NPT sequon and will therefore not be recognized by the oligosaccharyltransferase complex (Matsumoto et al., 2017). The sequons at N269 and N372 are likely to accommodate *N*-glycans as these asparagine residues are correctly oriented at the protein surface (**Supplementary File S9.2**). Several disulfide bridges are predicted in the structure of OsAPSE, although only 1 disulfide bridge is likely to occur in the GH27 domain of OsAPSE. Cysteine residues C187 and C227 are positioned under a favorable dihedral angle and inter-atomic distance of 2.03 Å, which is within the average disulfide bridge length of 1.8-3.0 Å.

It remains speculative whether or not secretion of OsAPSE occurs conventionally or unconventionally. By default, it is assumed that proteins with a SP are secreted at the cell surface through conventional secretion (Rose and Lee, 2010). However, it was shown that several secretory proteins do not necessarily possess a SP (Wang et al., 2018). Unconventional protein secretion in multivesicular bodies has been demonstrated for several cell wall-active enzymes, including xyloglucan endotransglucosylase/hydrolases from *Arabidopsis* (De Caroli et al., 2021) and HaAPSE (HanXRQChr08g0208381), the *Helianthus annuus* (sunflower) homologue of OsAPSE (50.2% sequence identity) (Regente et al., 2017). Retrieving cell wall-active enzymes from multivesicular bodies is significant and remarkable, as it was supposed for a long time that these enzymes are trafficked by a SP and secreted conventionally (Rose and Lee, 2010). However, it has been hypothesized that some cell wall-active enzymes with SP may be delivered at the cell surface using a vesicular “type IV-UPS” bypass of the Golgi apparatus (Rabouille, 2017; Maricchiolo et al., 2022). Alternatively, unconventionally secreted cell wall-active enzymes with SP may bypass both the Golgi apparatus and vesicular transport through ER-plasma contact sites (Bellucci et al., 2018). Unfortunately, in contrast to mammalian proteins, tools for predicting unconventional protein secretion, such as SecretomeP (RRID: SCR_026505), are not available for plants (Bendtsen et al., 2004).

#### 3.4.2 Localization of OsAPSE according to *in silico* prediction tools

OsAPSE was predicted to be located at the cell surface or cell wall (**Supplementary File S9.3**). The results were mainly uniform, predicting the localization of OsAPSE. This is in agreement with the hypothesized function of OsAPSE being a cell wall-active enzyme. Localization data from the WallProtDB-2 database (San Clemente et al., 2022) (RRID: SCR_026506) reveals that OsAPSE has already been detected in the cell wall proteome of rice callus cultures (Chen et al., 2009).

#### 3.4.3 Localization of OsAPSE-EGFP in transiently transformed *N. benthamiana* leaves

**Figure 6:**
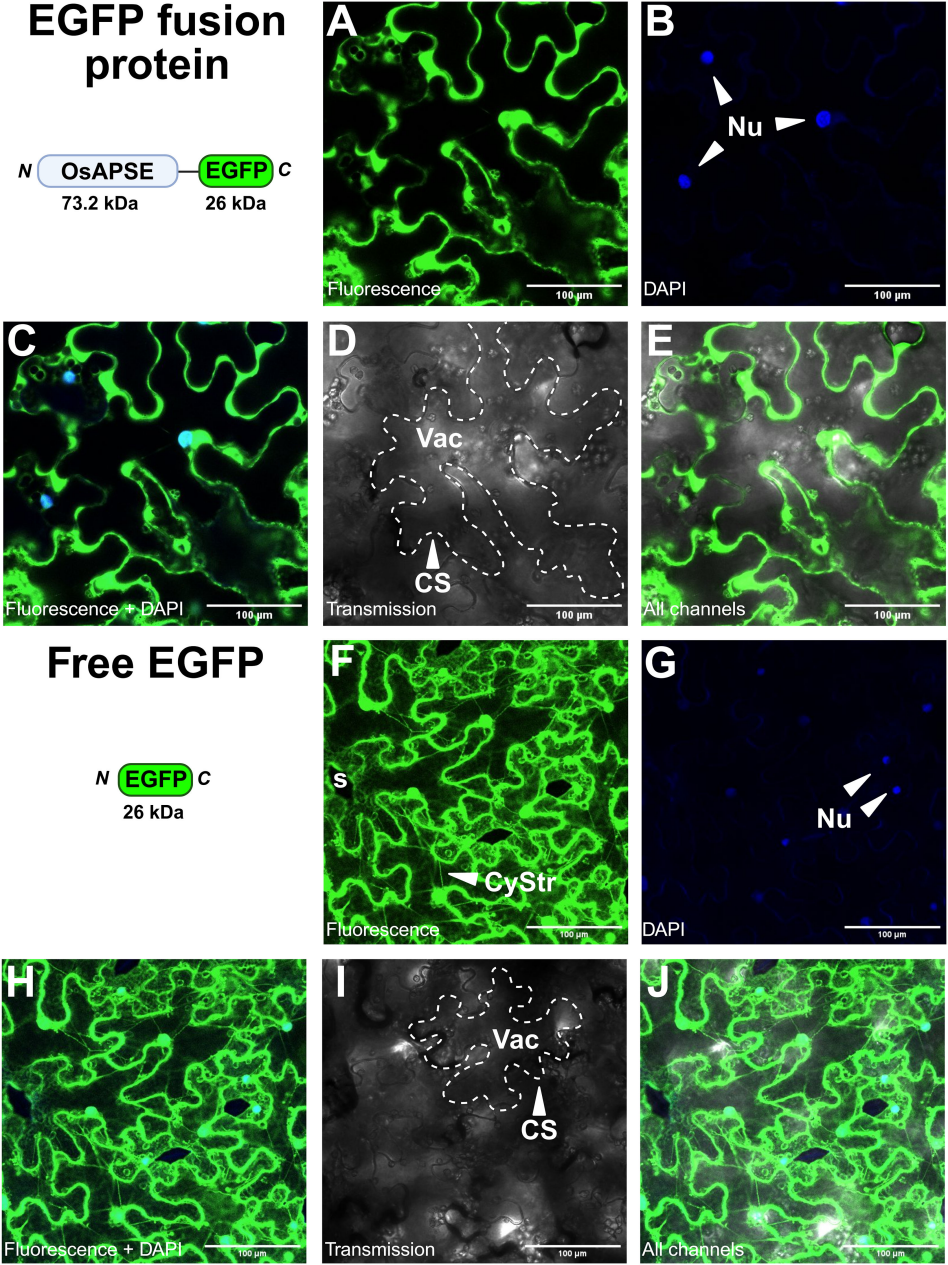
Subcellular localization of OsAPSE-EGFP in epidermal cells of *Nicotiana benthamiana* leaves. **(A-E)** show *pK7FWG2::OsAPSE-EGFP* infiltrated tissues, while **(F-J)** show tissues infiltrated with the *pK7FWG2* empty vector control. The domain architecture, orientation and size of the transiently produced protein is shown. **(A, F)** show the green fluorescent signal. **(B, G)** show the DAPI signal. **(C, H)** show the combined green fluorescent and DAPI signal. **(D, I)** show the transmission channel. **(E, J)** show the combination of all channels. Abbreviations: CS (cell surface), CyStr (cytoplasmic strand), Nu (nucleus), s (stomata), Vac (vacuole).

Subcellular localization studies of OsAPSE in *N. benthamiana* leaves, transiently transformed with the *pK7FWG2::OsAPSE-EGFP* fusion construct, reveal that the OsAPSE-EGFP fusion protein is localized at the cell surface (**Figures 6A–E**). No fluorescent signals were detected in the cytosol. Transient expression of cytosolic EGFP-fusion proteins typically shows cytoplasmic strands, attributed to the voluminous (*i.e.* 80-90% of total volume) vacuoles of the tobacco epidermal cells (Cui et al., 2020). Cytoplasmic strands were abundantly observed in the free-EGFP control treatments (**Figures 6F–J**), but only sporadically and faintly in *pK7FWG2::OsAPSE-EGFP* treated plants (**Figure 6A**). In several images, we also observed colocalization between OsAPSE-EGFP and the DAPI signal, suggesting a nuclear localization (**Figures 6B, C**) although no NLS was identified in the coding sequence of OsAPSE (**Supplementary File S9.2**).

Heterologous expression of non-native SPs is often less efficient (Jarvis et al., 1993; Wilbers et al., 2016), due to the occurrence of certain amino acids in the SP (*f.i.* double arginine and multiple repeated proline residues) that hinder helix formation, lower the affinity towards the SP recognition particle and inhibit SP peptidases (Nilsson and Von Heijne, 1992; Snapp et al., 2017). The SP of OsAPSE contains such disturbing elements (-PPPWRRLLRCALLPP-). RR motifs in SPs are ER retention signals, due to their ability to interfere with vesicle formation that would otherwise transport the protein along the secretory pathway (Schutze et al., 1994). Disturbed recognition or incomplete SP cleavage results in subpopulations of the protein of interest being either processed adequately or accumulating in the ER (Wilbers et al., 2016). Protein accumulation in the ER in turn leads to ER stress and unfolded protein responses (Hicks, 2013; Srivastava et al., 2014), which involve ER-associated degradation and retro-translocation of the misfolded protein to the cytosol for degradation (Johnson and Haigh, 2000). Upon proteolytic cleavage of the misfolded proteins, hidden NLS sequences are often exposed, causing nuclear import (Srivastava et al., 2014). Likewise, cytosolic degradation of (partially) misfolded OsAPSE-EGFP could result in the release of EGFP, which localizes by default to the nucleus (Seibel et al., 2007). Such ‘protein reflux’ has been described in the context of ER stress (El Meskini et al., 2001). Noteworthy, it has been established that EGFP(-fusion) proteins with size up to 110 kDa may diffuse spontaneously into the nucleus (Wang and Brattain, 2007).

Alternatively, it is possible that the native SP of OsAPSE is recognized both as a SP and a NLS. NLS sequences are typically hydrophobic (Kiefer et al., 1994). The activity of background proteases in *N. benthamiana* (Jutras et al., 2020) may trim the native SP, explaining NLS recognition. Similar cases in which *N*-terminal sequences were recognized simultaneously as SP and NLS have been reported, albeit in animals (Kiefer et al., 1994), although NLS sequences are functionally conserved amongst higher eukaryotes (Wagner and Hall, 1993; Hicks, 2013). In these cases, the protein of interest had a dual fate, in both the secretory pathway and nucleus (Kiefer et al., 1994; Iwata et al., 2008). It should be emphasized that not all NLS sequences in plants have been discovered, as they are often non-canonical and not defined by a consensus sequence (Hicks, 2013; Lu et al., 2021).

Further investigation of the subcellular localization at the cell surface using propidium iodide as organelle marker for the plasma membrane did not deliver reliable results (data not shown). The combination of *in silico* predictions for OsAPSE localization, occurrence of OsAPSE in cell wall proteome databases and the microscopy images suggest that OsAPSE is most likely localized at the cell surface.

### 3.5 OsAPSE is involved in germination of rice seeds by acting on cell walls

#### 3.5.1 Screening of transgenic and mutant rice plants

**Figure 7:**
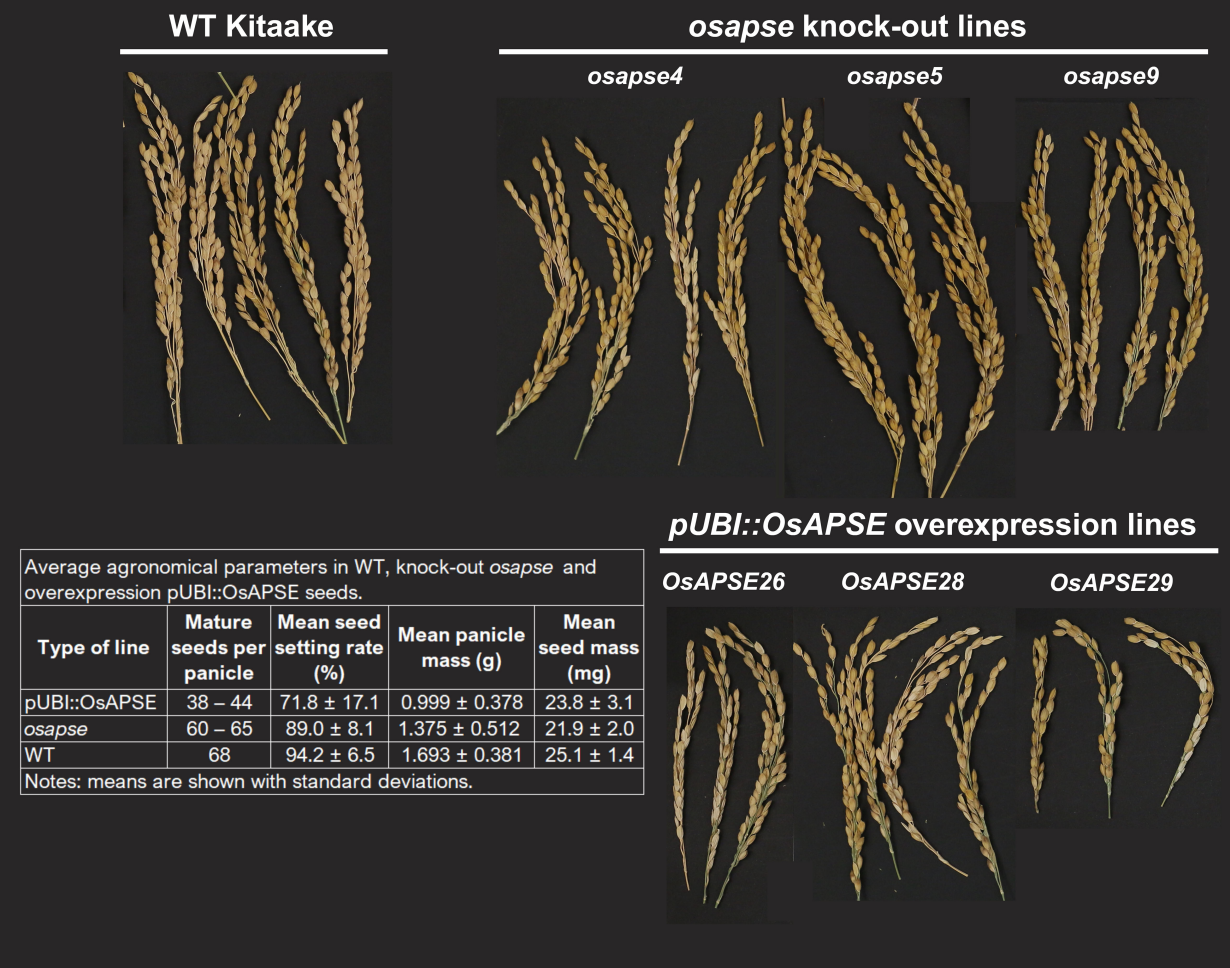
Representative panicles and average agronomical parameters of wild type, overexpression *pUBI::OsAPSE* and knock-out *osapse* rice seeds.

Transgenic rice lines with overexpression (*pUBI::OsAPSE*) or gene knock-out (*osapse*) were genotyped (**Supplementary File S10**) and analyzed for agronomical qualities. *pUBI::OsAPSE* panicles contained less mature seeds and showed lower seed setting rates compared to WT and *osapse* seeds, while panicle mass and seed mass where highest for WT seeds compared to *osapse* and *pUBI::OsAPSE* panicles and seeds (**Figure 7**). The agronomical parameters are in accordance with the model of Smith & Fretwell (Smith and Fretwell, 1974) and indicate the high plasticity of seed number and limited variation in seed mass (**Supplementary File S11**).

#### 3.5.2 OsAPSE is involved in rice seed germination and seedling development

The involvement of OsAPSE in seedling development and seed formation was inferred from both *in vitro* rice seed germination assays as well as RT-qPCR experiments on germinating and developing seeds and seedlings. Results from RNA extraction, cDNA synthesis, RT-qPCR data and normalization of gene expression are included in **Supplementary File S12**.

**Figure 8:**
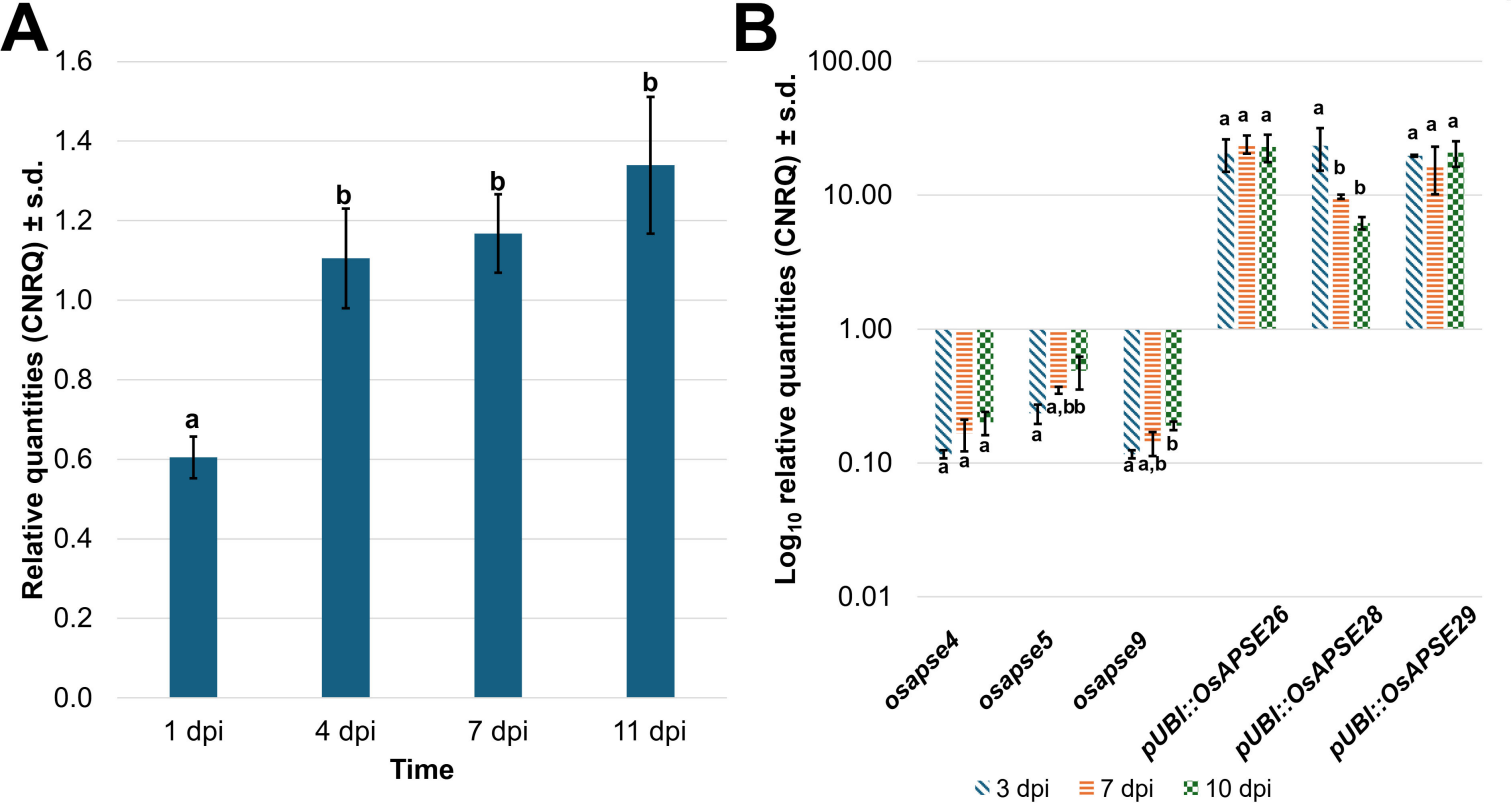
*OsAPSE* transcript levels in developing rice seedlings. Transcript levels for *OsAPSE* in germinating WT seeds and seedlings **(A)**. Transcript levels for *OsAPSE* in germinating seeds and seedlings of knock-out and overexpression plants **(B)**. Transcript levels are shown as calibrated normalized relative quantities. Homogenous subsets, based on Tukey *post-hoc* ANOVA analysis are indicated with letters a and b.

**Figure 8** shows the *OsAPSE* transcript levels in developing WT (**Figure 8A**), mutant *osapse* and overexpressing *pUBI::OsAPSE* seedlings (**Figure 8B**). In WT seedlings, the transcript levels for *OsAPSE* are low but show an increase over time (**Figure 8A**). The *OsAPSE* transcript levels are drastically lowered in *osapse* mutants, reaching an overall average transcript level of 0.22 ± 0.12 CNRQ (calibrated normalized relative quantities) during rice seed germination. The low *OsAPSE* transcript levels in knock-out mutants illustrate that *osapse* plants are true knock-out mutants (one-way ANOVA, F = 60.280; ν = 75 df, p < 0.001, η² = 0.840) compared to WT and *pUBI::OsAPSE*. Similar to WT seedlings, the *OsAPSE* transcript levels in *osapse* mutants increase slightly over time (**Figure 8B**) (one-way ANOVA, F = 20.037; ν = 14 df; p < 0.001, η² = 0.845). Elevated and generally stable *OsAPSE* transcript levels (on average 18.23 ± 5.94 CNRQ) are observed for *pUBI::OsAPSE* overexpression lines, although the transcript levels in *pUBI::OsAPSE* 28 show a decrease over time. An apparent increase in *OsAPSE* transcription, though not statistically different, was observed in WT and is mainly attributed to the increased metabolic activity in developing seedlings. Indeed, it was already reported that the transcript levels for several CAZymes including AGALs and β-D-mannosidases increase during rice seed germination (Ren et al., 2007).

Knock-out mutants of *O*-glycan-active enzymes and cell wall-active enzymes often yield aberrant phenotypes (*f.i.* prolonged roots, reduced hypocotyls) (Eudes et al., 2008; Nibbering et al., 2020; Dash et al., 2023), as was also observed for *atapse* knock-out plants (Imaizumi et al., 2017). Despite the strong structural similarity between OsAPSE and AtAPSE, we did not observe differences in coleoptile length in *osapse* or *pUBI::OsAPSE* seedlings (data not shown).

**Figure 9:**
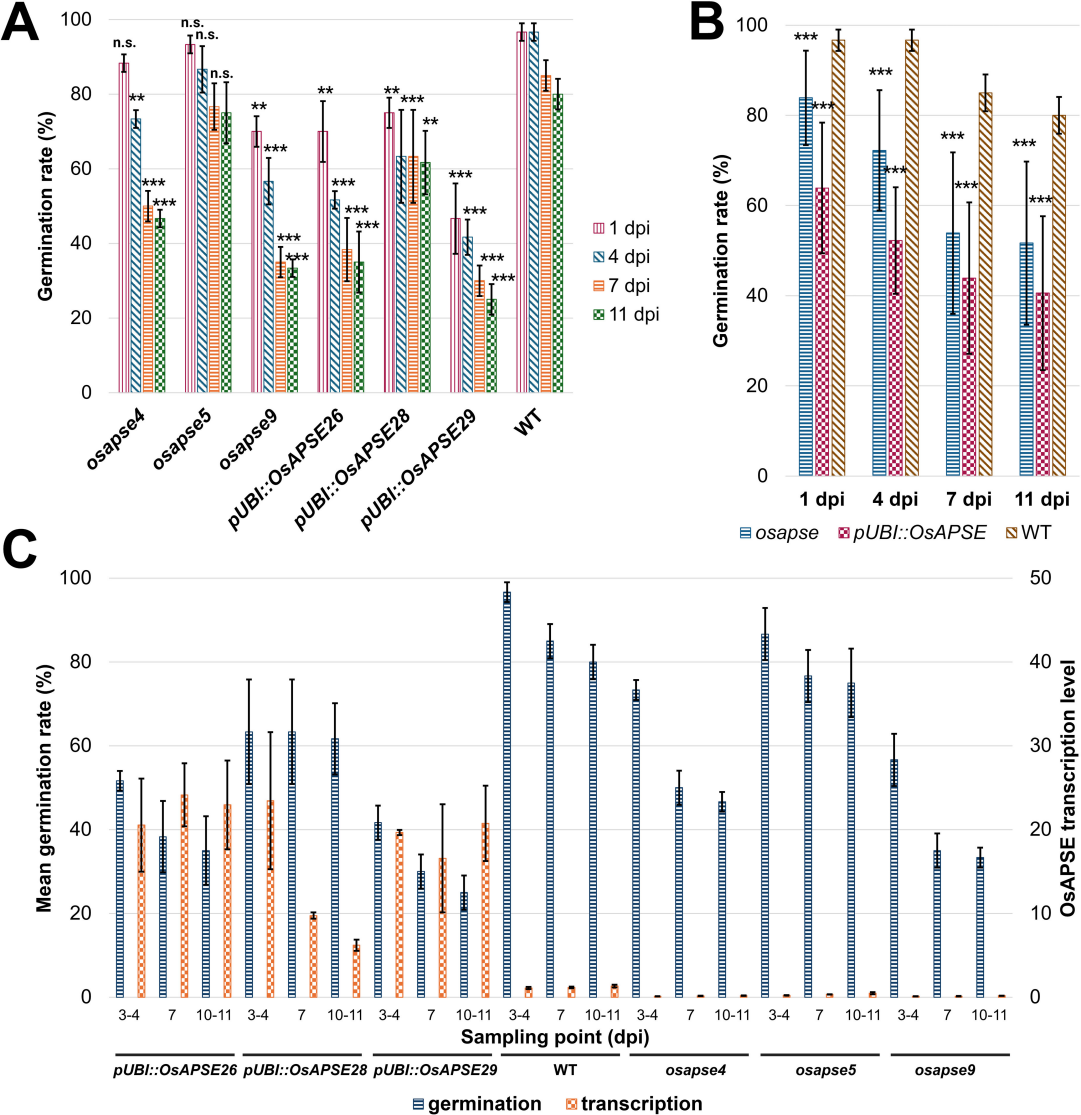
Seed germination rates of mutant *osapse* and overexpressing *pUBI::OsAPSE* rice compared to wild type plants. Germination rates across mutant *osapse*, overexpressing *pUBI::OsAPSE* and wild type rice at 1 dpi, 4 dpi, 7 dpi and 11 dpi **(A)**. Average germination rates for knock-out lines and overexpression lines compared to WT **(B)**. Correlation between the transcript level of *OsAPSE* and the observed rice seed germination rate at time points 3–4 dpi, 7 dpi and 10–11 dpi **(C)**. Asterisks indicate the significance level: *(p < 0.05), **(p < 0.01), ***(p < 0.001). Error bars are standard deviations originating from 3 biological replicates of 20 rice seeds per replicate. Abbreviations: n.s. (non-significant difference: p ≥ 0.05).

Differences in germination rates were apparent between *osapse*, *pUBI::OsAPSE* and WT seeds across different time points (**Supplementary File S13**) (**Figure 9**). The Omnibus Test of Model Coefficients revealed that the constructed GLM, comprising the relationship between sampling time, line and observed germination rate was significant (χ² = 343.460, *ν* = 27 df, p < 0.001). The GLM displayed main effects from the sampling time (χ² = 84.672, *ν* = 3 df, p < 0.001) and type of line (χ² = 251.142, *ν* = 6 df, p < 0.001), but not the interaction (χ² = 18.488, *ν* = 18 df, p < 0.424). Interestingly, *pUBI::OsAPSE* and knock-out *osapse* lines displayed a lower germination rate compared to WT, which consistently achieved highest germination rates (80-97%) at the different time points (**Figure 9A**). The transgenic *pUBI::OsAPSE* lines and knock-out *osapse* lines demonstrated aberrant germination phenotypes. Especially at time points 1 dpi and 4 dpi, large fractions of the *pUBI::OsAPSE* seeds (40-70%) and *osapse* seeds (40-75%) displayed seed lethality, no radicle emergence and disturbed root development, arrested coleoptile elongation and seedling etiolation, while WT seeds showed normal development, with emerging leaves and root formation. Knock-out line *osapse5* displayed germination rates that were most similar to WT, while lines *pUBI::OsAPSE28*, *pUBI::OsAPSE29*, *osapse9* and *osapse4* demonstrated the most deviating germination rates (**Figure 9A**). In general, the seed germination rates of *osapse* knock-out lines were higher compared to *pUBI::OsAPSE* overexpression lines (**Figure 9B**), but still lower compared to WT seeds. The germination behavior of *osapse4* and *osapse5* seeds differed considerably, despite bearing identical mutations (**Supplementary File S10**). The differences between the overexpression lines are attributed to the independent nature of these lines, as each overexpression line is created by a single transformation event, leading to specific *OsAPSE* transcript levels. Interestingly, data from the germination assay and RT-qPCR experiments suggest that optimal *OsAPSE* transcription levels are required for rice seed germination (**Figure 9C**). There are several examples of genes with similar expression regulation patterns. *OsMADS1* from rice involved in flower development and pollen morphology (Liu et al., 2023, 2025), *AtWUS* from *Arabidopsis* involved in shoot apical meristem maintenance and floral development (Stephenson et al., 2019; Li et al., 2023) and *ZmCCT10* from maize involved in photoperiod sensitivity and regulation of flowering time (Ikeda et al., 2009; Yadav et al., 2011), exemplify genes whose expression must be finetuned to ensure normal development. In each case, both knock-out and overexpression lead to aberrant phenotypes, ranging from sterility and disrupted organ formation to severe architectural defects, highlighting their dosage-sensitive nature, similar to OsAPSE.

#### 3.5.3 Involvement of OsAPSE in cell wall metabolism during rice seed germination

The involvement of GH27 proteins in seed germination or seedling development has been reported in pea (Blöchl et al., 2008), chickpea (Arunraj et al., 2020), cluster bean (Hughes et al., 1988), soybean (Guimaraes et al., 2001; Lien et al., 2018) and vetch (Gojło, 2023). Often, AGALs are involved in the germination process through their ability to hydrolyze storage polysaccharides (Elango et al., 2022) and galactomannan (Sharma et al., 2022), after which the released D-Gal*p* moieties are epimerized to D-Glc*p* and used as energy source (Zhang et al., 2006) or act as extracellular signaling molecules (Showalter, 2001).

**Figure 10:**
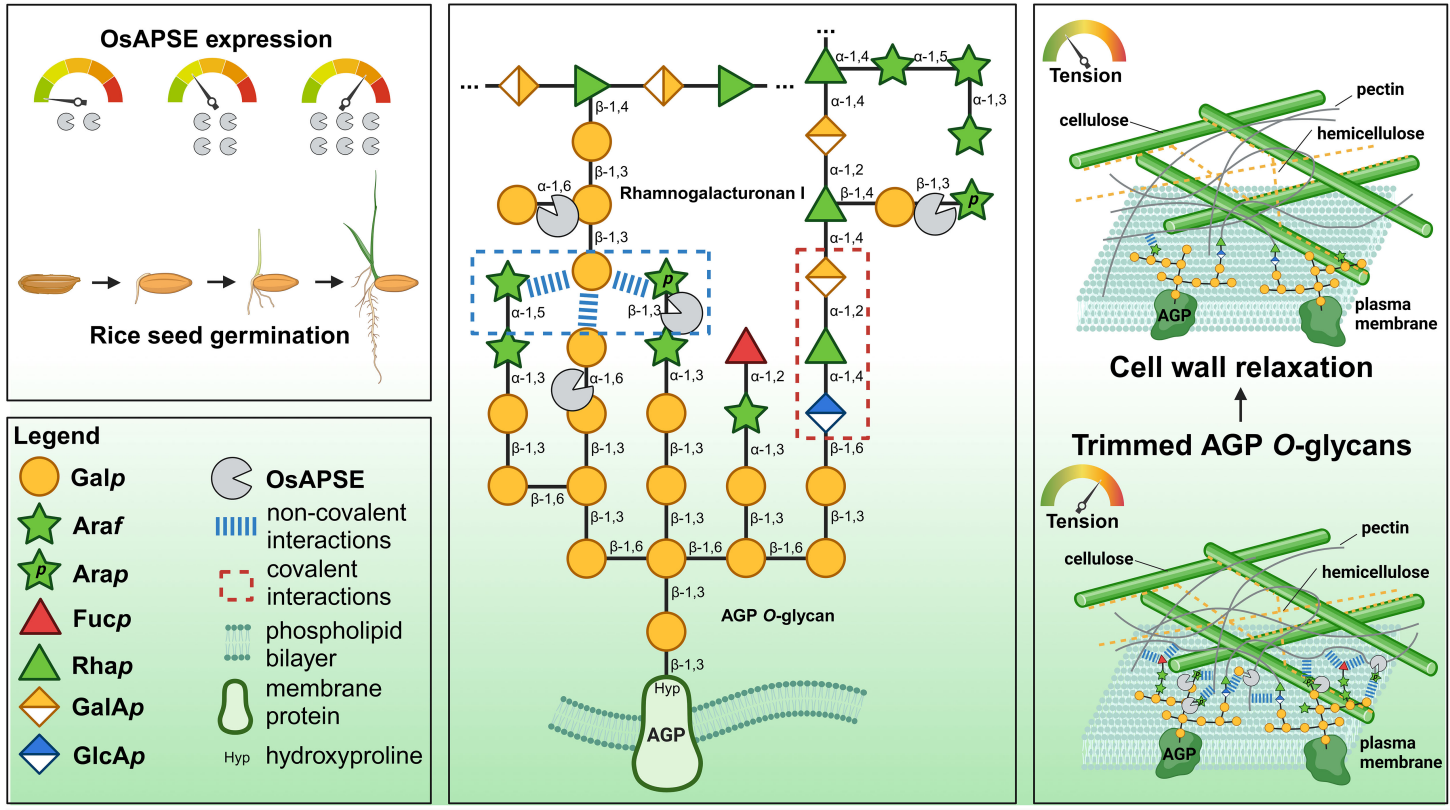
Working hypothesis for OsAPSE involvement in cell wall metabolism during rice seed germination. OsAPSE displays AGAL and ARAP activity, acting on the pectin-AGP *O*-glycan network, thereby affecting non-covalent interactions and impacting cell wall flexibility required for cell growth and expansion. AGP *O*-glycan structure and representation of the primary cell wall are based on existing theoretical models (Carpita and Gibeaut, 1993; Odonmazig et al., 1994; Ponder and Richards, 1997; Perez, 2003; Caffall and Mohnen, 2009; Goetz et al., 2016; Seifert, 2020; Strasser et al., 2021; Tan et al., 2023). This diagram was created using BioRender.com.

We hypothesize that adequate *OsAPSE* transcription levels are required for normal rice seed germination. Deviating transcript levels, as observed in *osapse* and *pUBI::OsAPSE* plants, likely cause metabolic imbalances, with repercussions for seed germination capacity. The importance of OsAPSE for the germination process is explained by dual ARAP/AGAL activity on cell wall structures. We assume that OsAPSE displays AGAL/ARAP activity on α-D-Gal*p* and β-L-Ara*p* residues occurring along the continuous pectin (rhamnogalacturonan-I)-AGP *O*-glycan network (**Figure 10**) (Tan et al., 2013, 2023). The ricin-B-like domain can enhance substrate binding since ricin-B(-like) domains and related CBM13 modules are known to recognize specifically D-Gal*p* and D-GalNAc residues (Hazes, 1996; Steeves et al., 1999), but can also recognize L-Ara*p* residues due to the structural similarity between D-Gal*p* and L-Ara*p* as demonstrated in a GH27 ARAP from *Streptomyces avermitilis* (Ichinose et al., 2009; Fujimoto, 2013).

Removal of terminal α-D-Gal*p* and β-L-Ara*p* moieties from AGP *O*-glycans and rhamnogalacturonan-I likely abolishes non-covalent interactions between the AGP *O*-glycan and the pectin fraction of the primary cell wall (**Figure 10**). Removal of these residues contributes to structural reorganization of the cell wall, as pectic polysaccharides and AGPs are in turn directly tethered to cellulose microfibrils and hemicellulose polysaccharides (Ellis et al., 2010; Peaucelle et al., 2012; Jamet and Dunand, 2020). Weakened interactions between AGP *O*-glycans and pectin reduce cell wall tension and allow cells to elongate and expand under influence of turgor pressure (**Figure 10**). The required degree of mobility between compounds of the primary cell wall and AGPs could be regulated (*i.e.* tightening and loosening of non-covalent interactions) through well-dosed activity of CAZymes like *OsAPSE*. Flexibility and extensibility of cell wall constituents is of paramount importance to attain normal cell elongation, cell expansion and growth in general (Cosgrove, 2024), especially in fast-paced processes like seed germination (Wolny et al., 2018; Yang et al., 2020).

## 4 Conclusions

This study focused on the biological function of OsAPSE, a member of the GH27 family from Japanese rice. Using a multi-perspective approach, we aimed to decipher the biological relevance of this protein.

We have demonstrated the intriguing phylogeny of OsAPSE, which is based on the presence of a ricin-B-like domain (De Coninck et al., 2024b). The presence of this lectin domain is only observed in a subset of GH27 sequences from *Viridiplantae* and distinguishes APSE homologues from other GH27 members. A subdivision as suggested by the CUPP database would therefore be appropriate (Barrett et al., 2020). Likewise, the GH27 domain of OsAPSE and other APSE homologues is structurally different compared to regular GH27 domains, with RMSD values > 5Å.

We also provided insights into the biochemical characteristics and putative biological function of OsAPSE. Although we did not succeed in obtaining soluble recombinant OsAPSE or its subdomains in bacterial or yeast cells, we were successful in the production of small quantities of the OsAPSE GH27 domain using a cell-free system. The produced proteins showed clear AGAL and ARAP activity, with optimal activity at pH = 8 and 25°C. Although the optimum pH differed from the typical apoplastic pH (5.5-6), we assume this is attributable to enzyme dormancy and activation upon cell wall alkalinization, typically associated with regulation of cell wall loosening and expansion (Geilfus et al., 2017). We calculated the Michaelis constant for pNP-α-D-Gal*p* as substrate, *i.e.* K_M_ = 0.67 mM, in line with observations from other plant GH27 enzymes. To our knowledge this study is the first to report enzymatic parameters for plant GH27 enzymes produced by a CFPS platform. Highest GH27 activity was present for melibiose, galactomannan and raffinose, while lower activities were obtained for verbascose, glycosylated arabinogalactan proteins and arabinogalactan. The activities observed for natural substrates were somewhat unexpected, as higher activities were obtained for substrates that will never make contact with OsAPSE at the cell surface. Melibiose does not occur in rice and galactomannan and raffinose are only present in low quantities in specific cells (*f.i.* endosperm cells, vascular tissue) (Ren et al., 2007; Van den Ende, 2013; Li et al., 2018; Elango et al., 2022). A similar observation for AtAPSE was made in the past (Imaizumi et al., 2017).

Subcellular localization analyses *in planta* suggest the localization of OsAPSE-EGFP at the cell surface but needs confirmation in future experiments. Co-localization studies with organelle reporters for the ER, Golgi apparatus and plasma membrane could be considered, in combination with plasmolysis assays, to pinpoint the exact subcellular localization of OsAPSE (Serna, 2005; Stellmach et al., 2022).

Transcriptomics analyses in WT rice revealed that *OsAPSE* transcript levels double during the germination process, indicating the need for GH27 activity during seed germination. The germination rate of rice is negatively affected when *OsAPSE* transcript levels are decreased or increased. Interestingly, the agronomical traits including mature seeds per panicle, the setting rate, the panicle mass and average seed mass were usually lower compared to WT plants. The importance of OsAPSE for the rice seed germination process is mainly explained by its proposed dual enzymatic activity along the continuous pectin–AGP *O*-glycan network (Tan et al., 2023). Based on the observed AGAL and ARAP activity and the ability of OsAPSE to cleave off β-L-Ara*p* and α-D-Gal*p* moieties from cell wall structures, we hypothesize that OsAPSE displays dual activities on β-L-Ara*p* and α-D-Gal*p* moieties along the continuous pectin-AGP *O*-glycan network at the cell surface, while the ricin-B-like domain might enhance substrate binding. The activity of OsAPSE likely affects non-covalent interactions between the pectin fraction and AGP *O*-glycans, either *in muro* or anchored in the plasma membrane (Ellis et al., 2010; Nibbering et al., 2020). As a result, altered non-covalent interactions would allow increased or decreased attraction between AGP *O*-glycans and pectin, thereby enhancing cell wall loosening and relaxation, which is a key driver in cellular expansion and growth (Cosgrove, 2024). Seed germination is *par excellence* a fast-paced developmental process, characterized by rapid cellular growth, and therefore a strong need for rapid cell wall metabolism (Wolny et al., 2018). Rapid and developmentally controlled AGP turnover, mediated by CAZymes, has been described in germinating rice seeds (Lu et al., 2001; Yuan et al., 2008). We have shown that the absence or excess of *OsAPSE* transcripts leads to aberrant germination phenotypes. Shortage of *OsAPSE* transcripts may lead to persisting α-D-Gal*p* and β-L-Ara*p* residues and therefore also more persisting non-covalent interactions between AGP *O*-glycans and pectin, resulting in reduced cell wall flexibility, while increased *OsAPSE* transcripts could result in excessive detachment. We therefore believe that OsAPSE may be a key enzyme in enabling cell wall relaxation through regulating non-covalent interactions between pectin and AGP *O*-glycans during the rice seed germination process Future experiments will be required to further investigate the involvement of OsAPSE in seed germination. For instance, effects of defective arabinosyl- and galactosyltransferase activity during seed development and germination should be explored. The role of several GTs in AGP processing have been elucidated in *Arabidopsis*, but remain elusive in rice (Silva et al., 2020; Strasser et al., 2021). Furthermore, we recommend future research to focus on the enzymatic properties of AGP *O*-glycan active enzymes. For instance, the characterization of AGP *O*-glycans and the activity of OsAPSE on AGP *O*-glycans can be investigated, combined with HPAEC-PAD for quantification and NMR and MS/MS for determining the saccharide composition and glycosidic linkages (Tan et al., 2024). It should be emphasized that the function of OsAPSE may not be exclusively connected to seed germination, as AGP *O*-glycans are involved in a plethora of physiological processes (Ellis et al., 2010; Mareri et al., 2019; Strasser et al., 2021) including growth and development (Hromadová et al., 2021; Lamport et al., 2021), cell differentiation (Borassi et al., 2020), cellular communication (Lopez-Hernandez et al., 2020; Teh et al., 2022), reproduction (Kaur et al., 2022) and signaling of biotic (Kikuchi et al., 2022) and abiotic (Pfeifer et al., 2020) stresses.

## Data Availability

The original contributions presented in the study are included in the article/**Supplementary Material**. Further inquiries can be directed to the corresponding author/s.

## Footnotes

1
https://pubchem.ncbi.nlm.nih.gov/.

2
https://www.chemspider.com/.
