# Supplementary material for: OsAPSE modulates non-covalent interactions between arabinogalactan protein O-glycans and pectin in rice cell walls
Source: Front Plant Sci. 2025 May 22;16:1588802. doi: 10.3389/fpls.2025.1588802 (PMC12137362; doi:10.3389/fpls.2025.1588802)
Supplement: Supplementary file 1 [file Table1.docx]

**Supplementary File S1 – Examples of characterized GH27 enzymes across kingdoms**

| **Activity and EC number** | **Organism** | **Taxonomy** | **Substrates** | **Source** |
| --- | --- | --- | --- | --- |
| AGAL/MEL  (EC: 3.2.1.22) | *Homo sapiens* | Metazoa | Lysosomal glycosphingolipids | (Lukas et al., 2016) |
|  | *Cellvibrio japonicus* | *Bacteria* | Galactomannan | (Halstead et al., 2000) |
|  | *Cucumis melo* | *Viridiplantae* | Stachyose, raffinose, melibiose | (Gao and Schaffer, 1999) |
|  | *Saccharomyces cerevisiae* | *Fungi* | Raffinose, melibiose | (Fernández-Leiro et al., 2010) |
| NAGA  (EC: 3.2.1.49) | *Homo sapiens* | *Metazoa* | Blood group A antigen, Forssman antigen, mucin glycoproteins, glycolipids | (Clark and Garman, 2009) |
|  | *Acremonium* spp. | *Fungi* | Blood group A antigen | (Kadowaki et al., 1989) |
| ARAP  (EC: 3.2.1.88) | *Chitinophaga pinensis* | *Bacteria* | Arabinogalactan (protein *O*-glycans) | (McKee and Brumer, 2015) |
|  | *Arabidopsis thaliana* | *Viridiplantae* | Arabinogalactan (protein *O*-glycans) | (Imaizumi et al., 2017) |
|  | *Fusarium oxysporum* | *Fungi* | Arabinogalactan (protein *O*-glycans) | (Sakamoto et al., 2010) |
| IMDase  (EC: 3.2.1.94) | *Arthrobacter globiformis* | *Bacteria* | Dextran | (Okazawa et al., 2015) |
| GALT  (EC: 2.4.1.-) | *Bifidobacterium adolescentis* | *Bacteria* | Donor: pNP-α-D-Gal*p*  Acceptor: melibiose | (Van Laere et al., 1999) |
|  | *Arabidopsis thaliana* | *Viridiplantae* | Donor: raffinose  Acceptor: raffinose | (Chuankhayan et al., 2023) |
|  | *Ajuga reptans* | *Viridiplantae* | Donor: raffinose, stachyose, verbascose, galactinol  Acceptor: raffinose, stachyose, verbascose | (Tapernoux-Lüthi et al., 2004) |
|  | *Meyerozyma guilliermondii* | *Fungi* | Donor: melibiose  Acceptor: D-Glc*p*, D-Gal*p*, maltose, maltitol, 1,4-butandiol, L-Ara*p*, D-Xyl*p*, D-Rib*p*, D-Fuc*p*, L-Rha*p*. | (Hashimoto et al., 1995) |

**References**

Chuankhayan, P., Lee, R.-H., Guan, H.-H., Lin, C.-C., Chen, N.-C., Huang, Y.-C., et al. (2023). Structural insight into the hydrolase and synthase activities of an alkaline α-galactosidase from *Arabidopsis* from complexes with substrate/product. *Acta Crystallogr. Sect. Struct. Biol.* 79, 154–167. doi: 10.1107/S2059798323000037

Clark, N. E., and Garman, S. C. (2009). The 1.9 Å Structure of Human α-N-Acetylgalactosaminidase: The Molecular Basis of Schindler and Kanzaki Diseases. *J. Mol. Biol.* 393, 435–447. doi: 10.1016/j.jmb.2009.08.021

Fernández-Leiro, R., Pereira-Rodríguez, Á., Cerdán, M. E., Becerra, M., and Sanz-Aparicio, J. (2010). Structural Analysis of Saccharomyces cerevisiae α-Galactosidase and Its Complexes with Natural Substrates Reveals New Insights into Substrate Specificity of GH27 Glycosidases. *J. Biol. Chem.* 285, 28020–28033. doi: 10.1074/jbc.M110.144584

Gao, Z., and Schaffer, A. A. (1999). A Novel Alkaline α-Galactosidase from Melon Fruit with a Substrate Preference for Raffinose. *Plant Physiol.* 119, 979–988. doi: 10.1104/pp.119.3.979

Halstead, J. R., Fransen, M. P., Eberhart, R. Y., Park, A. J., Gilbert, H. J., and Hazlewood, G. P. (2000). α-Galactosidase A from *Pseudomonas fluorescens* subsp. *cellulosa* : cloning, high level expression and its role in galactomannan hydrolysis. *FEMS Microbiol. Lett.* 192, 197–203. doi: 10.1111/j.1574-6968.2000.tb09382.x

Hashimoto, H., Katayama, C., Goto, M., Okinaga, T., and Kitahata, S. (1995). Transgalactosylation Catalyzed by α-Galactosidase from *Candida guilliermondii* H-404. *Biosci. Biotechnol. Biochem.* 59, 619–623. doi: 10.1271/bbb.59.619

Imaizumi, C., Tomatsu, H., Kitazawa, K., Yoshimi, Y., Shibano, S., Kikuchi, K., et al. (2017). Heterologous expression and characterization of an Arabidopsis β-l-arabinopyranosidase and α-d-galactosidases acting on β-l-arabinopyranosyl residues. *J. Exp. Bot.* 68, 4651–4661. doi: 10.1093/jxb/erx279

Kadowaki, S., Ueda, T., Yamamoto, K., Kumagai, H., and Tochikura, T. (1989). Isolation and Characterization of a Blood Group A Substance degrading α-N-Acetylgalactosaminidase from an Acremonium sp. *Agric. Biol. Chem.* 53, 111–120. doi: 10.1080/00021369.1989.10869270

Lukas, J., Scalia, S., Eichler, S., Pockrandt, A.-M., Dehn, N., Cozma, C., et al. (2016). Functional and Clinical Consequences of Novel α-Galactosidase A Mutations in Fabry Disease. *Hum. Mutat.* 37, 43–51. doi: 10.1002/humu.22910

McKee, L. S., and Brumer, H. (2015). Growth of Chitinophaga pinensis on Plant Cell Wall Glycans and Characterisation of a Glycoside Hydrolase Family 27 β-l-Arabinopyranosidase Implicated in Arabinogalactan Utilisation. *PLOS ONE* 10, e0139932. doi: 10.1371/journal.pone.0139932

Okazawa, Y., Miyazaki, T., Yokoi, G., Ishizaki, Y., Nishikawa, A., and Tonozuka, T. (2015). Crystal Structure and Mutational Analysis of Isomalto-dextranase, a Member of Glycoside Hydrolase Family 27. *J. Biol. Chem.* 290, 26339–26349. doi: 10.1074/jbc.M115.680942

Sakamoto, T., Tsujitani, Y., Fukamachi, K., Taniguchi, Y., and Ihara, H. (2010). Identification of two GH27 bifunctional proteins with β-L-arabinopyranosidase/α-D-galactopyranosidase activities from Fusarium oxysporum. *Appl. Microbiol. Biotechnol.* 86, 1115–1124. doi: 10.1007/s00253-009-2344-6

Tapernoux-Lüthi, E. M., Böhm, A., and Keller, F. (2004). Cloning, Functional Expression, and Characterization of the Raffinose Oligosaccharide Chain Elongation Enzyme, Galactan:Galactan Galactosyltransferase, from Common Bugle Leaves. *Plant Physiol.* 134, 1377–1387. doi: 10.1104/pp.103.036210

Van Laere, K., Hartemink, R., Beldman, G., Pitson, S., Dijkema, C., Schols, H., et al. (1999). Transglycosidase activity of Bifidobacterium adolescentis DSM 20083 α-galactosidase. *Appl. Microbiol. Biotechnol.* 52, 681–688. doi: 10.1007/s002530051579
