# Supplementary material for: OsAPSE modulates non-covalent interactions between arabinogalactan protein O-glycans and pectin in rice cell walls
Source: Front Plant Sci. 2025 May 22;16:1588802. doi: 10.3389/fpls.2025.1588802 (PMC12137362; doi:10.3389/fpls.2025.1588802)
Supplement: Supplementary file 2 [file Table2.docx]

**Supplementary File S2 – Overview of primer sequences in this study.**

| **Name** | **Orientation** | **Sequence** | **Purpose** | **Locus ID** | **Source** |
| --- | --- | --- | --- | --- | --- |
| P154 | Fw | ATGAAATTCTTAGTCAACGTTGC | Amplification of the pALiCE02 backbone. | *n.a.* | This study |
| P155 | Rv | CGTTATCGTACGCACCACGTGTGATTA |  |  |  |
| L903 | Fw | GCAAAACAATTCCTCTCCTGAC | qPCR target gene OsAPSE | LOC_Os01g33420 | This study |
| L904 | Rv | CACCTTGAAAACATGGGAAAC |  |  |  |
| A267 | Fw | GGAGCCGAAAGATGAAGTGC | Reference gene OsSAP18 (histone deacetylase complex subunit SAP18) | LOC_Os02g02960 | (Xu et al., 2015) |
| A268 | Rv | ACCCACCTCTTTGACAACGA |  |  |  |
| A269 | Fw | GAGCGGCAACACTAAGGATG | Reference gene OsFb15 (fiber protein Fb15) | LOC_Os02g07910 | (Xu et al., 2015) |
| A270 | Rv | AATCTCCTTGACGGCACAGA |  |  |  |
| Evd910 | Fw | TGTGAGCAGCTTCTCGTTTG | Reference gene OsEXP (N-lysine methyltransferase) | LOC_Os03g27010 | (Zhan et al., 2018) |
| Evd911 | Rv | TGTTGTTGCCTGTGAGATCG |  |  |  |
| L979 | Fw | TGCTCTGACAAGGGGACAAC | Amplification of the knock-out target region of OsAPSE | LOC_Os01g33420 | This study |
| L980 | Rv | GCATAACTGCCAAAGTGGCT |  |  |  |
| L983 | Fw | TTGGCGACCTCGTATTGGGAA | Amplification of the hygromycin resistance gene during selection of transgenic rice plants | *n.a.* | This study |
| L984 | Rv | CAAAGATCGTTATGTTTATCGGCACT |  |  |  |
| L301 | Fw | ATGGGAAGGGGAGCCCCAT | Amplification of OsAPSE (codon-optimized for *Nicotiana benthamiana*) | LOC_Os01g33420 | This study |
| L305 | Rv | ACATGTAATTTCAAACACCATGGAACCATG |  |  |  |
| L366 | Fw | AAAAAGCAGGCTTCACCATGGGAAGGGGAGCCCCA | Addition of ½ attB sites to the coding sequence of OsAPSE. | *n.a.* | This study |
| L367 | Rv | AGAAAGCTGGGTGACATGTAATTTCAAAC |  |  |  |
| Evd002 | Fw | GGGGACAAGTTTGTACAAAAAAGCAGGCT | Completion of attB sites | *n.a.* | (Van Hove et al., 2011) |
| Evd004 | Rv | GGGGACCACTTGTACAAGAAAGCTGGGT |  |  |  |
| Evd386 | Fw | GTAAAACGACGGCCAG | Amplification of the pK7FWG2 backbone | *n.a.* | (Van Hove et al., 2011) |
| Evd387 | Rv | CAGGAAACAGCTATGAC |  |  |  |
| Abbreviations: n.a. (not applicable) | | | | | |

**References**

Van Hove, J., Fouquaert, E., Smith, D. F., Proost, P., and Van Damme, E. J. M. (2011). Lectin activity of the nucleocytoplasmic EUL protein from Arabidopsis thaliana. *Biochem. Biophys. Res. Commun.* 414, 101–105. doi: 10.1016/j.bbrc.2011.09.031

Xu, H., Bao, J.-D., Dai, J.-S., Li, Y., and Zhu, Y. (2015). Genome-Wide Identification of New Reference Genes for qRT-PCR Normalization under High Temperature Stress in Rice Endosperm. *PLOS ONE* 10, e0142015. doi: 10.1371/journal.pone.0142015

Zhan, L.-P., Peng, D.-L., Wang, X.-L., Kong, L.-A., Peng, H., Liu, S.-M., et al. (2018). Priming effect of root-applied silicon on the enhancement of induced resistance to the root-knot nematode Meloidogyne graminicola in rice. *BMC Plant Biol.* 18, 50. doi: 10.1186/s12870-018-1266-9
