# Supplementary material for: OsAPSE modulates non-covalent interactions between arabinogalactan protein O-glycans and pectin in rice cell walls
Source: Front Plant Sci. 2025 May 22;16:1588802. doi: 10.3389/fpls.2025.1588802 (PMC12137362; doi:10.3389/fpls.2025.1588802)
Supplement: Supplementary file 7 [file Table7.docx]

**Supplementary File S7 – Cloning and sequencing of the pALiCE02::GH27_OsAPSE construct**.

**Section 1/4**: Cloning by restriction and ligation

| **Table S7.1 – Quantity and purity of the insert and expression plasmids, determined through UV-spectrophotometry (Nanodrop2000).** | | | |
| --- | --- | --- | --- |
|  | **Concentration (ng/µL)** | **A260/280** | **A260/230** |
| pGH27_OsAPSE | 601.0 | 1.96 | 2.29 |
| pALiCE02 | 356.9 | 1.91 | 2.24 |

| **Table S7.2 – Restriction mixtures for the insert and expression plasmids.** The restriction mixtures were incubated at 37°C for 1h and inactivated at 80°C for 20 minutes. | | |
| --- | --- | --- |
|  | **pGH27_OsAPSE**  c = 601 ng/µL | **pALiCE02**  c = 356.9 ng/µL |
| Bidest  rCutSmart buffer  5 µg DNA  *Kpn*I (2.5 U)  *Nco*I (2.5 U) | 36.2 µL  5 µL  8.3 µL  0.25 µL  0.25 µL | 30.5 µL  5 µL  14 µL  0.25 µL  0.25 µL |
| Total volume | 50 µL | 50 µL |

| **Table S7.3 – Quantity and purity of the digested insert and expression plasmids, determined through UV-spectrophotometry (Nanodrop2000).** | | | |
| --- | --- | --- | --- |
|  | **Concentration (ng/µL)** | **A260/280** | **A260/230** |
| pGH27_OsAPSE | 185.1 | 1.81 | 1.16 |
| pALiCE02 | 181.5 | 1.77 | 1.10 |

| **Table S7.4 – Composition of the ligation mixture in a 3/1 insert (pGH27_OsAPSE) to vector (pALiCE02) ratio.** The ligation mixtures were incubated at room temperature for 30 minutes. | |
| --- | --- |
| **Compound** | **Volume** |
| 10X Ligase buffer  Insert (pGH27_OsAPSE)  Vector (pALiCE02)  Bidest  100 mM dithiothreitol  T4 DNA ligase | 2 µL  6.6 µL  2.2 µL  6.2 µL  1 µL  2 µL |
| Total volume | 20 µL |

**Section 2/4**: Transformation and selection of recombinant *E. coli* TOP10 cells

| **Table S7.5 – PCR amplification of the GH27_OsAPSE fragment from putatively transformed *E. coli* TOP10 cells.** A total of 10 colonies were picked up and resuspended in 10 µL bidest and then submitted to the PCR program as indicated in this table. Primer oligonucleotide sequences are shown in Supplementary File S1. | |
| --- | --- |
| **Master mix** | **PCR program** |
| \| **Compound** \| **Volume** \| \| --- \| --- \| \| H_2_O  10X extra buffer  10 mM dNTPs  10 µM Fw (P154)  10 µM Rv (P155)  Taq polymerase \| 8.3 µL  2.5 µL  2.0 µL  1.0 µL  1.0 µL  0.2 µL \| | \| **Step** \| \| **Temp.** \| **Time** \| \| --- \| --- \| --- \| --- \| \| Initial denaturation \| \| 95°C \| 5 min \| \| 35x \| Denaturation \| 95°C \| 30 s \| \| Annealing \| 53°C \| 30 s \| \| Elongation \| 72°C \| 60 s \| \| Final elongation \| \| 72°C \| 5 min \| \| Infinite hold \| \| 12°C \| ∞ \| |


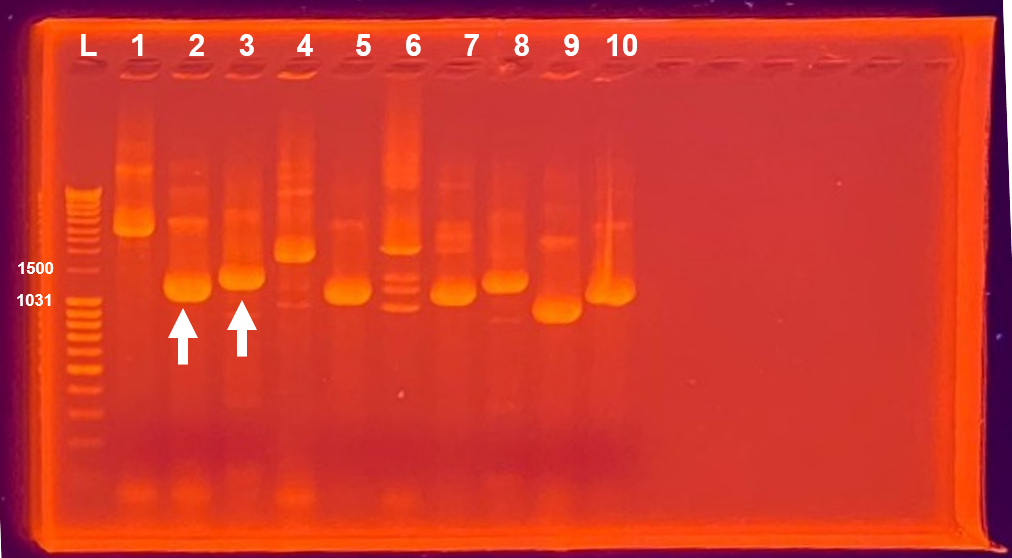


**Figure S7.1 – Ethidium bromide stained 1.5% agarose (w/v) gels loaded with PCR products.** The target amplicon size is 1245 bp. Colonies 2 and 3 were selected and grown overnight in LB + carbenicillin (80 µg/mL) at 37°C (220 rpm) and submitted to plasmid isolation and sent for sequencing with vector-specific primers (P154/155).

| **Table S7.6 – Quantity and purity of the isolated recombinant expression plasmids, determined through UV-spectrophotometry (Nanodrop2000).** | | | |
| --- | --- | --- | --- |
|  | **Concentration (ng/µL)** | **A260/280** | **A260/230** |
| Colony 2 | 287.2 | 1.95 | 2.63 |
| Colony 3 | 203.9 | 2.01 | 2.79 |

**Section 3/4**: Sequencing of pALiCE02::GH27_OsAPSE against the designed construct.

CLUSTAL O(1.2.4) multiple sequence alignment

sequencing TTTCTTACATCTATGCGGCTGCCATGGGCTGGAACTCCTACGACTCTTTCTCTTGGATCG 60

designed -----------------------ATGGGCTGGAACTCCTACGACTCTTTCTCTTGGATCG 37

*************************************

sequencing TGGACGAGAACACCTACATGCAGAACGCTGAGATTCTCGCCGAGAAGCTTTTGCCACATG 120

designed TGGACGAGAACACCTACATGCAGAACGCTGAGATTCTCGCCGAGAAGCTTTTGCCACATG 97

************************************************************

sequencing GATACGAGTTCGCCGTGATCGATTACCTCTGGTACAGAAAGTACGTGCACGGCGCTTACA 180

designed GATACGAGTTCGCCGTGATCGATTACCTCTGGTACAGAAAGTACGTGCACGGCGCTTACA 157

************************************************************

sequencing CTGACTCCTACGGATTCGATAACATCGATGAGTGGGGCCGTCCATTTCCAGATCTTCAAA 240

designed CTGACTCCTACGGATTCGATAACATCGATGAGTGGGGCCGTCCATTTCCAGATCTTCAAA 217

************************************************************

sequencing GGTTCCCCAGCTCCAGGATCGATAAGGGATTCTCTCAGCTCGCTAACAAGGTGCACGGAA 300

designed GGTTCCCCAGCTCCAGGATCGATAAGGGATTCTCTCAGCTCGCTAACAAGGTGCACGGAA 277

************************************************************

sequencing TGGGACTCAAGTTCGGAATCCACCTCATGAAGGGCATTTCTCTCCAGGCTGTGAACGGCA 360

designed TGGGACTCAAGTTCGGAATCCACCTCATGAAGGGCATTTCTCTCCAGGCTGTGAACGGCA 337

************************************************************

sequencing ACACTCCAATCCTCGATATCAAGACCGGAAAGCCCTACGTTGAGGATGGAAGGCAATGGA 420

designed ACACTCCAATCCTCGATATCAAGACCGGAAAGCCCTACGTTGAGGATGGAAGGCAATGGA 397

************************************************************

sequencing CTGCTCGTGATATTGGACTCACTCACAGGACTTGTGCTTGGATGCCTCACGGATTCATGT 480

designed CTGCTCGTGATATTGGACTCACTCACAGGACTTGTGCTTGGATGCCTCACGGATTCATGT 457

************************************************************

sequencing CCGTGAACACTGATATCGGAGCTGGCAAGGCTTTCCTCAGATCTCTTTACCAGCAGTACG 540

designed CCGTGAACACTGATATCGGAGCTGGCAAGGCTTTCCTCAGATCTCTTTACCAGCAGTACG 517

************************************************************

sequencing CTGATTGGGGCGTCGACTTTGTTAAGGTGGACTGCATCTTCGGCACCGACTACTCCCCAA 600

designed CTGATTGGGGCGTCGACTTTGTTAAGGTGGACTGCATCTTCGGCACCGACTACTCCCCAA 577

************************************************************

sequencing AAGAGATTATTACCATCTCCGAGCTGCTCGCCGAGCTTGATAGGCCAATTATCCTCTCCA 660

designed AAGAGATTATTACCATCTCCGAGCTGCTCGCCGAGCTTGATAGGCCAATTATCCTCTCCA 637

************************************************************

sequencing TTTCTCCCGGAACTGAGGTGACACCAGCTCTCGCTAAGAACATCTCTCAGCACGTGAACA 720

designed TTTCTCCCGGAACTGAGGTGACACCAGCTCTCGCTAAGAACATCTCTCAGCACGTGAACA 697

************************************************************

sequencing TGTACAGGATCACTGGCGACGATTGGGACAACTGGAAGGATGTGTCATCCCACTTCGACG 780

designed TGTACAGGATCACTGGCGACGATTGGGACAACTGGAAGGATGTGTCATCCCACTTCGACG 757

************************************************************

sequencing TGTCCTCTTCATTTGCTGCTGCTAACAAGATCGGAGCTATCGGACTTAGAGGAAGGTCTT 840

designed TGTCCTCTTCATTTGCTGCTGCTAACAAGATCGGAGCTATCGGACTTAGAGGAAGGTCTT 817

************************************************************

sequencing GGCCAGATCTCGATATGCTTCCATTCGGCTGGCTTACTAACGCTGGTGTTAATCAGGGAC 900

designed GGCCAGATCTCGATATGCTTCCATTCGGCTGGCTTACTAACGCTGGTGTTAATCAGGGAC 877

************************************************************

sequencing CACATAGGCAGTGCGAGCTTACTTCTGATGAGCAGAGGACTCAGATCGCCCTTTGGTCTA 960

designed CACATAGGCAGTGCGAGCTTACTTCTGATGAGCAGAGGACTCAGATCGCCCTTTGGTCTA 937

************************************************************

sequencing TGGCTAAGAGCCCACTTATGTACCGGTGGTGATCTTAGGCACCTCGATAACGACACCCTC 1020

designed TGGCTAAGAGCCCACTTATGTACCGGTGGTGATCTTAGGCACCTCGATAACGACACCCTC 996

************************************************************

sequencing AGCATTATTACTAACCCCACTCTCCTCAAGATCAACCACTCTCATCATCACCATCACCAC 1080

designed AGCATTATTACTAACCCCACTCTCCTCAAGATCAACCACTCTCATCATCACCATCACCAC 1056

************************************************************

sequencing TGATGAGGTACCAAGCTCTTCCGGGTTTGGTTTGGACC 1118

designed TGATGAGGTACC-------------------------- 1068

************

**Section 4/4**: Nucleobond pALiCE02::GH27_OsAPSE plasmid isolation and purification

| **Table S7.6 – Quantity and purity of the isolated recombinant expression plasmids, purified with the Nucleobond protocol and determined through UV-spectrophotometry (Nanodrop2000).** After the measurement, the recombinant pALiCE02::GH27_OsAPSE expression plasmid was diluted to a working concentration of 1000 ng/µL and stored at -20°C until application. | | | |
| --- | --- | --- | --- |
|  | **Concentration (ng/µL)** | **A260/280** | **A260/230** |
| pALiCE02::GH27_OsAPSE | 1167 | 1.90 | 2.11 |


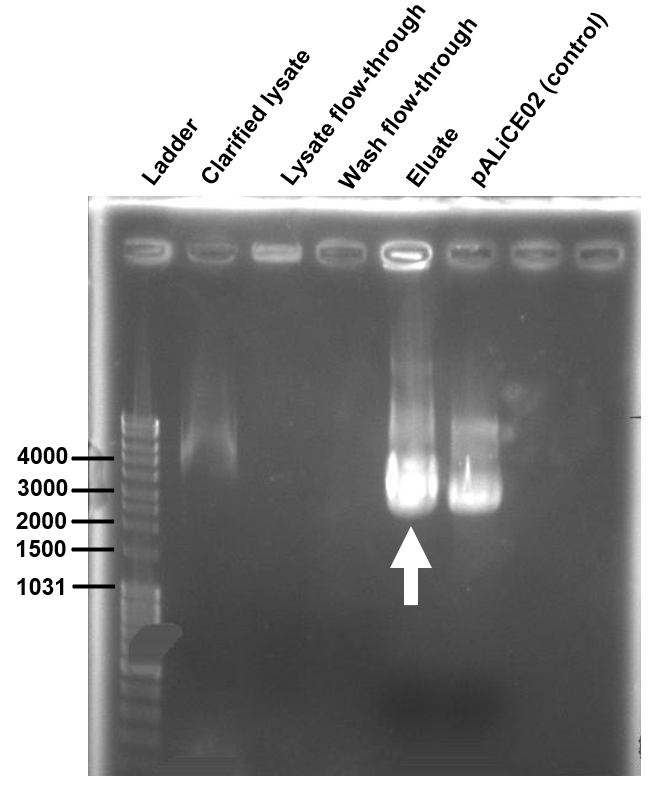


**Figure S7.2 – Purity analysis of the obtained fractions during isolation and purification of the pALiCE02::GH27_OsAPSE plasmid following the Nucleobond protocol.** The empty-vector pALiCE02 plasmid has a size of 3088 bp while the pALiCE02::GH27_OsAPSE has a size of 3310 bp.
