# Supplementary material for: OsAPSE modulates non-covalent interactions between arabinogalactan protein O-glycans and pectin in rice cell walls
Source: Front Plant Sci. 2025 May 22;16:1588802. doi: 10.3389/fpls.2025.1588802 (PMC12137362; doi:10.3389/fpls.2025.1588802)
Supplement: Supplementary file 9 [file Table9.docx]

**Supplementary File S9 – In silico prediction of localization and PTMs for OsAPSE.**

**Section 1**: *In silico* tools to study the presence of localization signals and post-translational modifications. Image: output of SignalP v6.0.

|  | **Signal peptide** | **Other transfer signals** | ***N*-glycosylation** | ***O*-glycosylation** | **Disulfide bridges** | **Membrane association** |
| --- | --- | --- | --- | --- | --- | --- |
| **Online tool** | SignalP v6.0 | TargetP v2.0  NucPred | NetNGlyc v1 | NetOGlyc v4 | DiANNA v1.1 | DeepLoc v2.1 |
| **Result** | Signal peptide and cleavage site present | Absent | 4 confident sites for *N*-glycosylation | 5 confident sites for *O*-glycosylation | 3 confident disulfide bridges | Not associated to membranes |
| **Confidence** | 0.9997 | 0.9998 (TargetP v2.0)  0.24 (NucPred) | N269 (NIS): 0.6388  N372 (NDT): 0.6526  N380 (NPT): 0.6047 (contains proline!)  N466 (NIS): 0.3023 | T176: 0.6172  T186: 0.5769  T515: 0.7531  S516: 0.7874  S517: 0.8525 | C340-505: 0.9997  C435-527: 0.9657  C456-551: 0.9928 | 0.7350 |
| **Source** | (Teufel et al., 2022) | (Brameier et al., 2007; Almagro Armenteros et al., 2019) | (Gupta and Brunak, 2002) | (Steentoft et al., 2013) | (Ferre and Clote, 2005) | (Ødum et al., 2024) |
| Notes: *including nuclear localization signals, mitochondrial, chloroplast and thylakoid luminal transfer peptides | | | | | | |


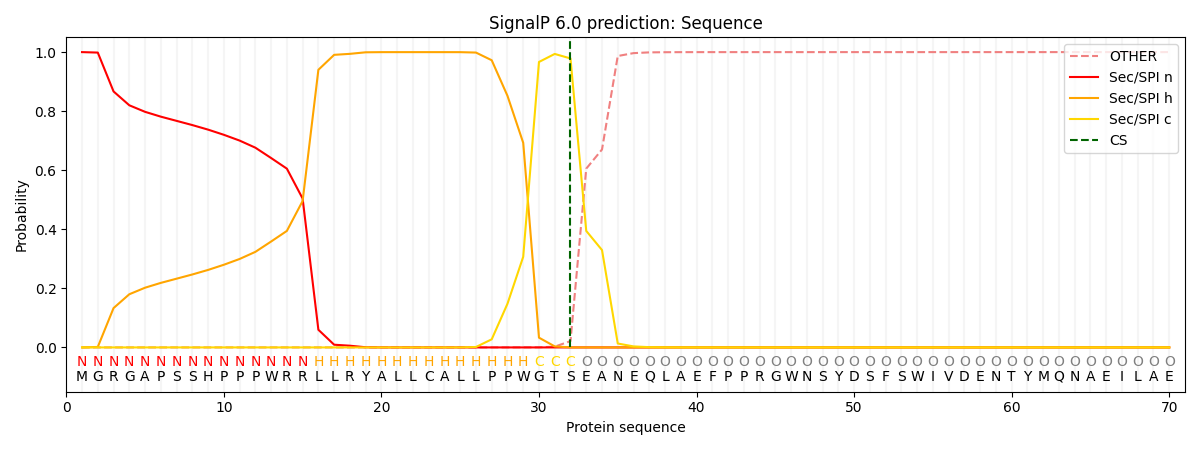


**Section 2**: Prediction of post-translational modifications, modelled on the structure of the GH27 domain of OsAPSE. Three putative sequon (NXT/S) sites for N-glycosylation (indicated in **blue**) were predicted and are likely to present N-glycans that are oriented towards the protein surface. Cysteines putatively involved in disulfide bridge formation are shown in **red**. The GH27 domain of OsAPSE most likely contains 1 disulfide bridge between C187 and C227 (inter-atomical distance of 2.028 Å) with favorable dihedral angle.


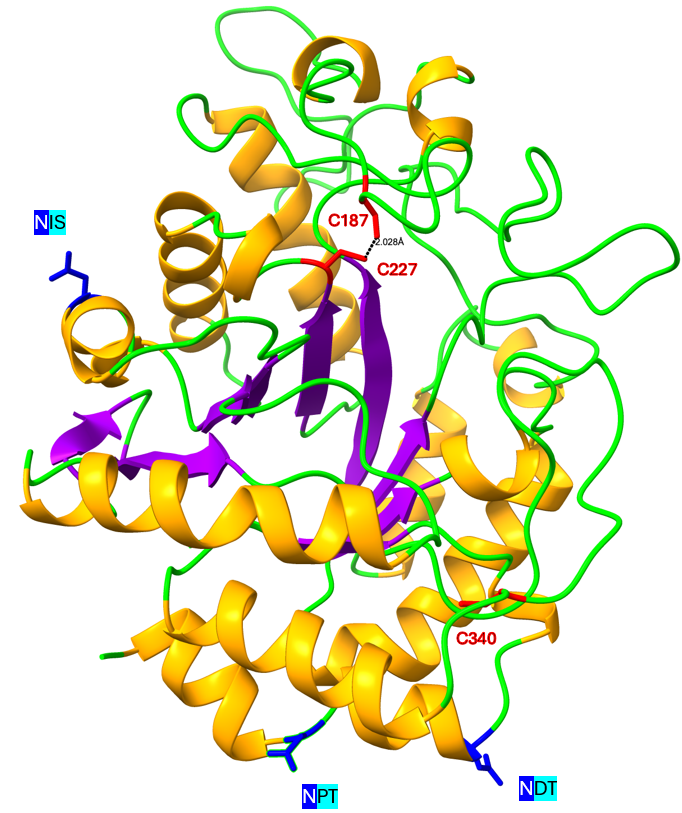


MGRGAPSSHPPPWRRLLRYALLCALLPPWGTSEANEQLAEFPPRGWNSYDSFSWIVDENTYMQNAEILAEKLLPHGYEFAVIDYLWYRKYVHGAYTDSYGFDNIDEWGRPFPDLQRFPSSRIDKGFSQLANKVHGMGLKFGIHLMKGISLQAVNGNTPILDIKTGKPYVEDGRQWTARDIGLTHRTCAWMPHGFMSVNTDIGAGKAFLRSLYQQYADWGVDFVKV**D**CIFGTDYSPKEIITISELLAELDRPIILSISPGTEVTPALAKNISQHVNMYRITG**D**DWDNWKDVSSHFDVSSSFAAANKIGAIGLRGRSWPDLDMLPFGWLTNAGVNQGPHRQCELTSDEQRTQIALWSMAKSPLMYGGDLRHLDNDTLSIITNPTLLKINHYSINNMEFHHVHSERTSKEDKHSSRFISEDLVHVPKIDGVSLGLTACSDDKANGWYMFSQHGKSDHICRNYGMQDDKNISFCLGKTIPLLTSDDIIVHNEEYQTKFHLANMDSDDACLDASGSQRRTSSDSKFPMFSRCRWHAMQMWELNEKGNLISSYSRLCATVESNNKGVVTTGAVARAWIATGSKGEIYLAFFNLDSMSRKITARISDLEKVLGSTFIRKDTCSCTEVWSGRNFGRVEEEISAVVKSHGSMVFEITC

**Section 3**: Prediction of subcellular localization for OsAPSE based on in silico tools

| **Prediction tool** | **MultiLoc 2** | **Plant-mPLoc** | **CELLO v2.5** | **DeepLoc v1** | **MuLocDeep** | **DeepLoc v2.1** |
| --- | --- | --- | --- | --- | --- | --- |
| Extracellular space  Endoplasmic reticulum  Plasma membrane  Vacuoles  Mitochondria  Golgi apparatus  Peroxisomes  Lysosomes  Cytosol  Nucleus  Chloroplasts  Cytoskeleton  Cell wall | **0.19**  **0.19**  0.12  0.12  0.11  0.09  0.08  *n.a.*  0.06  0.02  0.01  *n.a.*  *n.a.* | *n.a.*  *n.a.*  *n.a.*  *n.a.*  *n.a.*  *n.a.*  *n.a.*  *n.a.*  *n.a.*  *n.a.*  *n.a.*  *n.a.*  **1** | **1.147**  0.128  **1.292**  0.101  0.492  0.030  0.187  0.490  0.570  0.278  0.260  0.026  *n.a.* | **0.969**  0.018  0.001  0  0  0  0  0.018  0  0  0  *n.a.*  *n.a.* | **0.1572**  0.0910  0.0300  *n.a.*  0.0016  0.0183  0.0009  0.0067  0.0465  0.0057  0.0024  *n.a.*  *n.a.* | 0.4865  **0.5377**  0.3146  **0.5560**  0.0374  0.2630  0.0077  **0.5560**  0.1261  0.1300  0.0577  *n.a.*  *n.a.* |
| **Conclusion** | Cell surface | Cell surface | Cell surface | Cell surface | Cell surface | Vacuoles |
| **Source** | (Blum et al., 2009) | (Chou and Shen, 2010) | (Yu et al., 2014) | (Almagro Armenteros et al., 2019) | (Jiang et al., 2023) | (Ødum et al., 2024) |

**References**

Almagro Armenteros, J. J., Tsirigos, K. D., Sønderby, C. K., Petersen, T. N., Winther, O., Brunak, S., et al. (2019). SignalP 5.0 improves signal peptide predictions using deep neural networks. *Nat. Biotechnol.* 37, 420–423. doi: 10.1038/s41587-019-0036-z

Blum, T., Briesemeister, S., and Kohlbacher, O. (2009). MultiLoc2: integrating phylogeny and Gene Ontology terms improves subcellular protein localization prediction. *BMC Bioinformatics* 10, 274. doi: 10.1186/1471-2105-10-274

Brameier, M., Krings, A., and MacCallum, R. M. (2007). NucPred—Predicting nuclear localization of proteins. *Bioinformatics* 23, 1159–1160. doi: 10.1093/bioinformatics/btm066

Chou, K.-C., and Shen, H.-B. (2010). Plant-mPLoc: A Top-Down Strategy to Augment the Power for Predicting Plant Protein Subcellular Localization. *PLoS ONE* 5, e11335. doi: 10.1371/journal.pone.0011335

Ferre, F., and Clote, P. (2005). DiANNA: a web server for disulfide connectivity prediction. *Nucleic Acids Res.* 33, W230–W232. doi: 10.1093/nar/gki412

Gupta, R., and Brunak, S. (2002). Prediction of glycosylation across the human proteome and the correlation to protein function. *Pac. Symp. Biocomput.* 7, 310–322.

Jiang, Y., Jiang, L., Akhil, C. S., Wang, D., Zhang, Z., Zhang, W., et al. (2023). MULocDeep web service for protein localization prediction and visualization at subcellular and suborganellar levels. *Nucleic Acids Res.* 51, W343–W349. doi: 10.1093/nar/gkad374

Ødum, M. T., Teufel, F., Thumuluri, V., Almagro Armenteros, J. J., Johansen, A. R., Winther, O., et al. (2024). DeepLoc 2.1: multi-label membrane protein type prediction using protein language models. *Nucleic Acids Res.* 52, W215–W220. doi: 10.1093/nar/gkae237

Steentoft, C., Vakhrushev, S. Y., Joshi, H. J., Kong, Y., Vester-Christensen, M. B., Schjoldager, K. T.-B. G., et al. (2013). Precision mapping of the human O-GalNAc glycoproteome through SimpleCell technology. *EMBO J.* 32, 1478–1488. doi: 10.1038/emboj.2013.79

Teufel, F., Almagro Armenteros, J. J., Johansen, A. R., Gíslason, M. H., Pihl, S. I., Tsirigos, K. D., et al. (2022). SignalP 6.0 predicts all five types of signal peptides using protein language models. *Nat. Biotechnol.* 40, 1023–1025. doi: 10.1038/s41587-021-01156-3

Yu, C.-S., Cheng, C.-W., Su, W.-C., Chang, K.-C., Huang, S.-W., Hwang, J.-K., et al. (2014). CELLO2GO: A Web Server for Protein subCELlular LOcalization Prediction with Functional Gene Ontology Annotation. *PLoS ONE* 9, e99368. doi: 10.1371/journal.pone.0099368
