# Supplementary material for: OsAPSE modulates non-covalent interactions between arabinogalactan protein O-glycans and pectin in rice cell walls
Source: Front Plant Sci. 2025 May 22;16:1588802. doi: 10.3389/fpls.2025.1588802 (PMC12137362; doi:10.3389/fpls.2025.1588802)
Supplement: Supplementary file 10 [file Table10.docx]

**Supplementary File S10 – Selection and screening of transgenic plants**

Mutant *osapse* rice seeds and overexpressing pUBI::OsAPSE rice seeds from the F_2_ generation were obtained and multiplied as explained earlier. The CTAB protocol is widely used for the extraction of nucleic acids (De Wever et al., 2021) and resulted in high concentrations of pure gDNA, reaching average concentrations of 1586.0 ± 1005.1 ng/µL. Furthermore, the gDNA was of high purity, as assessed by the A260/280 and A260/230 ratios equalling 2.049 ± 0.049 and 2.259 ± 0.122 on average (**Table S10.1**).

| **Table S10.1 – Quantity and quality of genomic DNA from transgenic/mutant/WT rice seedlings** | | | | |
| --- | --- | --- | --- | --- |
| **Line** | **DNA concentration (ng/µL)** | **A260/280 ratio** | **A260/230 ratio** | **Amplification of Hyg^R^** |
| OE29.1a  OE29.1b | 1511.9  1176.6 | 2.07  2.06 | 2.35  2.40 | OK |
| OE29.2a  OE29.2b | 857.6  2314.0 | 2.12  2.00 | 2.43  2.22 | OK |
| OE29.3a  OE29.3b | 3820.3  2316.7 | 1.99  2.03 | 2.09  2.15 | OK |
| OE29.4a  OE29.4b | 3488.3  2753.0 | 2.04  2.06 | 2.16  2.19 | OK |
| OE26.1a  OE26.1b | 1000.5  1086.8 | 2.04  2.06 | 2.46  2.37 | OK |
| OE26.2a  OE26.2b | 1800.7  1594.8 | 2.07  2.11 | 2.13  2.17 | OK |
| OE26.3a  OE26.3b | 1525.6  1606.3 | 2.04  2.10 | 2.07  2.19 | OK |
| OE26.4a  OE26.4b | 1101.6  1151.9 | 2.10  2.11 | 2.25  2.17 | OK |
| OE26.5a  OE26.5b | 987.0  539.0 | 2.12  2.14 | 2.13  2.25 | OK |
| OE28.1a  OE28.1b | 1101.1  1211.2 | 2.06  2.11 | 2.20  2.26 | OK |
| OE28.2a  OE28.2b | 3509.3  3588.1 | 2.04  2.06 | 2.21  2.20 | OK |
| OE28.3a  OE28.3b | 1018.6  1533.0 | 2.12  2.09 | 2.31  2.21 | OK |
| OE28.4a  OE28.4b | Not sampled | Not sampled | Not sampled | *n.a.* |
| OE28.5a  OE28.5b | 535.2  353.4 | 2.07  2.01 | 2.58  2.64 | OK |
| KO4.1a  KO4.1b | 1511.2  569.9 | 2.05  2.06 | 2.27  2.21 | *n.a.*  *n.a.* |
| KO4.2a  KO4.2b | 1564.2  942.7 | 2.05  2.03 | 2.29  2.31 | *n.a.*  *n.a.* |
| KO4.3a  KO4.3b | 598.3  1065.8 | 2.08  2.03 | 2.33  2.26 | *n.a.*  *n.a.* |
| KO4.4a  KO4.4b | 1020.6  1435.3 | 2.06  2.05 | 2.33  2.23 | *n.a.*  *n.a.* |
| KO4.5a  KO4.5b | 1464.9  653.6 | 2.04  2.06 | 2.30  2.38 | *n.a.*  *n.a.* |
| KO5.5a  KO5.5b | 892.9  1271.9 | 2.09  2.06 | 2.35  2.29 | *n.a.*  *n.a.* |
| KO5.3a  KO5.3b | 1856.4  626.2 | 2.03  2.09 | 2.29  2.35 | *n.a.*  *n.a.* |
| KO5.1a  KO5.1b | 1189.8  4033.2 | 2.06  1.97 | 2.28  2.10 | *n.a.*  *n.a.* |
| KO5.2a  KO5.2b | 4543.4  1820.0 | 1.83  2.00 | 1.93  2.18 | *n.a.*  *n.a.* |
| KO9.1a  KO9.1b | 1944.8  514.5 | 2.02  1.98 | 2.22  2.27 | *n.a.*  *n.a.* |
| KO9.2a  KO9.2b | 606.1  188.9 | 2.08  1.94 | 2.29  2.42 | *n.a.*  *n.a.* |
| KO9.3a  KO9.3b | 1141.1  3232.1 | 2.07  2.04 | 2.30  2.15 | *n.a.*  *n.a.* |
| KO9.4a  KO9.4b | 743.9  1264.7 | 2.04  2.02 | 2.21  2.30 | *n.a.*  *n.a.* |
| KO9.5a  KO9.5b | 854.8  1610.5 | 2.05  2.03 | 2.28  2.38 | *n.a.*  *n.a.* |
| WT1  WT2  WT3 | 2468.3  3080.3  2211.2 | 1.99  2.06  2.04 | 1.99  2.23  2.24 | Not amplified |
| **Average** | 1586.0 ± 1005.1 | 2.049 ± 0.049 | 2.259 ± 0.122 | *n.a.* |
| Abbreviations: *n.a.* (not applicable). | | | | |

As a matter of quality control, a fragment of the hygromycin resistance gene was amplified from the OSAPSE gDNA. This fragment could not be amplified from WT gDNA (**Figure S10.1**).

**Figure S10.1 – UV detection of PCR amplified fragments of the hygromycin resistance gene after ethidium bromide staining in 3% agarose gels in Tris-Acetate-EDTA buffer.** Reference sizes from the molecular ladder are highlighted.


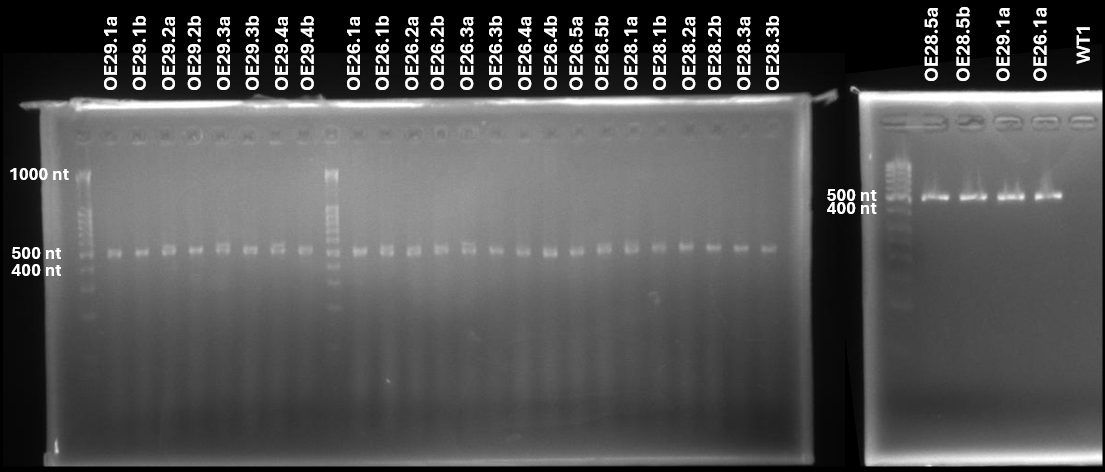


For the mutant *osapse* gDNA, the sequence complementary to the guide RNA was amplified, purified and sequenced. The obtained sequencing chromatograms were analysed and compared to the WT (**Table S10.2**). Mutant knock-out lines *osapse4* and *osapse5* display identical mutations, while *osapse9* shows unique mutations (**Table S10.3,** summary in **Table S10.4**). In *oaspse4* and *osapse5*, the combined effect of a cytosine to adenine substitution (*i.e.* leading to a P43H point mutation) and deletion of two nucleotides within the first CRISPR site cause an important frameshift mutation, leading to a premature stopcodon after 147 nucleotides. Additionally, a deletion of 4 nucleotides in the second CRISPR site cause an additional frameshift. In *osapse9*, a large deletion causes the loss of 233 nucleotides, thereby considerably mutilating the resulting polypeptide.

| **Table S10.2 – Chromatograms after sequencing of the 2 CRISPR sites in the WT and KO plants** | | |
| --- | --- | --- |
| **Line** | **Site 1** | **Site 2** |
| WT | 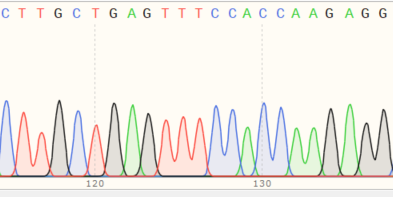 | 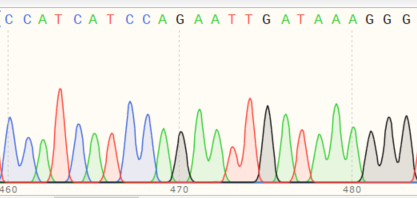 |
| KO4 | 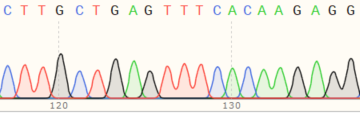 | 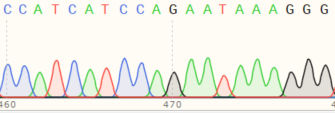 |
| KO5 | 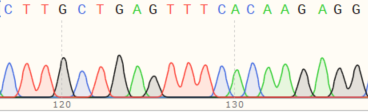 | 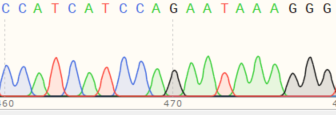 |
| KO9 | 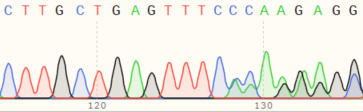 | *n.a.* |

| **Table S10.3 – Effect of mutations in the different KO mutants** | |
| --- | --- |
| **Partial OsAPSE native nucleotide and amino acid sequence (WT)** | ***osapse4; osapse5***  **site1: C 🡺 A substitution + deletion (AC): frameshift mutation**  **site2: deletion (TTGA): frameshift mutation** |
| **ATG**GGAAGGGGAGCCCCATCCTCCCATCCGCCGCCATGGCGCCGCCTCCTCCGCTACGCC  CTCCTCTGCGCCCTCCTCCCCCCCTGGGGCACTTCCGAGGCTAATGAACAACTTGCTGAG  TTTCCACCAAGAGGCTGGAATTCCTATGATTCCTTTTCATGGATAGTTGATGAAAATACA  TACATGCAAAATGCGGAGATCTTGGCAGAAAAATTGCTCCCACATGGATATGAGTTTGCA  GTTATTGATTACCTCTGGTACCGAAAGTATGTTCATGGGGCATACACAGATTCATATGGA  TTTGATAACATTGATGAGTGGGGTCGGCCATTTCCTGATCTTCAAAGATTTCCATCATCC  AGAATTGATAAAGGGTTCAGTCAACTTGCGAACAAGGTGCATGGAATGGGCTTGAAATTC  GGAATCCATTTAATGAAAGGGATAAGTTTACAGGCTGTGAATGGAAACACACCCATATTG  GACATTAAAACGGGAAAACCCTACGTAGAGGATGGCCGGCAATGGACAGCTCGTGATATA  ...  MGRGAPSSHPPPWRRLLRYALLCALLPPWGTSEANEQLAEFPPRGWNSYDSFSWIVDENT  YMQNAEILAEKLLPHGYEFAVIDYLWYRKYVHGAYTDSYGFDNIDEWGRPFPDLQRFPSS  RIDKGFSQLANKVHGMGLKFGIHLMKGISLQAVNGNTPILDIKTGKPYVEDGRQWTARDI  ... | **ATG**GGAAGGGGAGCCCCATCCTCCCATCCGCCGCCATGGCGCCGCCTCCTCCGCTACGCC  CTCCTCTGCGCCCTCCTCCCCCCCTGGGGCACTTCCGAGGCTAATGAACAACTTGCTGAG  TTTC**CAC**CAAGAGGCTGGAATTCCTATGATTCCTTTTCATGGATAGTTGATGAAAATACA  TACATGCAAAATGCGGAGATCTTGGCAGAAAAATTGCTCCCACATGGATATGAGTTTGCA  GTTATTGATTACCTCTGGTACCGAAAGTATGTTCATGGGGCATACACAGATTCATATGGA  TTTGATAACATTGATGAGTGGGGTCGGCCATTTCCTGATCTTCAAAGATTTCCATCATCC  AGAA**TTGA**TAAAGGGTTCAGTCAACTTGCGAACAAGGTGCATGGAATGGGCTTGAAATTC  GGAATCCATTTAATGAAAGGGATAAGTTTACAGGCTGTGAATGGAAACACACCCATATTG  GACATTAAAACGGGAAAACCCTACGTAGAGGATGGCCGGCAATGGACAGCTCGTGATATA  ...  **M**GRGAPSSHPPPWRRLLRYALLCALLPPWGTSEANEQLAEF**P**PRGWNSYDSFSWIVDENT  YMQNAEILAEKLLPHGYEFAVIDYLWYRKYVHGAYTDSYGFDNIDEWGRPFPDLQRFPSS  RIDKG**F**SQLANKVHGMGLKFGIHLMKGISLQAVNGNTPILDIKTGKPYVEDGRQWTARDI  ...  ⇓  **ATG**GGAAGGGGAGCCCCATCCTCCCATCCGCCGCCATGGCGCCGCCTCCTCCGCTACGCC  CTCCTCTGCGCCCTCCTCCCCCCCTGGGGCACTTCCGAGGCTAATGAACAACTTGCTGAG  TTTC**A**__CAAGAGGCTGGAATTCCTA**TGA^1^***TTCCTTTTC***ATG**GATAGT**TGATGA^2^***AAATAC*  *ATAC***ATG**CAAAATGCGGAGATCTTGGCAGAAAAATTGCTCCCACATGGATATGAGTTTGCA  GTTATTGATTACCTCTGGTACCGAAAGTATGTTCATGGGGCATACACAGATTCATATGGA  TTTGATAACATTGATGAGTGGGGTCGGCCATTTCCTGATCTTCAAAGATTTCCATCATCC  AGAA____TAAAGGGTTCAGTCAACTTGCGAACAAGGTGCATGGAATGGGCT**TGA^3^***AATTC*  *GGAATCCATTTA***ATG**AAAGGGATAAGTTTACAGGCTGTGAATGGAAACACACCCATATTG  GACATTAAAACGGGAAAACCCTACGTAGAGGATGGCCGGCAATGGACAGCTCGTGATATA  ...  **M**GRGAPSSHPPPWRRLLRYALLCALLPPWGTSEANEQLAEF**H**KRLEFL**-^1^***FLF***M**DS**--^2^***KYI*  **M**QNAEILAEKLLPHGYEFAVIDYLWYRKYVHGAYTDSYGFDNIDEWGRPFPDLQRFPSS  RIKG**S**VNLRTRCMEWA**-^3^***NSESI***M**KGISLQAVNGNTPILDIKTGKPYVEDGRQWTARDI  ... |

| **Table S10.3 – Effect of mutations in the different KO mutants (continued)** | |
| --- | --- |
| **Partial OsAPSE native nucleotide and amino acid sequence (WT)** | ***osapse9***  **site1-site2: deletion : frameshift mutation** |
| **ATG**GGAAGGGGAGCCCCATCCTCCCATCCGCCGCCATGGCGCCGCCTCCTCCGCTACGCC  CTCCTCTGCGCCCTCCTCCCCCCCTGGGGCACTTCCGAGGCTAATGAACAACTTGCTGAG  TTTCCACCAAGAGGCTGGAATTCCTATGATTCCTTTTCATGGATAGTTGATGAAAATACA  TACATGCAAAATGCGGAGATCTTGGCAGAAAAATTGCTCCCACATGGATATGAGTTTGCA  GTTATTGATTACCTCTGGTACCGAAAGTATGTTCATGGGGCATACACAGATTCATATGGA  TTTGATAACATTGATGAGTGGGGTCGGCCATTTCCTGATCTTCAAAGATTTCCATCATCC  AGAATTGATAAAGGGTTCAGTCAACTTGCGAACAAGGTGCATGGAATGGGCTTGAAATTC  GGAATCCATTTAATGAAAGGGATAAGTTTACAGGCTGTGAATGGAAACACACCCATATTG  GACATTAAAACGGGAAAACCCTACGTAGAGGATGGCCGGCAATGGACAGCTCGTGATATA  ...  MGRGAPSSHPPPWRRLLRYALLCALLPPWGTSEANEQLAEFPPRGWNSYDSFSWIVDENT  YMQNAEILAEKLLPHGYEFAVIDYLWYRKYVHGAYTDSYGFDNIDEWGRPFPDLQRFPSS  RIDKGFSQLANKVHGMGLKFGIHLMKGISLQAVNGNTPILDIKTGKPYVEDGRQWTARDI  ... | **ATG**GGAAGGGGAGCCCCATCCTCCCATCCGCCGCCATGGCGCCGCCTCCTCCGCTACGCC  CTCCTCTGCGCCCTCCTCCCCCCCTGGGGCACTTCCGAGGCTAATGAACAACTTGCTGAG  TTTC**CACCAAGAGGCTGGAATTCCTATGATTCCTTTTCATGGATAGTTGATGAAAATACA**  **TACATGCAAAATGCGGAGATCTTGGCAGAAAAATTGCTCCCACATGGATATGAGTTTGCA**  **GTTATTGATTACCTCTGGTACCGAAAGTATGTTCATGGGGCATACACAGATTCATATGGA**  **TTTGATAACATTGATGAGTGGGGTCGGCCATTTCCTGATCTTCAAAGATTTCCATCA**TCC  AGAATTGATAAAGGGTTCAGTCAACTTGCGAACAAGGTGCATGGAATGGGCTTGAAATTC  GGAATCCATTTAATGAAAGGGATAAGTTTACAGGCTGTGAATGGAAACACACCCATATTG  GACATTAAAACGGGAAAACCCTACGTAGAGGATGGCCGGCAATGGACAGCTCGTGATATA  ...  MGRGAPSSHPPPWRRLLRYALLCALLPPWGTSEANEQLAEFPPRGWNSYDSFSWIVDENT  YMQNAEILAEKLLPHGYEFAVIDYLWYRKYVHGAYTDSYGFDNIDEWGRPFPDLQRFPSS  RIDKGFSQLANKVHGMGLKFGIHLMKGISLQAVNGNTPILDIKTGKPYVEDGRQWTARDI  ...  ⇓  **ATG**GGAAGGGGAGCCCCATCCTCCCATCCGCCGCCATGGCGCCGCCTCCTCCGCTACGCC  CTCCTCTGCGCCCTCCTCCCCCCCTGGGGCACTTCCGAGGCTAATGAACAACTTGCTGAG  TTTCTCCAGAAT**TGATAA^1^**AGGGTTCAGTCAACTTGCGAACAAGGTGCATGGA**ATG**GGCTT  GAAATTCGGAATCCATTTAATGAAAGGGATAAGTTTACAGGCTGTGAATGGAAACACACC  CATATTGGACATTAAAACGGGAAAACCCTACGTAGAGGATGGCCGGCAATGGACAGCTCG  TGATATA  ...  MGRGAPSSHPPPWRRLLRYALLCALLPPWGTSEANEQLAEFLQN**--^1^***RVQSTCEQGAW***M**GL  KFGIHLMKGISLQAVNGNTPILDIKTGKPYVEDGRQWTARDI  ... |
| The left panel contains a part of the native coding sequence and amino acid sequence of OsAPSE. The right panel investigates the effect of the detected mutations on the amino acid sequence of the resulting mutant *osapse* proteins. Sites targeted for knock-out are highlighted in yellow and red. Start codons are indicated in green. Stop codons are indicated in red. Substitutions are marked in magenta, while deletions are marked in cyan. The combined effect of the different mutations is shown after the double arrow: the effect of deletions is highlighted with underlining (**__**). Stop codons due to frame shifts are indicated in red and numbered in superscript. Non-transcribed nucleotides and non-translated amino acids are shown in italics and *dotted underlining*. The first non-identical (KO compared to WT) amino acid is indicated in **bold**. In the reconstruction of the resulting *osapse* proteins, it is assumed that the next start codon after a premature stop codon is effective. | |

| **Table S10.4 – Overview of the mutations in the different KO lines** | | | |
| --- | --- | --- | --- |
| **Line** | **KO site 1** | **KO site 2** | **Result on putatively produced *osapse* protein** |
| *osapse4*  *osapse5* | Indel: P43H | Deletion | Frameshifts lead to multiple premature stop codons and new start codons. Multiple parts of the native OsAPSE protein are missing. |
| *osapse9* | Deletion of fragment between site 1 and site 2 | | Removal of large fragment (233 nt) leads to several premature stop codons and new start codons. Multiple large parts of the native OsAPSE protein are missing. |

From the central dogma of molecular biology we know how genetic information from the DNA is transcribed to mRNA and translated into proteins. Meanwhile it has been shown that there are several exceptions to the central dogma, and sometimes the central dogma is considered to oversimplify reality (Kozak, 1990). Normally, it is supposed that ribosomes bind to the 5’ cap of the mRNA, after which it scans in the 5’→3’ direction until the first start codon downstream of a Kozak sequence. Upon recognition of the start codon, translation is continued until a stop codon is encountered. Recognition of a stop codon leads to the hydrolysis of the polypeptide from the tRNA, but also the dissociation of the ribosomal subunits. Hence, it is normally assumed that separated ribosomal subunits are not immediately ready for reassembly on the same mRNA and that it is therefore not likely that a dissociated ribosome will bind to a downstream start codon after encountering a stop codon, since the ribosomes require recruitment at the 5’ cap of the mRNA. Translation is only seldomly initiated at internal start codons further downstream from the Kozak sequence. The occurrence of internal ribosome binding sites is very rare in eukaryotes and is typically observed in viruses (Arhab et al., 2020). However, it has been demonstrated several times before that post-termination ribosomes can reinitiate translation at downstream start codons in eukaryotic cells (Embree et al., 2022). This process enables to distinguish an open reading frame (ORF) upstream and downstream from the stop codon, and turns a presumably monocistronic ORF into a polycistronic ORF, leading to multiple truncated polypeptides originating from the same mRNA (Cohen et al., 2019). We adopted this train of thought and analysed the occurrence of start codons downstream from premature stop codons, which would give rise to alternative ORFs.

Eventually, different polypeptides with varying length and structure can arise from the observed mutations (**Table S10.4**).

| **Table S10.4 – Predicted structures of the polypeptides arising from KO mutations** | | |
| --- | --- | --- |
| **Line** | **Partial polypeptide** | **Modelled protein structure (AlphaFold) and pTM score** |
| Native | -- | pTM = 0.82  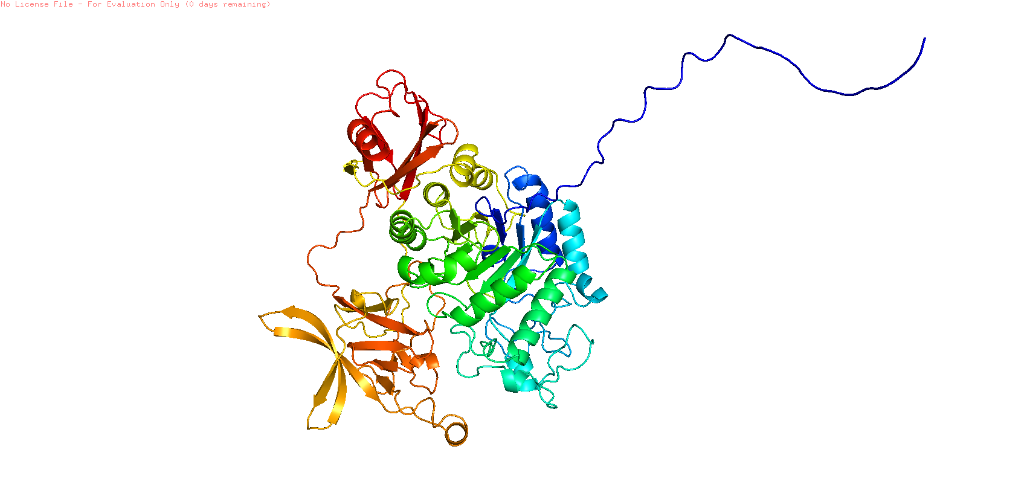 |
| *osapse4; osapse5* | **M**GRGAPSSHPPPWRRLLRYALLCAL  LPPWGTSEANEQLAEF**H**KRLEFL | pTM = 0.33  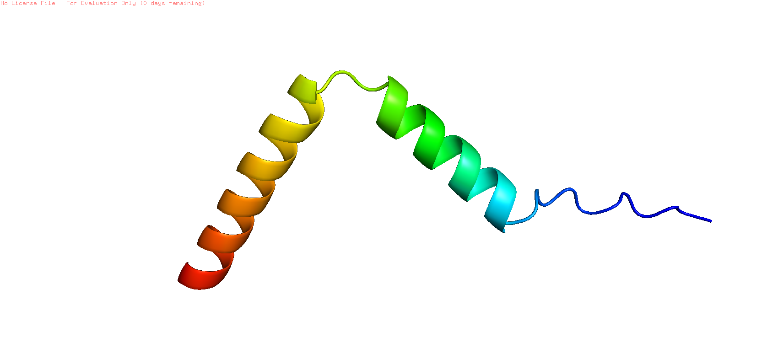 |
|  | **M**QNAEILAEKLLPHGYEFAVIDYLW  YRKYVHGAYTDSYGFDNIDEWGRPF  PDLQRFPSSRIKG**S**VNLRTRCMEWA | pTM = 0.62  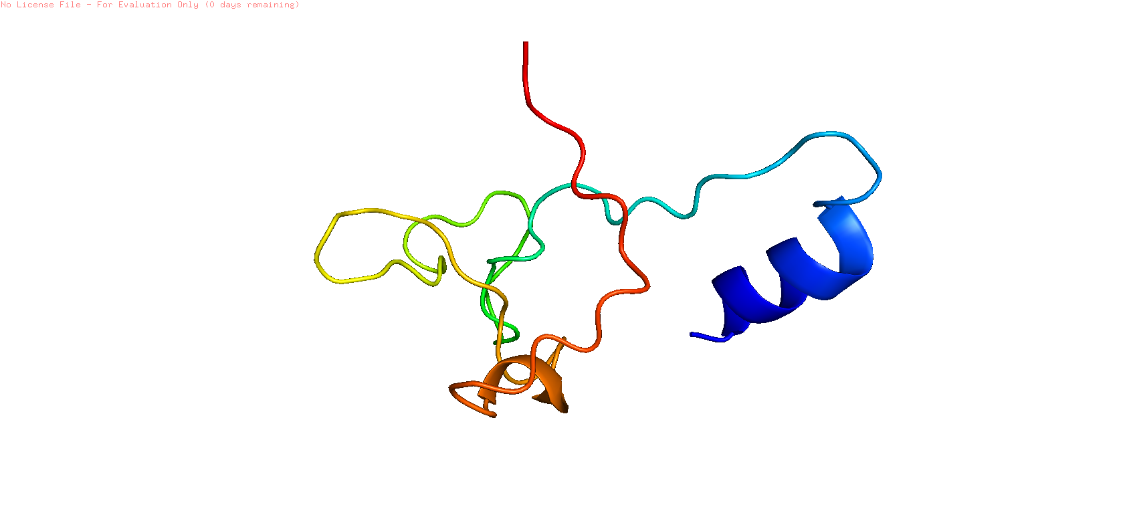 |
|  | **M**KGISLQAVNGNTPILDIKTGKPYVE  DGRQWTARDIGLTHRTCAWMPHGFMS  VNTDIGAGKAFLRSLYQQYADWGVDF  VKV**D**CIFGTDYSPKEIITISELLAEL  DRPIILSISPGTEVTPALAKNISQHV  NMYRITG**D**DWDNWKDVSSHFDVSSSF  AAANKIGAIGLRGRSWPDLDMLPFGW  LTNAGVNQGPHRQCELTSDEQRTQIA  LWSMAKSPLMYGGDLRHLDNDTLSII  TNPTLLKINHYSINNMEFHHVHSERT  SKEDKHSSRFISEDLVHVPKIDGVSL  GLTACSDDKANGWYMFSQHGKSDHIC  RNYGMQDDKNISFCLGKTIPLLTSDD  IIVHNEEYQTKFHLANMDSDDACLDA  SGSQRRTSSDSKFPMFSRCRWHAMQM  WELNEKGNLISSYSRLCATVESNNKG  VVTTGAVARAWIATGSKGEIYLAFFN  LDSMSRKITARISDLEKVLGSTFIRK  DTCSCTEVWSGRNFGRVEEEISAVVK  SHGSMVFEITC | pTM = 0.84  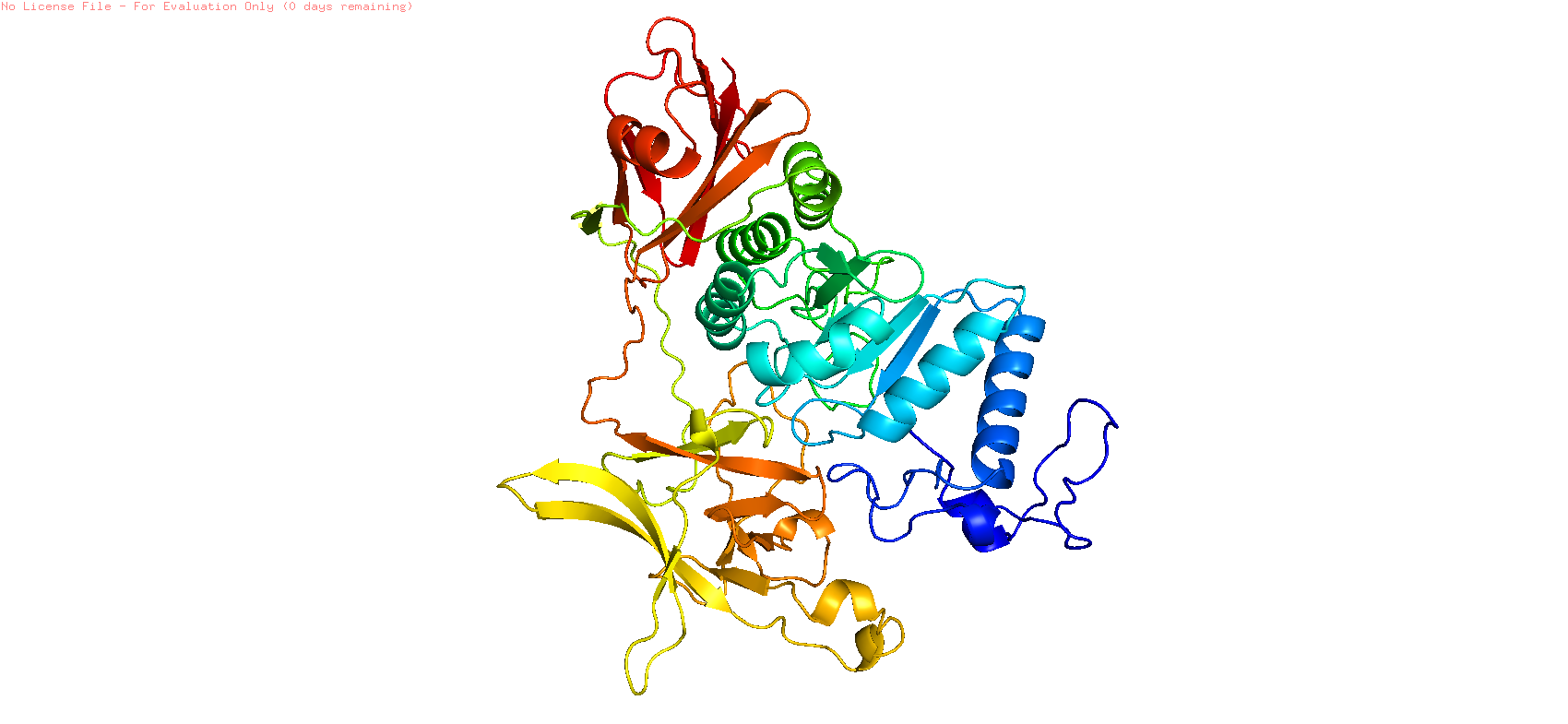  RMSD against OsAPSE: 1.469911 Å over 504 residues |
| *osapse9* | **M**GRGAPSSHPPPWRRLLRYALLCALLP  PWGTSEANEQLAEFLQN | pTM = 0.42  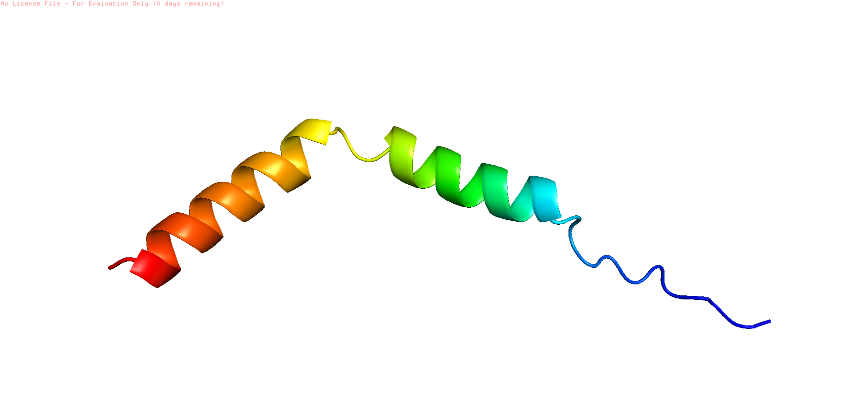 |
|  | **M**GLKFGIHLMKGISLQAVNGNTPILDI  KTGKPYVEDGRQWTARDIGLTHRTCAW  MPHGFMSVNTDIGAGKAFLRSLYQQYA  DWGVDFVKV**D**CIFGTDYSPKEIITISE  LLAELDRPIILSISPGTEVTPALAKNI  SQHVNMYRITG**D**DWDNWKDVSSHFDVS  SSFAAANKIGAIGLRGRSWPDLDMLPF  GWLTNAGVNQGPHRQCELTSDEQRTQI  ALWSMAKSPLMYGGDLRHLDNDTLSII  TNPTLLKINHYSINNMEFHHVHSERTS  KEDKHSSRFISEDLVHVPKIDGVSLGL  TACSDDKANGWYMFSQHGKSDHICRNY  GMQDDKNISFCLGKTIPLLTSDDIIVH  NEEYQTKFHLANMDSDDACLDASGSQR  RTSSDSKFPMFSRCRWHAMQMWELNEK  GNLISSYSRLCATVESNNKGVVTTGAV  ARAWIATGSKGEIYLAFFNLDSMSRKI  TARISDLEKVLGSTFIRKDTCSCTEVW  SGRNFGRVEEEISAVVKSHGSMVFEIT  C | pTM = 0.83  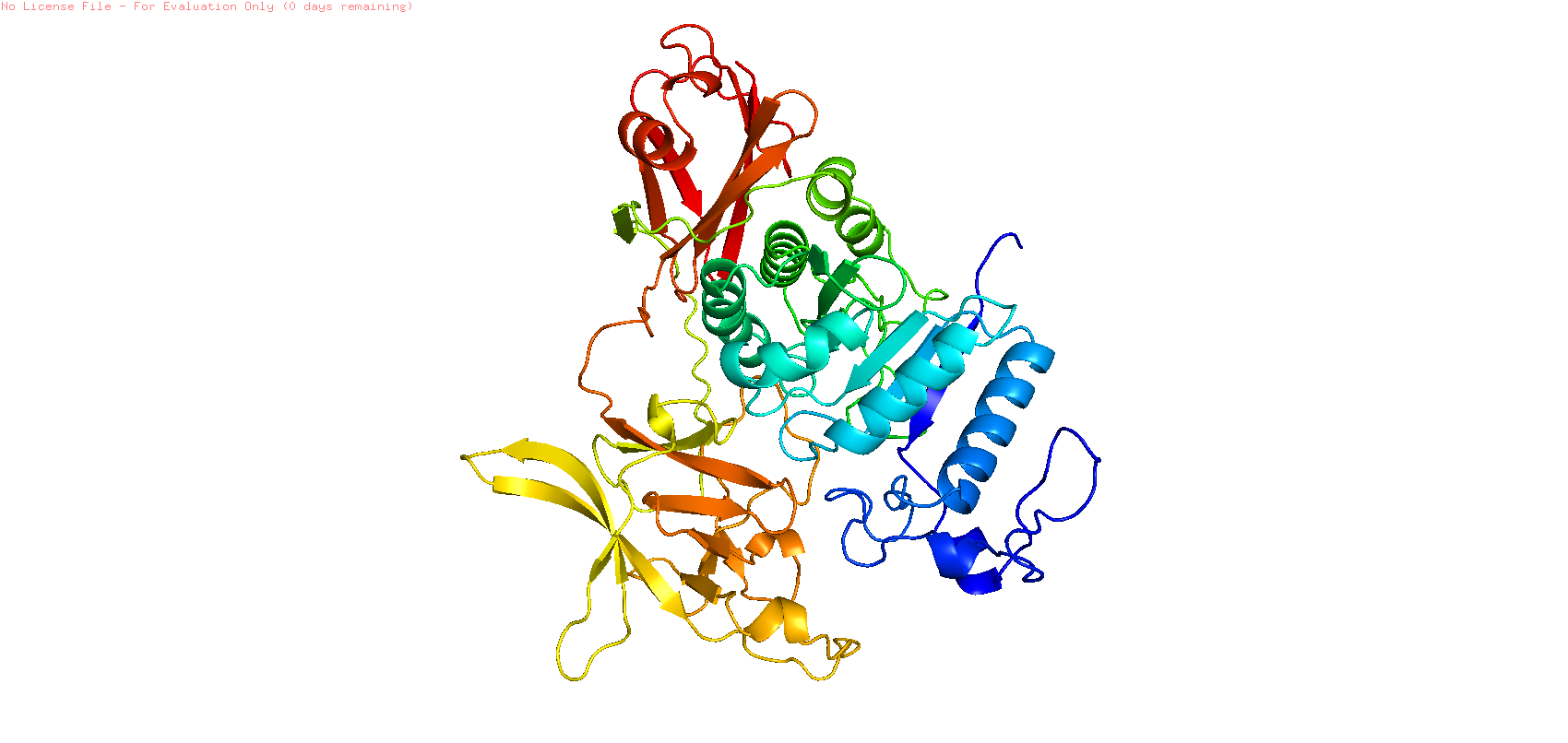  RMSD against OsAPSE: 2.066285 Å over 504 residues |

Mutant *osapse4/5* and *osapse9* give rise to a set of polypeptides with varying numbers of amino acid residues. The most likely polypeptides that originate from premature stop codons have a length ranging between 44 and 75 amino acids and do not contain binding sites. Structurally, these polypeptides resemble α-helices and random coils and are catalytically inactive. The theoretical polypeptides originating from alternative downstream start codons are longer (*i.e.* 505 – 514 residues) and contain the remaining OsAPSE coding sequence, including the catalytic residues D226 and D282, although it is not likely that these polypeptides will be created. The truncated versions of OsAPSE have lost 144 or 135 residues and are structurally different from OsAPSE, also assuming impaired functionality compared to the native OsAPSE (Illergård et al., 2009) (**Figure S10.2**).


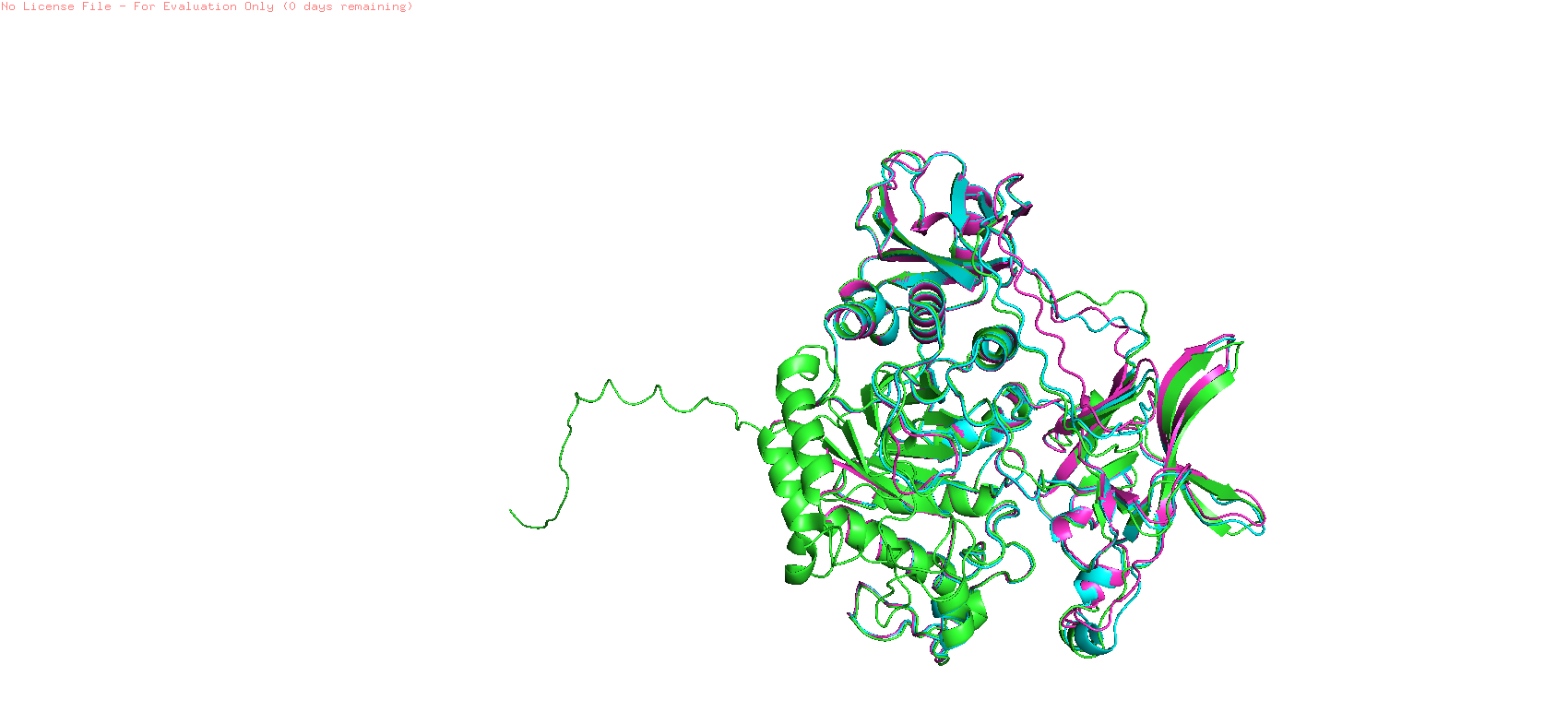


**Figure S10.2 – Structural alignment of OsAPSE (green) with truncated versions originated from KO mutations: *osapse4/5* (cyan) and *osapse9* (magenta).**

**References**

Arhab, Y., Bulakhov, A. G., Pestova, T. V., and Hellen, C. U. T. (2020). Dissemination of Internal Ribosomal Entry Sites (IRES) Between Viruses by Horizontal Gene Transfer. *Viruses* 12, 612. doi: 10.3390/v12060612

Cohen, S., Kramarski, L., Levi, S., Deshe, N., Ben David, O., and Arbely, E. (2019). Nonsense mutation-dependent reinitiation of translation in mammalian cells. *Nucleic Acids Res.* 47, 6330–6338. doi: 10.1093/nar/gkz319

De Wever, J., De Coninck, T., Everaert, H., Afoakwa, E. O., Coppieters, F., Rottiers, H., et al. (2021). Selection and validation of reference genes for accurate RT-qPCR gene expression normalization in cacao beans during fermentation. *Tree Genet. Genomes* 17, 7. doi: 10.1007/s11295-021-01490-2

Embree, C. M., Abu-Alhasan, R., and Singh, G. (2022). Features and factors that dictate if terminating ribosomes cause or counteract nonsense-mediated mRNA decay. *J. Biol. Chem.* 298, 102592. doi: 10.1016/j.jbc.2022.102592

Illergård, K., Ardell, D. H., and Elofsson, A. (2009). Structure is three to ten times more conserved than sequence—A study of structural response in protein cores. *Proteins Struct. Funct. Bioinforma.* 77, 499–508. doi: 10.1002/prot.22458

Kozak, M. (1990). Downstream secondary structure facilitates recognition of initiator codons by eukaryotic ribosomes. *Proc. Natl. Acad. Sci.* 87, 8301–8305. doi: 10.1073/pnas.87.21.8301
